# A thermophysical investigation of weakly coordinated metals in ionic liquids†

**DOI:** 10.1039/d4sc03588g

**Published:** 2024-07-31

**Authors:** Coby J. Clarke, Thomas Clayton, Matthew J. Palmer, Kevin R. J. Lovelock, Peter Licence

**Affiliations:** a GSK Carbon Neutral Laboratory, School of Chemistry, University of Nottingham Nottingham UK coby.clarke@nottingham.ac.uk; b Department of Chemistry, University of Reading Reading UK

## Abstract

Ionic liquids can solvate metals without strongly coordinating them, which gives a rare opportunity to probe the complexity of weakly coordinated metals through characterisation of liquid properties. In this work we use bis(trifluoromethanesulfonyl)imide (*i.e.* bistriflimide; [NTf_2_]^−^) anions to prepare weakly coordinated metal containing ionic liquids (MILs) that are highly versatile because they are reactive with readily substituted ligands. Weakly coordinated metals are more than highly active catalysts. They are primed to create dynamic systems that are useful in other areas such as battery electrolytes, soft materials, and separations. However, very little is known about the properties of ionic liquids with weakly coordinated metals, so we present a wide scope analysis of nineteen 1-alkyl-3-methylimidazolium bistriflimide ILs with five different M[NTf_2_]_*n*_ salts (M = Li, Mg, Zn, Co, Ni) in variable concentration to understand how metal cations influence thermophysical properties. We investigate short- and long-term thermal stability, decomposition kinetics, and decomposition mechanisms which provides operating windows and knowledge on how to improve stability. In particular, we find that all metals catalyse the elimination decomposition process, which severely compromises thermal stability. Alongside this, we present a detailed analysis of viscosities, densities, and heat capacities, the latter of which revealed that bistriflimide metal ILs are prone to drawing water from the air to form strong hydration spheres. Thermal parameters are affected to varying degrees, but desorption is possible under elevated temperatures — further justifying the need to know upper temperature limits. Altogether, this work provides a broad and methodical study to help understand solvent–solute interactions and thus design better systems for emerging applications that utilise weakly coordinated metals.

## Abstract 2

This wide scope study of weakly coordinated metals solvated in ionic liquids links molecular scale interactions to their macroscopic properties: thermal stability, decomposition mechanisms, viscosity, density, heat capacity, and glass transitions.

## Graphical abstract

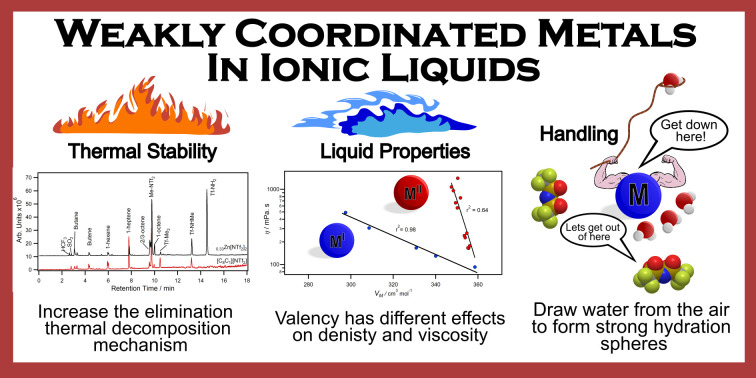

## 1. Introduction

Ionic liquids (ILs) are free-flowing salts with diverse molecular structures. They are highly solvating and capable of dissolving metal compounds to give mixtures known as metal containing ILs (MILs) that are useful as catalytic media. Recently, many metal bistriflimide salts (*i.e.*, M[NTf_2_]_*n*_, where *n* = 1 or 2 depending on oxidation state) have become commercially available. Dissolving M[NTf_2_]_*n*_ salts in [NTf_2_]^−^ ILs (here abbreviated [Cation][NTf_2_]_*χ*_M[NTf_2_]_2_, where *χ* = mole fraction of metal salt) results in highly Lewis acidic metals because they are solvated by weakly coordinating [NTf_2_]^−^ anions that are easily displaced.^1–7^ However, very little is known about the physical properties of weakly coordinated MILs and there is a significant gap in knowledge, especially for thermal properties which are important because most applications use elevated temperatures. The properties of mixtures can also be vastly different to their pure components, and metals are highly susceptible to poisoning from IL decomposition products which can accumulate over time.^8^ Therefore, overheating MILs could be detrimental to their use, so knowledge of operating temperatures is key to extending application lifetimes, which is particularly important for highly fluorinated molecules.

Currently, halometallate ILs are the most common examples of MILs, and they are primarily used as Lewis acid catalysts.^9^ However, halometallates have limited applications because strongly coordinating halides prevent ligand exchange, restricting the chemical transformation toolkit. For example, chlorozincate ILs are known to act as Lewis acids when bridging chloride ligands open along the polyanionic complex to accept Lewis bases, so most Zn–Cl bonds are retained.^10^ Applications of [Cation][NTf_2_]_*χ*_M[NTf_2_]_*n*_ MILs are currently appearing across vastly different fields, but there is a common theme arising — weakly coordinated metals give dynamic systems that introduce an extra level of tunability and functionality, opening up MIL applications beyond catalysis^11,12^ to areas such as energy applications,^13^ materials,^14^ and separations. Electrochemical energy-storage (EES) is a rapidly growing field where weakly coordinated MILs have great potential and the details of these systems have been discussed elsewhere,^15^ particularly with redox active Li^+^. Other metal-based EES systems for Na^+^, K^+^, Mg^2+^, Zn^2+^, and Al^3+^ are also being developed, and [NTf_2_]^−^ IL electrolytes play a significant role in enabling these technologies.^16^ Likewise, [NTf_2_]^−^ ILs are increasingly popular in solid polymer electrolytes where they decrease the interfacial resistance between Li metal and the polymers.^14,17^ However, not all applications relate to energy; [C_*n*_C_1_Im][NTf_2_] ILs containing Zn[NTf_2_]_2_ can be used to create soft ionic liquid gels (ionogels) with tuneable mechanical and surface compositions, guided through Zn^2+^–polymer interactions.^18^ Also, Ag^+^ ions in [C_*n*_C_1_Im][NTf_2_] ILs are so electrophilic that they can accept π-donor ligands which enables them to separate olefin/paraffins gases or liquids, leading to their adoption as GC stationary phases or in facilitated transport membranes (FTMs) for propylene/propane separation.^19,20^ In all these applications, the properties of ILs give key advantages over molecular solvents: highly solvating, conductive, non-flammable, effectively involatile, and importantly, thermal stable — but to what degree is currently unclear for their metal mixtures. Moreover, weakly coordinated metals are of broad interest outside of ILs and decades of research into non-coordinating anions has helped areas such as homogeneous catalysis and electrolytes significantly advance.^21–23^ Established applications could be enhanced by IL solvent effects as well as their physical properties, so there is also an opportunity to improve existing chemistry.

**Fig. 1:**
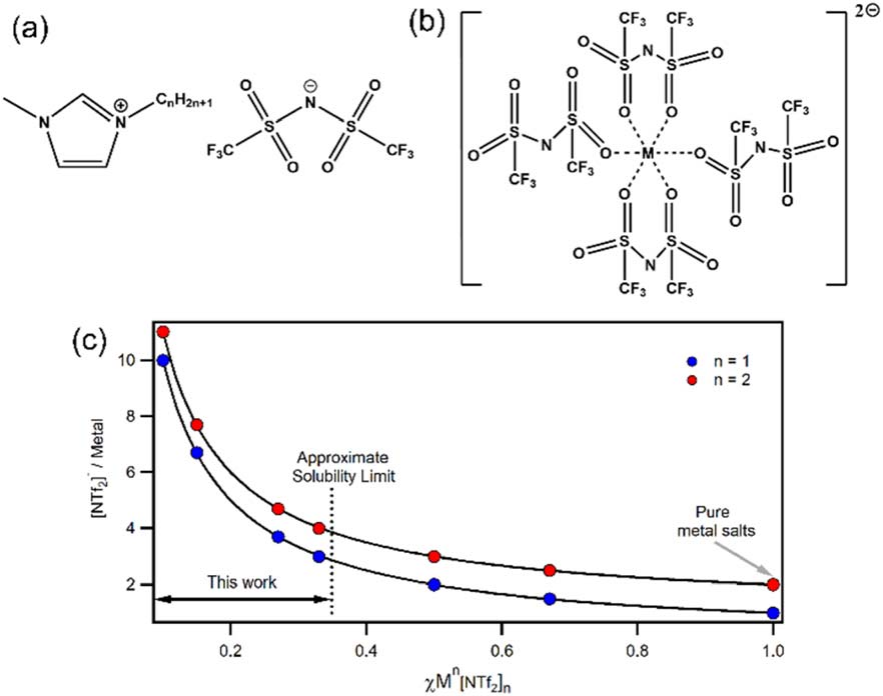
Structures of (a) 1-alkyl-3-methylimidazolium bistriflimide ILs; (b) the 4-fold coordination structure of M^II^[NTf_2_]_2_ salts as found from X-ray crystallography;^1^ (c) anion/metal ratio for monovalent (blue) and divalent (red) solutions of M^*n*^[NTf_2_]_*n*_ in [Cation][NTf_2_] ILs at various *χ*M^*n*^[NTf_2_]_*n*_.

Understanding the coordination environments of metal ions in [Cation][NTf_2_]_*χ*_M[NTf_2_]_2_ ILs is remarkably difficult, but nevertheless it is important for the development of structure–property relationships to improve and optimise function. In the solid state, crystal structures of divalent M[NTf_2_]_2_ salts (Fig. 1b) reveal 4-coordinate complexes with two bidentate ligands.^6^ Crystallography is not possible for IL mixtures so other techniques are needed. For dilute solutions of Zn[NTf_2_]_2_ in [NTf_2_]^−^ ILs, Zn K-edge extended X-ray absorption fine structure (EXAFS) and molecular dynamics (MD) simulations indicate the existence of an octahedral six coordinate [Zn(NTf_2_)_6_]^4−^ complex with monodentate [NTf_2_]^−^ anions coordinated solely through oxygen atoms,^24,25^ and similar six-coordinate species have also been identified for Co^2+^, Ni^2+^ and Mg^2+^.^7,26^ Hence, a maximum of 6 [NTf_2_]^−^ ligands are expected in divalent metal inner coordination spheres, which corresponds to *χ* = 0.2 (note: below this concentration excess [NTf_2_]^−^ anions will occupy outer spheres). For larger concentrations, fewer ligands are available so lower coordinate species may be present. Other studies have suggested that lower coordinate species such as [Zn(NTf_2_)_3_]^−^ can form;^27,28^ however there are few speciation studies at higher concentrations. To further complicate matters, [NTf_2_]^−^ may be able to coordinate through nitrogen but this is yet to be confirmed experimentally to the best of our knowledge.^29^ Raman spectroscopy has been used to study metal–[NTf_2_]^−^ coordination through shifts in the expansion and contraction mode of the [NTf_2_]^−^ anion.^30^ Deconvolution of the signals allows bound ligands and free anions in the bulk IL to be quantified, which enables coordination numbers to be calculated. Metals such as Li(i), Na(i), K(i), Cs(i), Mg(ii), Fe(ii), Co(ii), Ni(ii), Zn(ii), Ag(i), and Dy(ii/iii) have been studied and coordination numbers are generally smaller than those found by MD.^30–38^ Importantly, in our work the concentration range is sufficiently large (Fig. 1c) that the number of anions per metal cation span a wide range (10–3 for +1; 11–4 for +2) to give variable coordinated/solvated states. Calculations suggest that organic cation do not influence coordination of [NTf_2_]^−^ ligands around metal centres.^7^

Monovalent Li[NTf_2_] in [C_*n*_C_1_Im][NTf_2_] is thought to be coordinated by 2–4 [NTf_2_]^−^ anions.^39^ For *χ*Li[NTf_2_] < 0.2, a four-coordinate complex with bidentate [NTf_2_]^−^ anions has been suggested based on IR and Raman spectroscopy data.^40^ For *χ*Li[NTf_2_] > 0.2, the number of coordinated [NTf_2_]^−^ ligands was found to decrease below 2, supporting MD calculations that found nanostructured polymeric [Li_*m*_[NTf_2_]_*n*_]^(*n*−*m*)−^ aggregates (where *n* = 2*m* − 1) could be formed by bridging [NTf_2_]^−^ anions.^41^ Furthermore, calculations show a temperature dependence on clustering, with greater cluster sizes at lower temperatures. Borodin *et al.* have calculated that at 60 °C there are 4.7–4.8 [NTf_2_]^−^ anions coordinating Li^+^ and Na^+^, larger than previously thought at room temperature.^27^ The authors calculations also showed that the fraction of monodentate [NTf_2_]^−^ ligands increases with increasing temperature for monovalent Li^+^ and Na^+^ but decreases with increasing temperature for divalent Mg^2+^. Hence, the authors suggest aggregation of metal ions may change with increasing temperature because monodentate [NTf_2_]^−^ anions are more likely to bridge metals. Given the limited number of studies and the contradicting results published for weakly coordinating MILs, it is clear that speciation is more complex than halometallate analogues. Regardless, experimentally derived physical parameters are still needed to aid application development and help link structure to property.

In this work, we investigate thermal properties of nineteen [C_*n*_C_1_Im][NTf_2_]_*χ*_M[NTf_2_]_*n*_ ILs where M = Li, Mg, Zn, Co, Ni and *χ* = 0–0.33. We first investigate the effects of metal concentration on short and long-term thermal stability and then shows how metals influence decomposition rates through thermogravimetric analysis (TGA) kinetics. We next examine how metals effect decomposition mechanisms, which we have studied with 5 different analytical methods that were necessary due to the complexity of the samples. We present variable temperature density and viscosity data along with low temperature transitions and heat capacity from differential scanning calorimetry (DSC) measurements for the whole data set and discuss how different metals and different concentrations affect these respective properties. Hence, this study gives a wide scope perspective of the thermal properties of weakly coordinated metal ILs, which will help application development across a wide temperature range. The extensive list of thermal parameters published in this work are glass transition (*T*_g_), mass loss at 1% (*T*_1%_), onset temperature (*T*_onset_), liquid range, effective activation energy of decomposition (*E*_eff_), temperature at which 1% mass is lost over 10 hours (*T*_0.01/10_), heat capacity (*C*^o^_p_), density (*ρ*), and viscosity (*η*).

## 2. Results and discussion

### 2.1 Thermal stabilities and kinetics

**Fig. 2:**
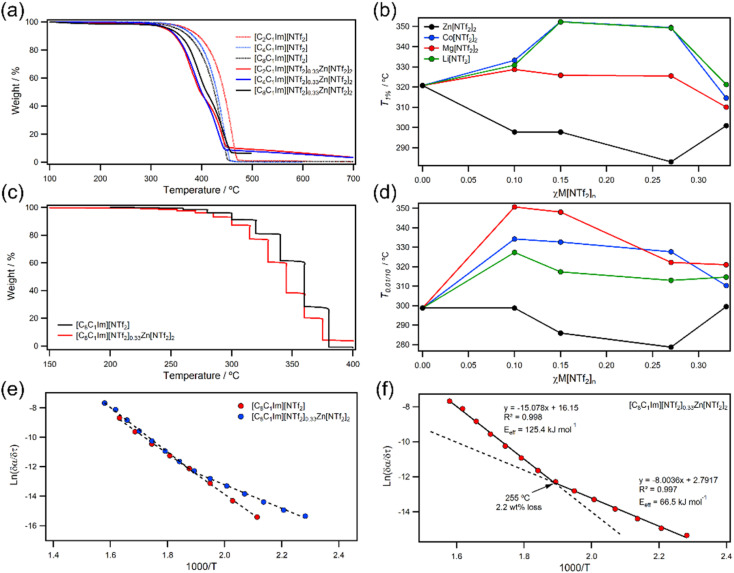
(a) Ramping TGA data for variable chain length [C_*n*_C_1_Im][NTf_2_]_0.33_Zn[NTf_2_]_2_ ILs; (b) *T*_1%_ short-term thermal stability *versus* mole fraction for [C_8_C_1_Im][NTf_2_]_*χ*_Zn[NTf_2_]_2_; (c) stepwise TGA profiles for [C_8_C_1_Im][NTf_2_] (red) and [C_8_C_1_Im][NTf_2_]_0.33_Zn[NTf_2_]_2_ (black) ILs; (d) long-term thermal stability *versus* mole fraction; (e) Arrhenius plots for [C_8_C_1_Im][NTf_2_] (red) and [C_8_C_1_Im][NTf_2_]_0.33_Zn[NTf_2_]_2_ (blue); (f) Arrhenius plot with two linear fits for [C_8_C_1_Im][NTf_2_]_0.33_Zn[NTf_2_]_2_.

We first decided to investigate thermal stabilities of [C_*n*_C_1_Im][NTf_2_]_*χ*_Zn[NTf_2_]_2_ ILs using TGA to see how different alkyl-chain lengths were affected by metals. Visually, ramping experiments appeared to show that Zn[NTf_2_]_2_ decreased the stability of all [C_*n*_C_1_Im][NTf_2_]_2_ ILs regardless of chain size (Fig. 2a; ESI, Table 1†); however, inspection of TGA derived parameters showed more intricate relationships. For example, the short-term parameter *T*_1%_ showed that Zn[NTf_2_]_2_ reduced thermal stability of short-chain ILs more than long-chain ILs. *T*_0.01/10_ values which parametrise long-term thermal stability also showed a significant decrease for ethyl-chains, while butyl- and octyl-chains remained consistent. In this work, *T*_0.01/10_ values were measured by stepwise isothermal TGA which significantly increased the rate of data collection and enabled many samples to be analysed (Fig. 2c; ESI, methods†) in a short period of time. The full list of thermal parameter values is given in the ESI† (ESI, Table S1†).

Moving to variable concentration experiments, we generally found *T*_1%_ values steadily increased for Li, Mg, and Co before decreasing at high *χ*M[NTf_2_]_*n*_ (Fig. 2b), although the effect was comparatively smaller for Mg. However, Zn showed an almost inverse trend, whereby stability substantially decreased and then began to increase at high *χ*Zn[NTf_2_]_2_. These trends were broadly reflected in *T*_0.01/10_ values (Fig. 2d), further validating our observations on a long-term scale. Usually, there is a reasonable correlation between long-term and short-term parameters (SI, Fig. S85†), but for MIL samples in this work it was less apparent. This deviation could be due to small decreases in mass before *T*_onset_, so to examine this we decided to investigate the rates of thermal decomposition using isothermal kinetics.

The Arrhenius plot of metal-free [C_8_C_1_Im][NTf_2_] decomposition gave a linear trend as previously measured for all aprotic metal-free ILs; however, metal containing [C_8_C_1_Im][NTf_2_]_0.33_Zn[NTf_2_]_2_ gave a non-linear relationship that could be described by two linear fits (Fig. 2e and f). This was found for most of the MILs in this work and all profiles showed an inflection point below 5 wt% at temperatures >240 °C. Arrhenius plots of this shape are generally indicative of two competing reaction pathways with different rates that give rise to a temperature-dependant activation energy.^42^ It is difficult to measure high and low conversion with isothermal kinetics; however, the two linear trends can give an effective activation energy (*E*_eff_) for each decomposition process. In this work, we find consistent results that show a low *E*_eff_ process at low temperatures leading to a comparatively higher *E*_eff_ process at higher temperature. For [C_8_C_1_Im][NTf_2_]_0.33_Zn[NTf_2_]_2_, the second decomposition step could be decomposition of [C_8_C_1_Im][NTf_2_] because it matches that of [C_8_C_1_Im][NTf_2_] in the Arrhenius plot (Δ*E*_eff_ = 7.1 kJ mol^−1^), but most other MILs have significantly higher gradients. Interestingly, *E*_eff_ values are somewhat metal dependant which suggests this step may involve metal centres in some way.

### 2.2 Decomposition mechanism

**Fig. 3:**
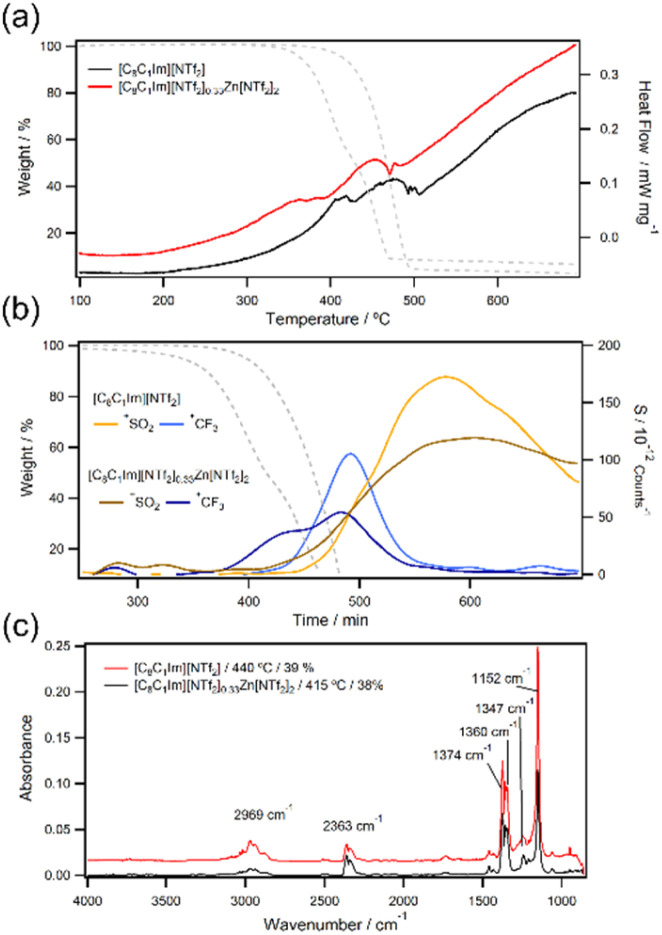
STA DSC profiles (a), TGA-MS data (b), and TGA-IR data (c) for [C_8_C_1_Im][NTf_2_] and [C_8_C_1_Im][NTf_2_]_0.33_Zn[NTf_2_]_2_.

To understand our observations and help interpret thermal stability parameters, we decided to conduct a mechanistic study. To date, a reliable decomposition pathway for the [NTf_2_]^−^ anion has not been proposed because it is extremely difficult to predict or detect decomposition products.^43–46^ Therefore, in this work we have also chosen to investigate metal-free [C_8_C_1_Im][NTf_2_] decomposition given the absence of control data. Firstly, we found there was no single mechanism dominating decomposition of either [C_8_C_1_Im][NTf_2_] or [C_8_C_1_Im][NTf_2_]_0.33_Zn[NTf_2_]_2_ as determined by the heat flow data from simultaneous thermal analysis (STA; Fig. 3a). This supports that multiple processes are occurring in parallel or sequence, unlike halide ILs or halometallate ILs which have one dominating *T*_d_ process.^8,47^ From TGA-MS data, [HCF_2_]^+^ (*m*/*z* 51) and [CF_3_]^+^ (*m*/*z* 69) ions were identified, which shows that fluoroform or other volatile hydrofluorocarbons are present in the decomposition vapours of both samples (Fig. 3b). [SO_2_]^+^˙ (*m*/*z* 64) was also identified but this signal appeared at higher temperatures than [CF_3_]^+^, so SO_2_ release is unlikely to be the leading decomposition step.^44^ This is supported by the observation that other thermally stable cations such [PPh_4_][NTf_2_] can have much higher decomposition temperatures than imidazolium ILs (ESI, Fig. S86†).^48^ TGA-IR data was primarily composed of SO_2_ as other studies have found (Fig. 3c), but this technique has an experimental bias to high volatile products that are strong IR-absorbers. TGA-MS is also biased to high volatiles such as HCF_3_ or SO_2_ because low and semi-volatiles are unlikely to be detected with these instrument configurations.

*Ex situ* analysis of [C_8_C_1_Im][NTf_2_] decomposition residues by high resolution ESI-MS (ESI, Fig. S179–S183†) showed [C_8_C_1_Im]^+^ as the primary constituent, but interestingly 13.5% octylimidazole (C_8_Im) was present. Alkylimidazoles are often found when imidazolium ILs with nucleophilic anions thermally degrade *via* the reverse Menshutkin reaction (*i.e.* nucleophilic substitution). Hence, this observation is unexpected given the weak nucleophilicity of [NTf_2_]^−^. Interestingly, [C_8_C_1_Im][NTf_2_]_0.33_Zn[NTf_2_]_2_ had significantly smaller amounts of C_8_Im at 2%, which was also unexpected because alkylimidazoles form strong Lewis adducts with Lewis acidic halometallate ions and are thus known to concentrate in decomposition residues.^8^ This observation hinted at a new decomposition pathway, but negative mode ESI-MS provided no other evidence as only [NTf_2_]^−^ ions were found in both [C_8_C_1_Im][NTf_2_] and [C_8_C_1_Im][NTf_2_]_0.33_Zn[NTf_2_]_2_ residues, so further investigation was needed.

**Fig. 4:**
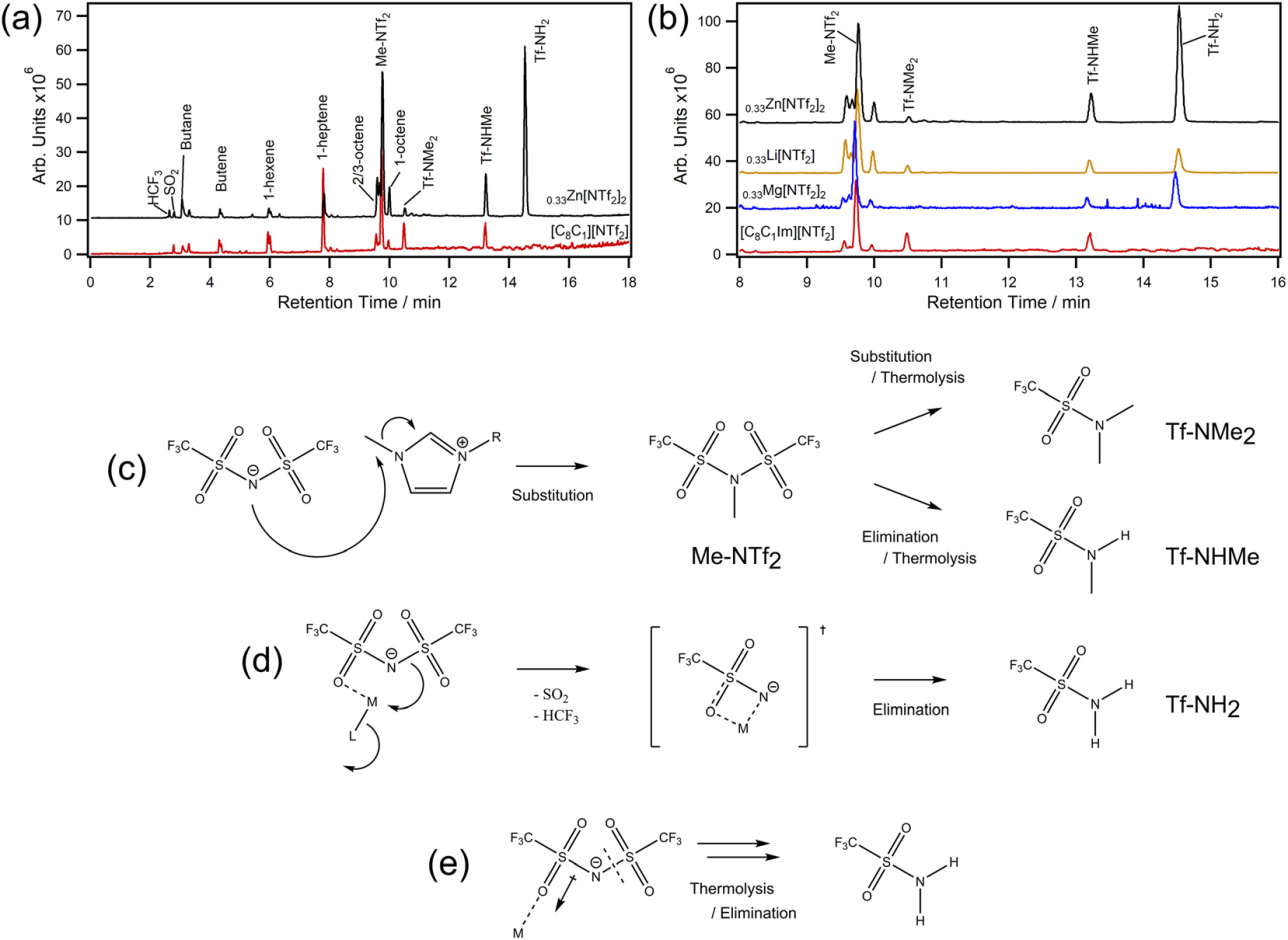
TD-GC-MS chromatograms for (a) [C_8_C_1_Im][NTf_2_] (red) and [C_8_C_1_Im][NTf_2_]_0.33_Zn[NTf_2_]_2_ (black) with peak assignments for 0–18 min; (b) chromatogram expansions from 8–16 min with [C_8_C_1_Im][NTf_2_]_0.33_Li[NTf_2_] and [C_8_C_1_Im][NTf_2_]_0.33_Mg[NTf_2_]_2_ for comparison; (c) decomposition scheme showing substitution and elimination routes in the absence of metals (a full scheme is shown in ESI, Scheme S1†); (d) metal–ligand interaction leading to formation of a transition state and subsequent elimination reactions; (e) simplified representation for polarisation of [NTf_2_]^−^ and the increased susceptibility to thermolysis.

We next chose to analyse samples by thermal desorption (TD) to better understand decomposition vapours (Fig. 4a and b). This technique works by collecting volatile products from a desorption tube on a focusing trap so they can be delivered onto a hyphenated GC-MS for separation and detection. Importantly, there are no condensation points and products can be concentrated over time. In our experiments, MILs were decomposed *in situ* at 400 °C and the evolved volatiles were collected over 20 minutes — decomposition rather than desorption. For [C_8_C_1_Im][NTf_2_], we found small amounts of expected volatiles such as SO_2_ and HCF_3_ along with various lengths of saturated and unsaturated hydrocarbons. However, methyl bistriflimide (Me-NTf_2_) was also detected in high quantity, which is direct evidence that [NTf_2_]^−^ can participate in a reverse Menshutkin-like reaction during thermal decomposition (Fig. 4c). Dimethyl triflamide (Tf-NMe_2_) and methyl triflamide (Tf-NHMe) were also detected which suggests Me-NTf_2_ can participate in further reverse substitution or elimination chemistry. Since [C_8_C_1_Im][NTf_2_] can be described by single step kinetic models,^49^ one of these reactions will be the rate limiting step that leads to mass loss, which could be why the *E*_a_ (114.4 ± 1.4 kJ mol^−1^) is lower than that of [C_8_C_1_Im]Cl (133.1 ± 5.7 kJ mol^−1^) despite the ≈ 130 °C increase in *T*_1%_.^8^

For [C_8_C_1_Im][NTf_2_]_0.33_Zn[NTf_2_]_2_, similar decomposition products were found to those of [C_8_C_1_Im][NTf_2_], however large amounts of triflamide (Tf-NH_2_) were also detected. Importantly, this product was found for all MIL samples in this work, but not [C_8_C_1_Im][NTf_2_]. The Tf-NH_2_ product is most likely produced through elimination mechanisms with protons of the octyl-chain, which is supported by the observed increase in octene isomers (a full scheme is shown in ESI, Scheme S1†). Given the mixture of Me-NTf_2_, Tf-NMe_2_, Tf-NHMe, and Tf-NH_2_, it is likely that this process occurs in parallel with nucleophilic substitution and these two processes are thus the ones identified in the kinetic study. Relative to Me-NTf_2_ peak areas, the quantity of Tf-NH_2_ increased in the order Li < Mg < Zn ≪ Co. The large amount of Tf-NH_2_ detected from the Co MIL (ESI, Fig. S177†) shows that open shell metals are far more active than closed shell metals which highlights that transition metals containing ILs are far more susceptible to poisoning from thermal decomposition. We did not find evidence of carbene formation in our analytical data. Carbenes usually form in the presence of bases,^50^ with metal oxides,^51^ in vacuuo,^52,53^ or from an applied potential difference^54^ (*e.g.* corona discharge in mass spectrometry).

For halozincate ILs, thermal stability strongly correlates to Lewis acidity, which is reflected in the M–Cl dissociation energies.^8^ For bistriflimide MILs, increasing concentration is known to increase Lewis acidity of metals, which could restrict ligand exchange and kinetically limit [NTf_2_]^−^ participating as a nucleophile in the substitution reaction. Theoretically, this would lead to a constant increase in stability; however, the decrease in stability observed at high *χ*M[NTf_2_]_*n*_ suggests other effects may be playing a role. For example, metal centres could be stabilising a molecular fragment (Fig. 4d) which is more likely to participate in elimination reactions due to sterics. Likewise, polarisation of [NTf_2_]^−^ ions may promote thermolysis by weakening N–S bonds (Fig. 4e). Regardless, a change in the decomposition pathways is insufficient to explain the trends observed in Fig. 2 alone, so nanostructure could also play a role. For example, increasing the concentration of Li[NTf_2_] has been shown to increase the structuring of non-polar domains in short-chain ILs.^55^ However, the opposite is found in long-chain ILs (*i.e.*, C_8_), where cations are increasingly disordered by disrupting aliphatic-domains which increases charge alternation. This could explain why short-chain ILs have larger reductions in thermal stability and why the *T*_1%_ and *T*_0.01/10_ of long-chain MILs decrease with increasing concentration — cations and anions are held in closer proximity. Furthermore, low concentrations of metal (*i.e. χ* = 0.1) can have large effects on nanostructural organisations, which could explain why we see large increases in *T*_0.01/10_ for Li, Co, and Mg at low metal content, and the slow decrease in stability as metal increases.

### 2.3 Viscosity and density

**Fig. 5:**
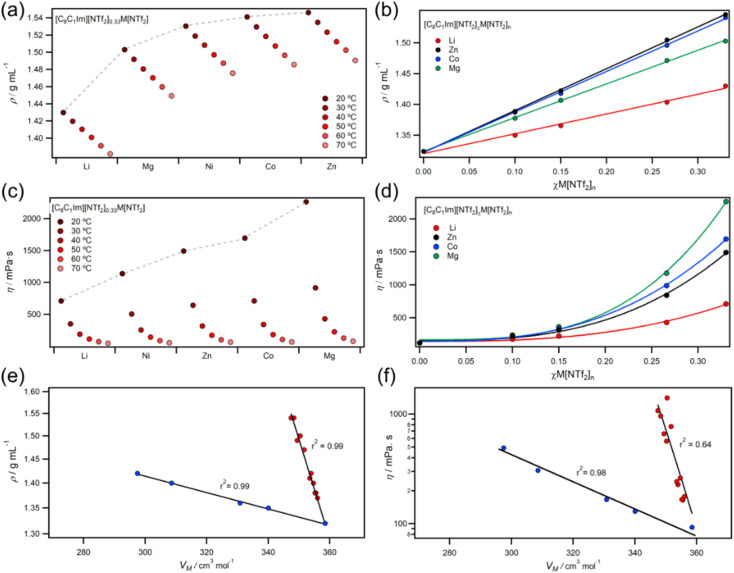
(a) Density data for [C_8_C_1_Im][NTf_2_]_0.33_M[NTf_2_]_*n*_ ILs; (b) density data for different mole fractions of [C_8_C_1_Im][NTf_2_]_0.33_M[NTf_2_]_*n*_ ILs; (c) viscosity data for [C_8_C_1_Im][NTf_2_]_0.33_M[NTf_2_]_*n*_ ILs; (d) viscosity data for different mole fractions of [C_8_C_1_Im][NTf_2_]_0.33_M[NTf_2_]_*n*_ ILs; (e and f) correlations with molar volume for density (e) and viscosity (f) (note: individual fits for MILs all had good correlations with *R*^2^ > 0.98).

Compared to molecular liquids, ILs have higher densities and viscosities because of coulombic charges which compact ions and increase molecular friction.^56^ In this work, MIL experimental densities followed the order Li < Mg < Ni < Co < Zn, while viscosities followed a different order of Li < Ni < Zn < Co < Mg (Fig. 5a and c). Elsewhere, viscosities have been calculated to increase in a similar order (Na < Li < Zn < Mg),^57^ and experimental studies on group 1 metals have linked small trends in viscosity to ionic radii.^58,59^ Temperature dependent viscosities of MILs were well described by the Vogel–Fulcher–Tammann (VTF) model (ESI, Fig. S202–S218†), which is known to better fit IL viscosities than the Arrhenius relationship.^60,61^ Interestingly, the Vogel parameter *T*_0_ (the temperature at which free volume available for hole formation is zero^62^) was found to obey a power-law dependency with concentration, but the pseudo-activation energy parameter B did not (ESI, Fig. S220–S221†). This suggests that free volume is primarily controlling viscosity in the temperature region measured, rather than activation energy.^63^ Conversely, cellulose solutions in chloride and acetate ILs have strong pseudo-activation energy power-law dependencies,^64,65^ which is most likely due to extensive hydrogen bonding networks.

Increasing the concentration of M[NTf_2_]_*n*_ salts in ILs led to linear increases in density but exponential increases in viscosity (Fig. 5b and d). Plots of viscosity and density against molar volume (*V*_M_) showed two clear trends for +1 and +2 salts (Fig. 5e and f), which was also found for molar concentration plots (Fig. S222†) — eliminating any issues from using the mole fraction weighted molecular weights. Viscosity is known to scale with *V*_m_ and density,^61,66,67^ but different cations do not usually cause deviations from these trends. Our observations could be due to differences in ligand exchange rates around metals since they are 10–50 times slower for divalent metals such as Mg^2+^ and Zn^2+^ than monovalent metals such as Li^+^.^27^

### 2.4 Adventitious water, glass transition, and heat capacity

**Fig. 6:**
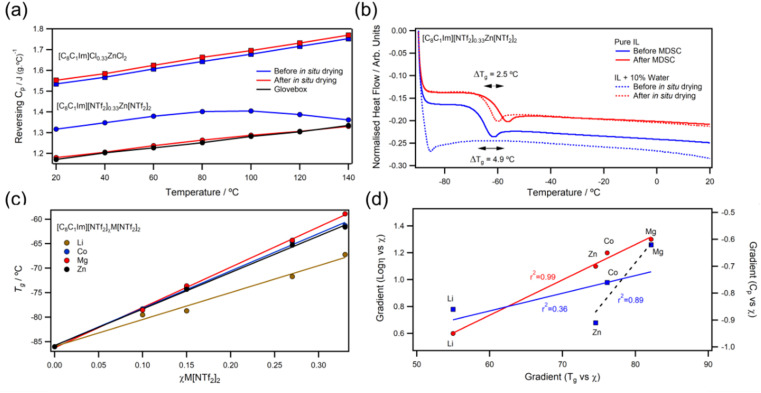
(a) QI-MDSC experiments for [C_8_C_1_Im][NTf_2_]_0.33_Zn[NTf_2_]_2_ (circles) and [C_8_C_1_Im]Cl_0.33_ZnCl_2_ (squares) before (blue) and after (red) *in situ* drying; (b) DSC data for pure [C_8_C_1_Im][NTf_2_]_0.33_Zn[NTf_2_]_2_ and a 10% water mixture; (c) *T*_g_*versus χ*M[NTf_2_]_*n*_ and (d) correlations of gradients taken from linear fits of logarithm of viscosity (20 °C; left axis; red circles), glass transition (bottom), and heat capacity (20 °C; right axis; blue squares) against mole fraction of metal salts.

For all metals, *T*_g_ correlated linearly with *χ*M[NTf_2_]_*n*_ (Fig. 6c) and linear fits gave gradients in the order Mg > Co > Zn ≫ Li. Ratios of *T*_g_/*T*_0_ deviated from those of ideal fluids but were consistent for all *χ*, suggesting a low rate of increase in free volume with temperature. Measured *C*^o^_p_ values were found to decrease with increasing *χ*M[NTf_2_]_*n*_, which is most likely due to the decrease in polyatomic [C_8_C_1_Im]^+^ cations. However, different metals reduced *C*^o^_p_ at different rates; linear fits to *C*^o^_p_*vs. χ* plots yielded gradients with the order Zn > Li > Co > Mg (ESI, Fig. S231†). Gradients from linear fits of the logarithm of viscosity *vs. χ* correlated with the those of *T*_g_, but not the with those of *C*^o^_p_ (Fig. 6d). Interestingly, better correlations were found for heat capacity when separating +2 ions from +1 (ESI, Fig. S233†), which could suggest that valency has different effects on rotational/vibrational and translational modes. For example, divalent metal ions are thought to increase ordering of ILs;^26^ and there is growing evidence that Zn^2+^ and Mg^2+^ may restrict rotation of [NTf_2_]^−^ ions to favour *cis*-conformers, which does not occur for monovalent ions.^25,58,68^ However, publications utilising Raman spectroscopy report conflicting results for conformations of metal bound [NTf_2_]^−^ anions,^31–38^ so further investigations are necessary.

## 3. Conclusions

We have thermally characterised a wide range of MILs with [NTf_2_]^−^ anions to understand how weakly coordinated metal ions influence properties. Short- and long-term thermal stability do not follow the previously observed increases for strongly coordinated halometallates and instead show more intricate relationships because of the weak dynamic bonding of [NTf_2_]^−^ ions. Kinetic studies revealed a non-linear temperature dependant activation energy that was indicative of two competing decomposition processes. Mechanistic studies identified these as substitution and elimination processes, which also both occur in metal-free [NTf_2_]^−^ ILs, but metal ions increase the rate of the elimination pathway. This is thought to proceed through a metal stabilised decomposition fragment which will reduce Lewis acidity. However, this change in mechanism is insufficient to explain the trends in concentration dependent stability plots. Changes in nanostructure may explain the discrepancies because low concentrations of metal can create extensive structuring that eventually leads to increased charge alternation as the amount of metal increases. Metal ions held in closer proximity to anions are more likely to react, but more studies on MIL structuring are needed to help separate this contribution, especially at elevated temperatures.

Correlations of VTF fitting parameters with concentration suggest that viscosity of metal ions is primarily controlled by free volume, rather than activation energy. Furthermore, molar volume plots revealed a strong oxidation state dependency for volumetric and flow properties, which are most likely due to higher charge densities and slower exchange rates, with ionic radii playing a more subtle role. Viscosity, glass transition and heat capacity correlations also suggest that valency has different effects on vibrational/rotational *vs.* translations modes which are exhibited through thermal parameter changes. In addition, while analysing heat capacities we noted that [NTf_2_]^−^ MILs were in fact hydrophilic and would readily draw water from atmospheric air. MILs could be dried *in situ*, but long drying times and high temperatures were needed because the highly Lewis acidic metals formed strong hydration spheres with water.

Weakly coordinated metal ions are growing in popularity because they have exceptional catalytic properties, and they enable dynamic chemistries. Hence, the results of this methodical study will contribute to many different areas. For example, β-substituted cations could significantly increase the stability of [NTf_2_]^−^ MILs by restricting the elimination decomposition pathway which would improve operating ranges for catalysts or high temperature batteries. Extending lifetimes is particularly important when fluorinated molecules have unmatched function and cannot be replaced. Furthermore, the mechanistic insight is important for these areas as it will enable the design of more robust systems. Solvent–solute interactions are also of relevance to many processes, so the molecular interaction insights presented in this work are of general importance, especially for the development of weakly coordinated systems.

## Data availability

All supporting data is available in the ESI† document.

## Author contributions

CJ: conceptualisation, investigation, formal analysis, writing; TC: methodology (TD-GCMS); MJP: investigation (synthesis); KRJL: conceptualisation; PL: funding acquisition, resources.

## Conflicts of interest

There are no conflicts to declare.

## Supplementary Material

SC-015-D4SC03588G-s001
