# Supplementary material for: A thermophysical investigation of weakly coordinated metals in ionic liquids
Source: Chem Sci. 2024 Jul 31;15(34):13832–40. doi: 10.1039/d4sc03588g (PMC11351778; doi:10.1039/d4sc03588g)
Supplement: SC-015-D4SC03588G-s001 [file SC-015-D4SC03588G-s001.pdf]

Supplementary Information (SI) for Chemical Science.
This journal is © The Royal Society of Chemistry 2024

Supporting Information

A Thermophysical Investigation of Weakly Coordinated Metals in Ionic Liquids

Coby J. Clarke,*[a] Thomas Clayton,[a] Matthew J. Palmer,[a] Kevin R. J. Lovelock[b] and Peter Licence[a]

(a) Department of Chemistry, The University of Nottingham, Nottingham, UK
(b) Department of Chemistry, University of Reading, Reading, UK

*Correspondence: coby.clarke@nottingham.ac.uk

1. Experimental

Synthesis and Analysis

Anhydrous metal bis(trifluoromethanesulfonyl)imide salts ($M[NTf_2]_n$; ≥ 99.9 % purity) were purchased from either Solvionic or Sigma Aldrich and dried on a Schlenk line at <2 x 10^{-2} mbar and 70 °C for at least 48 hr before being loaded and stored in an MBraun LABstar glovebox (< 0.5 ppm O_2 and < 0.5 ppm H_2O). 1-Butyl-3-methylimidazolium chloride ($[C_4C_1Im]Cl$) was prepared according to published procedures.[1] 1-octyl-3-methylimidazolium chloride ($[C_8C_1Im]Cl$), 1-octyl-3-methylimidazolium bromide ($[C_8C_1Im]Br$), and 1-Ethyl-3-methylimidazolium bis(trifluoromethanesulfonyl)imide ($[C_2C_1Im][NTf_2]$) were purchased from Sigma Aldrich. All other bis(trifluoromethanesulfonyl)imide ionic liquids (ILs) were prepared by ion metathesis from halide ILs in 18.2 MΩ cm^{-1} ultra-pure water from lithium bis(trifluoromethanesulfonyl)imide (1.2 equivalents) according to existing published procedures. Residual halide content was measured by ion chromatography (IC) and all ILs were found to contain less than 1.4 ppm halides (the detection limit of the IC).[2] Purity was also assessed by ^{1}H, ^{13}C, and ^{19}F NMR before ILs were used. All ionic liquids were dried on a Schlenk line at <2 x 10^{-2} mbar and 70 °C for 48-72 hr before being loaded into the same glovebox. Metal ionic liquids were prepared by weighing the calculated amounts into screw top glass vials which were stirred and heated to 70 °C for 24-48 hr until clear solutions were obtained. A detailed method has been published elsewhere.[3] Metal ILs were analysed by ^{1}H, ^{13}C, and ^{19}F NMR after preparation to ensure solutions had not decomposed during heating.

NMR was measured using a Bruker AV(III)400HD spectrometer equipped with a 5 mm autotune broadband probe with z gradients. Samples were dissolved in deuterated solvents (usually DMSO-d_6) to approximately 20-30 mg mL^{-1} concentration and spectra were referenced to the residual solvent peaks in Mestrenova before processing. Differential scanning calorimetry (DSC) was carried out with a TA Instruments Discovery DSC2500 with RCS-90 chiller. Approximately 3-5 mg of sample was used for standard experiments and the instrument was calibrated before use (baseline conditioning, temperature calibration, cell constant, reversing heat capacity calibration, and MDSC calibration with indium and sapphire calibration standards). Dry nitrogen gas was used for all experiments at 50 mL min^{-1} and samples were sealed in pre-weighed Tzero alodined hermetic pans in a glovebox and removed one at a time for analysis. Preliminary experiments were done in pin hole pans to allow an initial drying step (110 °C for 60 min) to drive water from the samples (i.e., *in situ* drying) and to erase the thermal history. Glass transition temperatures (T_g) were measured from thermograms with 10 °C min^{-1} heating rates. Heat capacities (C_p^o) were measured with approximately 15-20 mg of glovebox sealed sample. Quasi-isothermal modulated DSC (QIMDSC) was used to C_p^o values which were recorded in 20 °C increments from 20 °C to 140 °C. measure. A temperature amplitude of 1.00 °C and period = 120 s was used for temperature modulation and 10-minute isothermals steps were used to acquire C_p^o. To assess reproducibility, three separate sample pans containing $[C_8C_1Im][NTf_2]$ were analysed and the C_p^o at 25 °C was 714.0 ± 0.8 J mol^{-1} $°C^{-1}$ and the ΔC_p^o from 140 to 25°C was calculated to be 98.1 ± 1.2 J mol^{-1} $°C^{-1}$ (note: the error is the standard deviation of the average values; for ΔC_p^o error was propagated by the square root of the sum of the squares). The low standard deviation values showed that the measurements had high reproducibility and low error. Thermogravimetric analysis (TGA) was acquired using a TA Instruments Discovery TGA with high temperature Pt pans and nitrogen flow rate of 50 mL min^{-1}. For ramping experiments, samples were dried *in situ* for 60 minutes at 110-140 °C before heating at a rate of 10 °C min^{-1}.

Kinetic analysis was carried through either modulated TGA (MTGA) or an isothermal method. For MTGA, a period of 200 s and a modulation temperature of 5 °C was used. Hi-Res ramping at 2 °C min^{-1} with a resolution of 6.00 and sensitivity of 1.00 was also used to improve data quality. Activation energies (E_a) were calculated centred around 10 % mass loss over a 20 min window. The isothermal method used a stepwise temperature programme that increased in 15 °C increments every 45-60 minutes. The isothermal data was analysed according to previous methods to calculate the Arrhenius activation energies and $T_{0.01/10}$ values.

Dynamic viscosity, kinematic viscosity, and density were measured using an Anton Paar SVM 3000 Viscometer in 5 °C or 10 °C increments from 20-70 °C. Approximately 3 mL of IL was taken up into a syringe in a glovebox, which was sealed in nitrogen, removed from the glovebox and then injected directly into the instrument through a septum within 1 minute. Reproducibility was measured using triplicate injections of $[C_4C_1Im][NTf_2]$ and errors were found to be 0.24 % viscosity, 0.32 % kinematic, and 0.0237 g cm^{-3} for density. Three coefficient Vogel-Fulcher-Tammann (VFT) fittings were calculated in Igor Pro by non-linear least squares data fitting the expression:

$$Ln(\eta) = A + \frac{B}{T-T_O} \qquad \textbf{(Equation 1)}$$

Where η = dynamic viscosity, T = temperature, A is a constant and taking e^A = gives A_η the viscosity at infinite temperature, B is a constant and related to the Arrhenius activation energy[4] (i.e., a pseudo-activation energy), and T_O is a constant and termed the Vogel temperature. The Fragility parameter, D, was calculated from B/T_O and this parameter describes how rapidly dynamics of a liquid slow down as temperature approaches the glass transition — smaller D means viscosity is sensitive to temperature changes which is termed “fragile behaviour”, but large D means less sensitive to temperature, and this is termed “strong behaviour”. Strong liquids obey Arrhenius kinetics better than fragile liquids at low temperatures. Ionic liquid physical properties correlate very well to their molecular or molar volumes, which are both calculated from their respective ionic volumes.[5] In this work, molar volumes (V_m) of MILs were calculated from ionic volumes weighted according to χ and values for IL ions were taken from published reference tables.[6] The ionic volumes of metals were assumed to be the same as those in the metallic state so were calculated from the molar volumes of pure metals[7] at 25 °C, similar to other work.[8] The molar volume correlation factor published by Marcus[6] was used to account for deviations between IL calculated and experimental molar volumes by multiplying the obtained values by 689.5 rather than 602.2:

$$V_m = 689.5 * ((1-\chi)(V^+_{cation} + V^-_{anion}) + \chi(V^{n+}_{metal} + nV^-_{anion})) \qquad \textbf{(Equation 2)}$$

NMR Reports

$[C_2C_1Im][NTf_2]$: ^{1}H NMR (400 MHz, DMSO) δ = 9.10 (d, *J* = 1.7 Hz, 1H), 7.77 (t, *J* = 1.8 Hz, 1H), 7.68 (t, *J* = 1.8 Hz, 1H), 4.18 (q, *J* = 7.3 Hz, 2H), 3.84 (s, 3H), 1.41 (t, *J* = 7.3 Hz, 3H). ^{13}C NMR (101 MHz, DMSO) δ = 136.49, 123.61, 122.26, 119.50 (q, *J* = 321.9 Hz), 48.79, 35.74, 31.16, 29.38, 28.47, 28.33, 25.49, 22.05, 13.91. ^{19}F NMR (376 MHz, DMSO) δ = -78.73.

$[C_2C_1Im][NTf_2]_{0.33}Zn[NTf_2]_2$: ^{1}H NMR (400 MHz, DMSO) δ = 9.10 (s, 1H), 7.77 (t, *J* = 1.8 Hz, 1H), 7.68 (t, *J* = 1.8 Hz, 1H), 4.19 (q, *J* = 7.3 Hz, 2H), 3.84 (s, 3H), 1.42 (t, *J* = 7.3 Hz, 3H). ^{13}C NMR (101 MHz, DMSO) δ = 136.27, 123.61, 122.00, 119.53 (q, *J* = 321.9 Hz), 44.17, 35.72, 15.10. ^{19}F NMR (376 MHz, DMSO) δ = -78.74.

$[C_4C_1Im]Cl$: ^{1}H NMR (400 MHz, DMSO) δ = 9.24 (d, *J* = 1.8 Hz, 1H), 7.80 (t, *J* = 1.8 Hz, 1H), 7.73 (t, *J* = 1.8 Hz, 1H), 4.17 (t, *J* = 7.2 Hz, 2H), 3.85 (s, 3H), 1.82 – 1.70 (m, 2H), 1.26 (h, *J* = 7.4 Hz, 2H), 0.90 (t, *J*

= 7.4 Hz, 3H). ^{13}C NMR (101 MHz, DMSO) δ = 136.58, 123.60, 122.26, 48.44, 35.73, 31.35, 18.76, 13.27.

[C_4C_1Im][NTf_2]: ^{1}H NMR (400 MHz, DMSO) δ = 9.09 (d, *J* = 1.8 Hz, 1H), 7.76 (t, *J* = 1.8 Hz, 1H), 7.69 (d, *J* = 1.9 Hz, 1H), 4.15 (t, *J* = 7.2 Hz, 2H), 3.84 (s, 3H), 1.82 – 1.70 (m, 2H), 1.26 (h, *J* = 7.4 Hz, 2H), 0.90 (t, *J* = 7.4 Hz, 3H). ^{13}C NMR (101 MHz, DMSO) δ 136.49, 123.62, 122.26, 121.08, 117.88, 48.49, 35.73, 31.34, 18.76, 13.24.

[C_4C_1Im][NTf_2]$_{0.33}$Zn[NTf_2]$_2$: ^{1}H NMR (400 MHz, DMSO) δ = 9.09 (s, 1H), 7.75 (t, *J* = 1.8 Hz, 1H), 7.68 (t, *J* = 1.8 Hz, 1H), 4.15 (t, *J* = 7.2 Hz, 2H), 3.84 (s, 3H), 1.82 – 1.69 (m, 2H), 1.32 – 1.18 (m, 2H), 0.90 (t, *J* = 7.4 Hz, 3H). ^{13}C NMR (101 MHz, DMSO) δ = 136.53, 123.65, 122.29, 119.52 (q, *J* = 321.8 Hz), 48.54, 35.76, 31.38, 18.79, 13.26. ^{19}F NMR (376 MHz, DMSO) δ = -78.73.

[C_8C_1Im]Cl: ^{1}H NMR (400 MHz, DMSO) δ = 9.27 (d, *J* = 1.8 Hz, 1H), 7.80 (t, *J* = 1.8 Hz, 1H), 7.73 (t, *J* = 1.8 Hz, 1H), 4.16 (t, *J* = 7.2 Hz, 2H), 3.85 (s, 3H), 1.77 (p, *J* = 7.3 Hz, 2H), 1.32 – 1.15 (m, 10H), 0.89 – 0.80 (m, 3H). ^{13}C NMR (101 MHz, DMSO) δ = 136.58, 123.59, 122.26, 48.72, 35.73, 31.16, 29.39, 28.48, 28.34, 25.49, 22.06, 13.95.

[C_8C_1Im][NTf_2]: ^{1}H NMR (400 MHz, DMSO) δ = 9.09 (d, *J* = 1.8 Hz, 1H), 7.75 (t, *J* = 1.8 Hz, 1H), 7.69 (t, *J* = 1.8 Hz, 1H), 4.14 (t, *J* = 7.2 Hz, 2H), 3.84 (s, 3H), 1.77 (p, *J* = 7.2 Hz, 2H), 1.25 (q, *J* = 12.4, 9.1 Hz, 10H), 0.86 (t, *J* = 6.8 Hz, 3H). ^{13}C NMR (101 MHz, DMSO) δ = 136.48, 123.60, 122.25, 119.48 (q, *J* = 321.9 Hz), 48.78, 35.72, 31.14, 29.36, 28.45, 28.31, 25.48, 22.03, 13.88. ^{19}F NMR (376 MHz, DMSO) δ = -78.75.

[C_8C_1Im][NTf_2]$_{0.1}$Li[NTf_2]: ^{1}H NMR (400 MHz, DMSO) δ = 9.09 (s, 1H), 7.75 (d, *J* = 1.7 Hz, 1H), 7.68 (d, *J* = 1.7 Hz, 1H), 4.14 (t, *J* = 7.2 Hz, 2H), 3.84 (s, 3H), 1.77 (p, *J* = 7.2 Hz, 2H), 1.26 (q, *J* = 5.4, 4.9 Hz, 11H), 0.85 (t, *J* = 6.6 Hz, 3H). ^{13}C NMR (101 MHz, DMSO) δ = 136.49, 123.60, 122.25, 119.49 (q, *J* = 321.9 Hz), 48.79, 35.72, 31.15, 29.37, 28.46, 28.32, 25.49, 22.04, 13.88. ^{19}F NMR (376 MHz, DMSO) δ = -78.76.

[C_8C_1Im][NTf_2]$_{0.15}$Li[NTf_2]: ^{1}H NMR (400 MHz, DMSO) δ = 9.09 (s, 1H), 7.74 (d, *J* = 1.8 Hz, 1H), 7.67 (d, *J* = 1.8 Hz, 1H), 4.15 (t, *J* = 7.2 Hz, 2H), 3.85 (s, 3H), 1.78 (p, *J* = 7.2 Hz, 2H), 1.34 – 1.17 (m, 10H), 0.85 (t, *J* = 6.7 Hz, 3H). ^{13}C NMR (101 MHz, DMSO) δ = 136.51, 123.60, 122.26, 119.53 (q, *J* = 321.9 Hz), 48.83, 35.70, 31.17, 29.40, 28.48, 28.34, 25.50, 22.05, 13.83. ^{19}F NMR (376 MHz, DMSO) δ = -78.84.

[C_8C_1Im][NTf_2]$_{0.27}$Li[NTf_2]: ^{1}H NMR (400 MHz, DMSO) δ = 9.09 (s, 1H), 7.74 (d, *J* = 1.8 Hz, 1H), 7.67 (d, *J* = 1.8 Hz, 1H), 4.14 (t, *J* = 7.2 Hz, 2H), 3.84 (s, 3H), 1.78 (p, *J* = 7.2 Hz, 2H), 1.25 (dh, *J* = 13.9, 6.7 Hz, 10H), 0.85 (t, *J* = 6.7 Hz, 3H). ^{13}C NMR (101 MHz, DMSO) δ = 136.53, 123.61, 122.27, 119.54 (q, *J* = 321.8 Hz), 48.84, 35.72, 31.18, 29.41, 28.49, 28.35, 25.51, 22.06, 13.85. ^{19}F NMR (376 MHz, DMSO) δ = -78.84.

[C_8C_1Im][NTf_2]$_{0.33}$Li[NTf_2]: ^{1}H NMR (400 MHz, DMSO) δ = 9.09 (s, 1H), 7.73 (d, *J* = 1.8 Hz, 1H), 7.67 (d, *J* = 1.8 Hz, 1H), 4.14 (t, *J* = 7.2 Hz, 2H), 3.84 (s, 2H), 1.78 (p, *J* = 7.1 Hz, 2H), 1.34 – 1.16 (m, 10H), 0.85 (t, *J* = 6.7 Hz, 3H). ^{13}C NMR (101 MHz, DMSO) δ = 136.55, 123.63, 122.28, 119.57 (q, *J* = 321.9 Hz), 48.87, 35.73, 31.20, 29.43, 28.51, 28.37, 25.53, 22.08, 13.85. ^{19}F NMR (376 MHz, DMSO) δ = -78.86.

[C_8C_1Im][NTf_2]$_{0.1}$Mg[NTf_2]$_2$: ^{1}H NMR (400 MHz, DMSO) δ = 9.08 (s, 1H), 7.74 (d, *J* = 1.8 Hz, 1H), 7.67 (d, *J* = 1.7 Hz, 1H), 4.14 (t, *J* = 7.2 Hz, 2H), 3.84 (s, 3H), 1.78 (p, *J* = 7.2 Hz, 2H), 1.25 (dh, *J* = 13.8, 6.8 Hz, 10H), 0.85 (t, *J* = 6.6 Hz, 3H). ^{13}C NMR (101 MHz, DMSO) δ = 136.54, 123.64, 122.29, 119.56 (q, *J* = 321.9 Hz), 48.87, 35.74, 31.20, 29.43, 28.51, 28.37, 25.54, 22.08, 13.87. ^{19}F NMR (376 MHz, DMSO) δ = -78.83.

$[C_8C_1Im][NTf_2]_{0.15}Mg[NTf_2]_2$: ^{1}H NMR (400 MHz, DMSO) δ = 9.09 (s, 1H), 7.74 (d, *J* = 1.8 Hz, 1H), 7.68 (d, *J* = 1.8 Hz, 1H), 4.14 (t, *J* = 7.2 Hz, 2H), 3.84 (s, 3H), 1.78 (p, *J* = 7.2 Hz, 2H), 1.25 (dh, *J* = 14.8, 6.9 Hz, 10H), 0.85 (t, *J* = 6.6 Hz, 3H). ^{13}C NMR (101 MHz, DMSO) δ = 136.52, 123.62, 122.28, 119.53 (q, *J* = 321.7 Hz), 48.83, 35.73, 31.18, 29.41, 28.49, 28.35, 25.51, 22.07, 13.88. ^{19}F NMR (376 MHz, DMSO) δ = -78.80.

$[C_8C_1Im][NTf_2]_{0.27}Mg[NTf_2]_2$: ^{1}H NMR (400 MHz, DMSO) δ = 9.09 (s, 1H), 7.75 (d, *J* = 1.7 Hz, 1H), 7.68 (d, *J* = 1.8 Hz, 1H), 4.14 (t, *J* = 7.2 Hz, 2H), 3.84 (s, 3H), 1.77 (p, *J* = 7.2 Hz, 2H), 1.25 (p, *J* = 9.6, 7.9 Hz, 11H), 0.85 (t, *J* = 6.6 Hz, 3H). ^{13}C NMR (101 MHz, DMSO) δ = 136.50, 123.62, 122.27, 119.51 (q, *J* = 321.9 Hz), 48.80, 35.74, 31.17, 29.39, 28.47, 28.33, 25.50, 22.06, 13.91. ^{19}F NMR (376 MHz, DMSO) δ = -78.74.

$[C_8C_1Im][NTf_2]_{0.33}Mg[NTf_2]_2$: ^{1}H NMR (400 MHz, DMSO) δ = 9.09 (s, 1H), 7.74 (t, *J* = 1.8 Hz, 1H), 7.67 (d, *J* = 1.8 Hz, 1H), 4.14 (t, *J* = 7.2 Hz, 2H), 3.84 (s, 3H), 1.77 (p, *J* = 7.2 Hz, 2H), 1.25 (m, 10H), 0.85 (t, *J* = 6.7 Hz, 3H). ^{13}C NMR (101 MHz, DMSO) δ = 136.54, 123.65, 122.30, 119.56 (q, *J* = 321.8 Hz), 48.85, 35.76, 31.20, 29.43, 28.51, 28.37, 25.54, 22.09, 13.91. ^{19}F NMR (376 MHz, DMSO) δ = -78.78.

$[C_8C_1Im][NTf_2]_{0.1}Co[NTf_2]_2$: ^{1}H NMR (400 MHz, DMSO) δ = 9.05 (s, 1H), 7.71 (t, *J* = 1.8 Hz, 1H), 7.64 (t, *J* = 1.9 Hz, 1H), 4.10 (t, *J* = 7.2 Hz, 2H), 3.80 (s, 3H), 1.83 – 1.61 (m, 2H), 1.30 – 1.12 (m, 10H), 0.81 (t, *J* = 6.7 Hz, 3H).

$[C_8C_1Im][NTf_2]_{0.15}Co[NTf_2]_2$: ^{1}H NMR (400 MHz, DMSO) δ = 9.00 (d, *J* = 1.7 Hz, 1H), 7.66 (t, *J* = 1.8 Hz, 1H), 7.60 (t, *J* = 1.8 Hz, 1H), 4.06 (t, *J* = 7.2 Hz, 2H), 3.76 (s, 3H), 1.78 – 1.59 (m, 2H), 1.24 – 1.10 (m, 10H), 0.83 – 0.70 (m, 3H). ^{13}C NMR (101 MHz, DMSO) δ = 134.28, 121.39, 120.04, 117.28 (q, *J* = 321.8 Hz), 46.58, 33.51, 28.94, 27.16, 26.25, 26.11, 23.27, 19.83, 11.68. ^{19}F NMR (376 MHz, DMSO) δ = -78.80.

$[C_8C_1Im][NTf_2]_{0.27}Co[NTf_2]_2$: ^{1}H NMR (400 MHz, DMSO) δ = 8.89 (s, 1H), 7.55 (d, *J* = 2.0 Hz, 1H), 7.48 (d, *J* = 2.0 Hz, 1H), 3.94 (t, *J* = 7.2 Hz, 2H), 3.64 (s, 1H), 1.58 (p, *J* = 7.1 Hz, 2H), 1.05 (d, *J* = 7.7 Hz, 10H), 0.69 – 0.60 (m, 3H). ^{13}C NMR (101 MHz, DMSO) δ = 131.35, 118.46, 117.11, 114.36 (q, *J* = 321.8 Hz), 43.67, 30.59, 26.02, 24.24, 23.32, 23.18, 20.35, 16.91, 8.75. ^{19}F NMR (376 MHz, DMSO) δ = -78.88.

$[C_8C_1Im][NTf_2]_{0.33}Co[NTf_2]_2$: ^{1}H NMR (400 MHz, DMSO) δ = 8.80 (s, 1H), 7.46 (s, 1H), 7.39 (s, 1H), 3.86 (t, *J* = 7.3 Hz, 2H), 3.56 (s, 3H), 1.48 (q, *J* = 7.1 Hz, 2H), 0.96 (d, *J* = 8.5 Hz, 10H), 0.56 (t, *J* = 6.5 Hz, 3H). ^{13}C NMR (101 MHz, DMSO) δ = 129.31, 116.42, 115.07, 112.31 (q, *J* = 322.0 Hz), 41.63, 28.55, 23.97, 22.20, 21.28, 21.14, 18.30, 14.86, 6.70. ^{19}F NMR (376 MHz, DMSO) δ = -78.94.

$[C_8C_1Im][NTf_2]_{0.1}Zn[NTf_2]_2$: ^{1}H NMR (400 MHz, DMSO) δ = 9.10 (s, 1H), 7.76 (s, 1H), 7.69 (s, 1H), 4.15 (t, *J* = 7.2 Hz, 2H), 3.85 (s, 3H), 1.96 – 1.66 (m, 2H), 1.42 – 1.12 (m, 11H), 0.86 (d, *J* = 7.2 Hz, 3H). ^{13}C NMR (101 MHz, DMSO) δ = 135.41, 122.53, 121.18, 118.41 (q, *J* = 321.9 Hz), 47.71, 34.65, 30.07, 28.30, 27.38, 27.24, 24.41, 20.97, 12.82. ^{19}F NMR (376 MHz, DMSO) δ = -78.75. ^{19}F NMR (376 MHz, DMSO) δ = -78.76.

$[C_8C_1Im][NTf_2]_{0.15}Zn[NTf_2]_2$: ^{1}H NMR (400 MHz, DMSO) δ = 9.09 (s, 1H), 7.75 (t, *J* = 1.8 Hz, 1H), 7.69 (t, *J* = 1.8 Hz, 1H), 4.14 (t, *J* = 7.2 Hz, 2H), 3.84 (s, 3H), 1.89 – 1.67 (m, 2H), 1.25 (d, *J* = 6.4 Hz, 9H), 0.86 (d, *J* = 7.1 Hz, 2H). ^{13}C NMR (101 MHz, DMSO) δ = 136.49, 123.61, 122.26, 119.50 (q, *J* = 321.9 Hz), 48.79, 35.74, 31.16, 29.38, 28.47, 28.33, 25.49, 22.05, 13.91. ^{19}F NMR (376 MHz, DMSO) δ = -78.74.

$[C_8C_1Im][NTf_2]_{0.27}Zn[NTf_2]_2$: ^{1}H NMR (400 MHz, DMSO) δ = 9.09 (s, 1H), 7.75 (t, *J* = 1.8 Hz, 1H), 7.68 (t, *J* = 1.8 Hz, 1H), 4.14 (t, *J* = 7.2 Hz, 2H), 3.84 (s, 3H), 1.77 (p, *J* = 7.3 Hz, 2H), 1.25 (q, *J* = 11.9, 8.9 Hz, 10H), 0.92 – 0.76 (m, 3H). ^{13}C NMR (101 MHz, DMSO) δ = 136.50, 123.62, 122.28, 119.51 (q, *J* = 321.9

Hz), 48.80, 35.74, 31.17, 29.39, 28.48, 28.34, 25.50, 22.06, 13.91. ^{19}F NMR (376 MHz, DMSO) δ = -78.74.

[C$_8$C$_1$Im][NTf$_2$]$_{0.33}$Zn[NTf$_2$]$_2$: ^{1}H NMR (400 MHz, DMSO) δ = 9.08 (s, 1H), 7.74 (d, *J* = 1.8 Hz, 1H), 7.67 (d, *J* = 1.8 Hz, 1H), 4.14 (t, *J* = 7.2 Hz, 2H), 3.84 (s, 3H), 1.77 (p, *J* = 7.2 Hz, 2H), 1.26 (m, 10H), 0.85 (t, *J* = 6.7 Hz, 3H). ^{13}C NMR (101 MHz, DMSO) δ = 136.57, 123.66, 122.32, 119.58 (q, *J* = 321.8 Hz), 48.88, 35.77, 31.22, 29.45, 28.53, 28.39, 25.55, 22.11, 13.92. ^{19}F NMR (376 MHz, DMSO) δ = -78.80.

[C$_8$C$_1$Im][NTf$_2$]$_{0.33}$Ni[NTf$_2$]$_2$: ^{1}H NMR (400 MHz, DMSO) δ = 9.06 (s, 1H), 7.71 (s, 1H), 7.64 (s, 1H), 4.12 (t, *J* = 7.1 Hz, 2H), 3.82 (s, 2H), 1.81 – 1.67 (m, 2H), 1.33 – 1.08 (m, 9H), 0.82 (d, *J* = 6.9 Hz, 3H). ^{13}C NMR (101 MHz, DMSO) δ = 136.40, 123.51, 122.16, 119.44 (q, *J* = 322.1 Hz), 48.73, 35.63, 31.07, 29.29, 28.38, 28.24, 25.40, 21.96, 13.78. ^{19}F NMR (376 MHz, DMSO) δ = -78.92.

2. Data

NMR Spectra

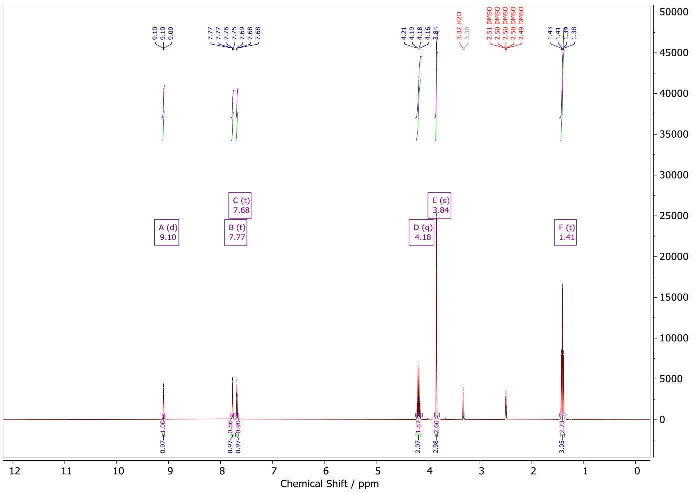

Figure S1 ^{1}H NMR of [C$_2$C$_1$Im][NTf$_2$] in DMSO-*d$_6$*.

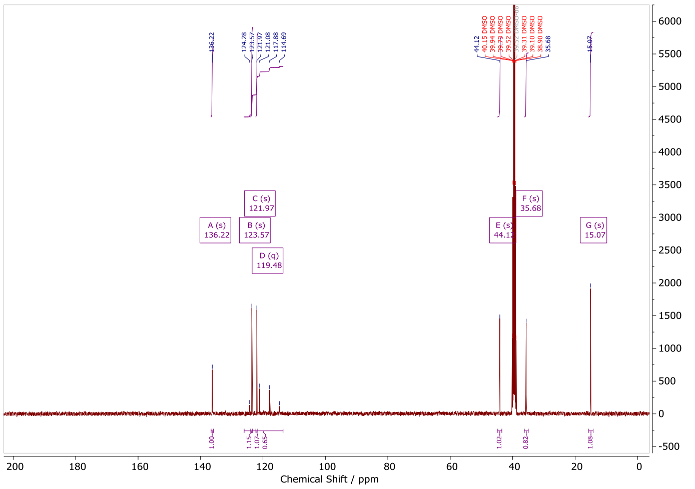

Figure S2 ^{13}C NMR of [C$_2$C$_1$Im][NTf$_2$] in DMSO-*d$_6$*.

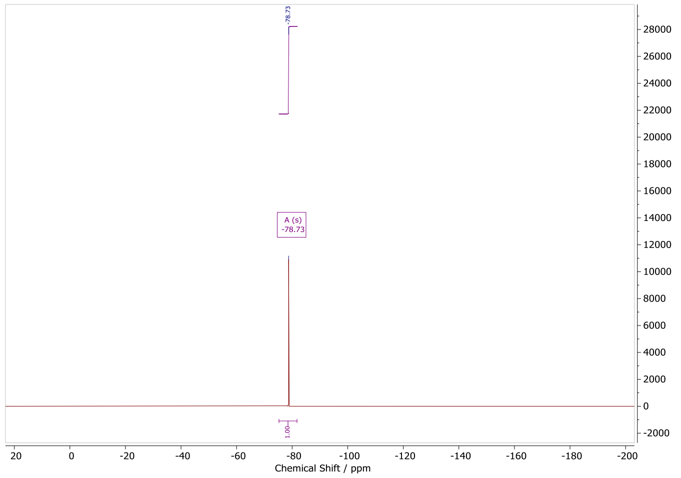

Figure S3 ^{19}F NMR of $[C_2C_1Im][NTf_2]$ in DMSO-d_6.

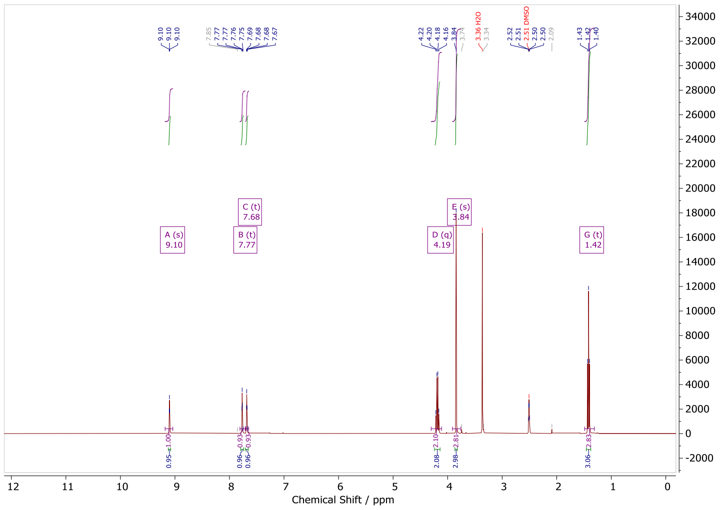

Figure S4 ^{1}H NMR of $[C_2C_1Im][NTf_2]_{0.33}Zn[NTf_2]_2$ in DMSO-d_6.

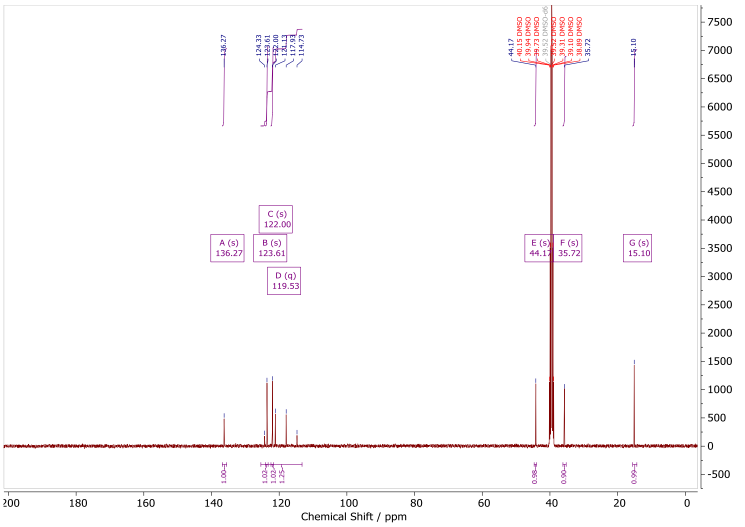

Figure S5 ^{13}C NMR of $[C_2C_1Im][NTf_2]_{0.33}Zn[NTf_2]_2$ in DMSO-d_6.

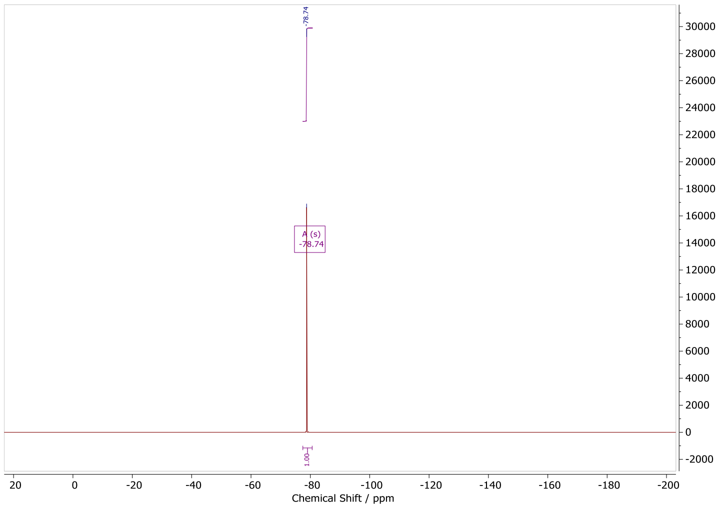

Figure S6 ^{19}F NMR of $[C_2C_1Im][NTf_2]_{0.33}Zn[NTf_2]_2$ in DMSO-d_6.

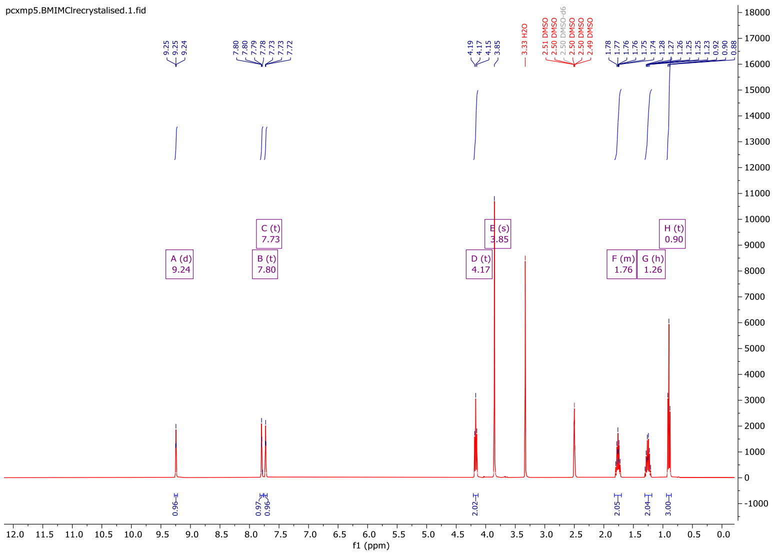

Figure S7 ^{1}H NMR of $[C_4C_1Im]Cl$ in DMSO-d_6.

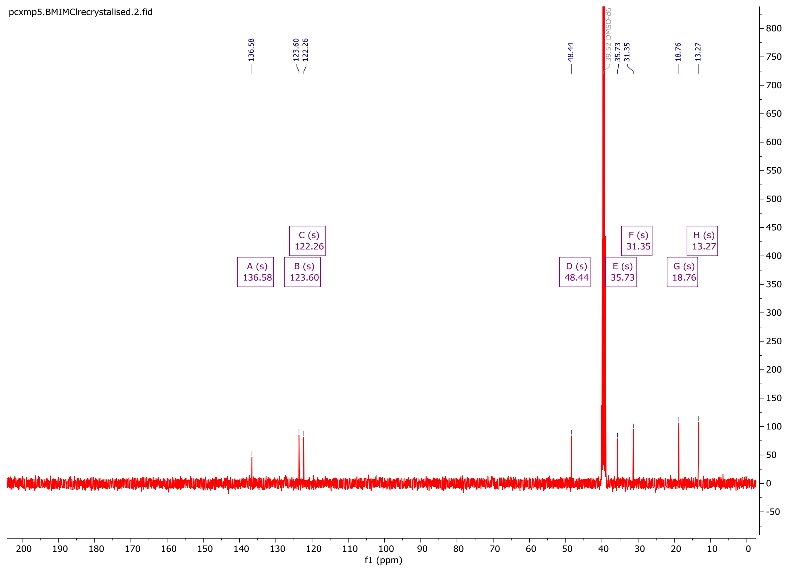

Figure S8 ^{13}C NMR of $[C_4C_1Im]Cl$ in DMSO-d_6.

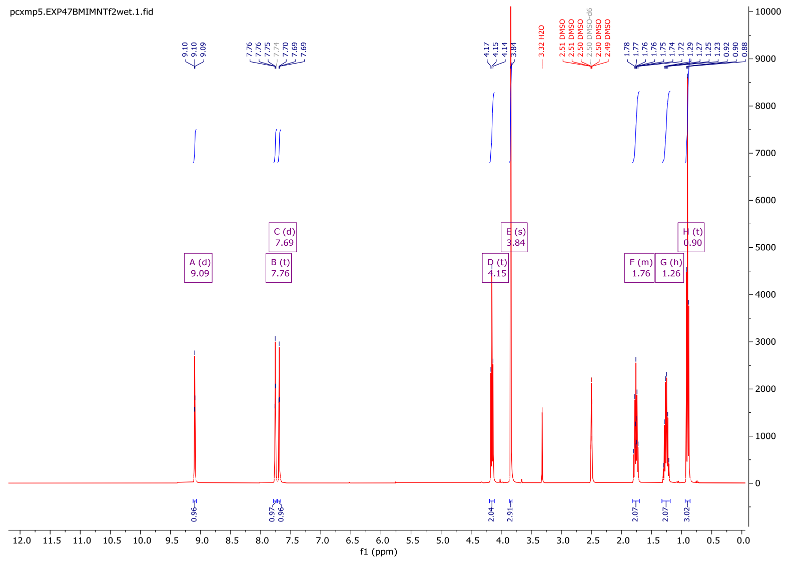

Figure S9 ^{1}H NMR of $[C_4C_1Im][NTf_2]$ in DMSO-d_6.

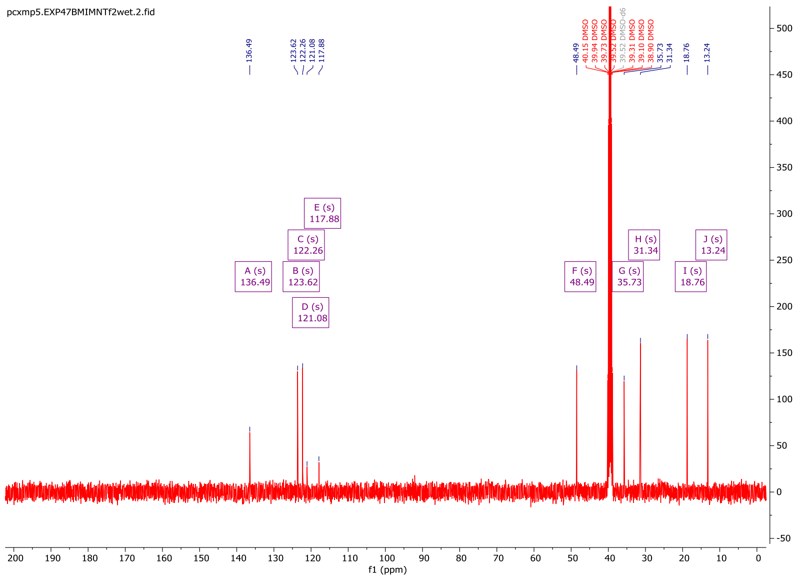

Figure S10 ^{13}C NMR of $[C_4C_1Im][NTf_2]$ in DMSO-d_6.

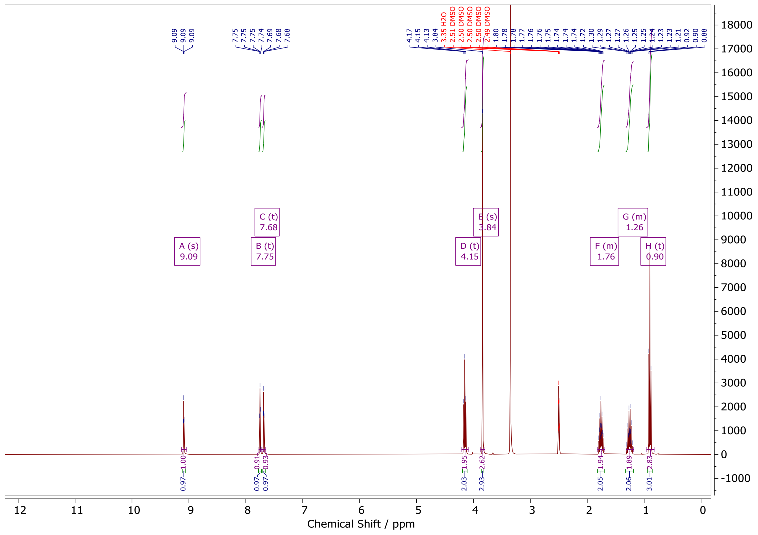

Figure S11 ^{1}H NMR of $[C_4C_1Im][NTf_2]_{0.33}Zn[NTf_2]_2$ in DMSO-d_6.

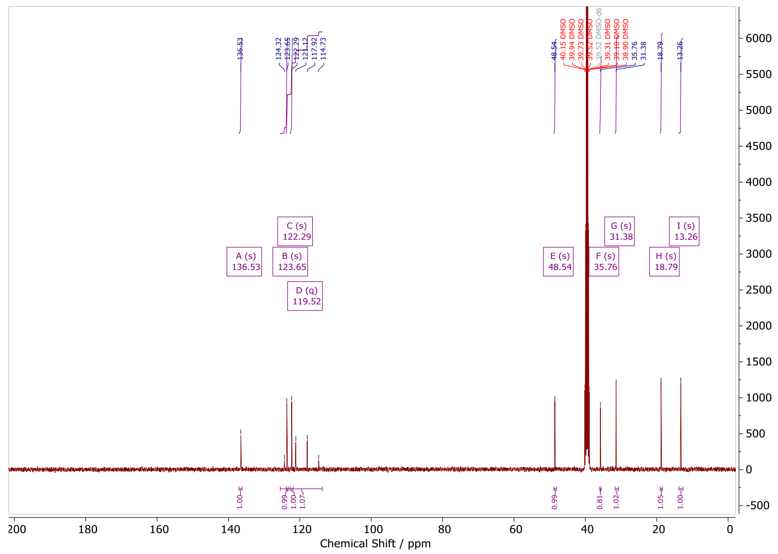

Figure S12 ^{13}C NMR of $[C_4C_1Im][NTf_2]_{0.33}Zn[NTf_2]_2$ in DMSO-d_6.

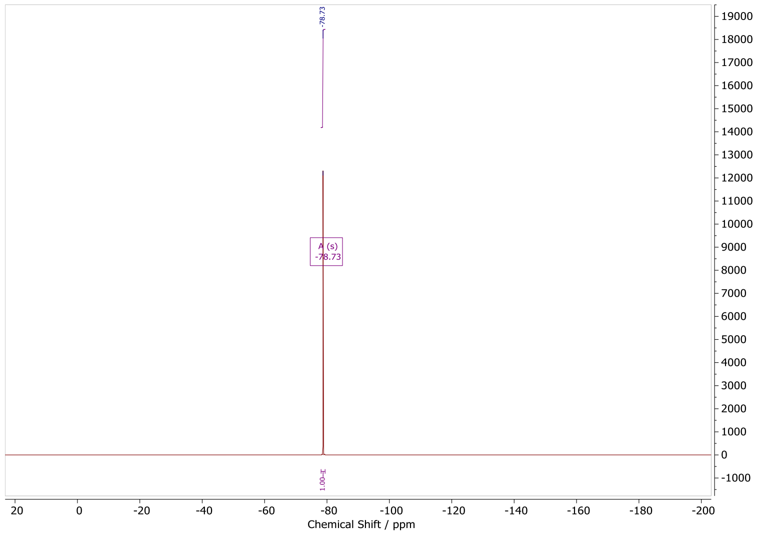

Figure S13 ^{19}F NMR of $[C_4C_1Im][NTf_2]_{0.33}Zn[NTf_2]_2$ in DMSO-d_6.

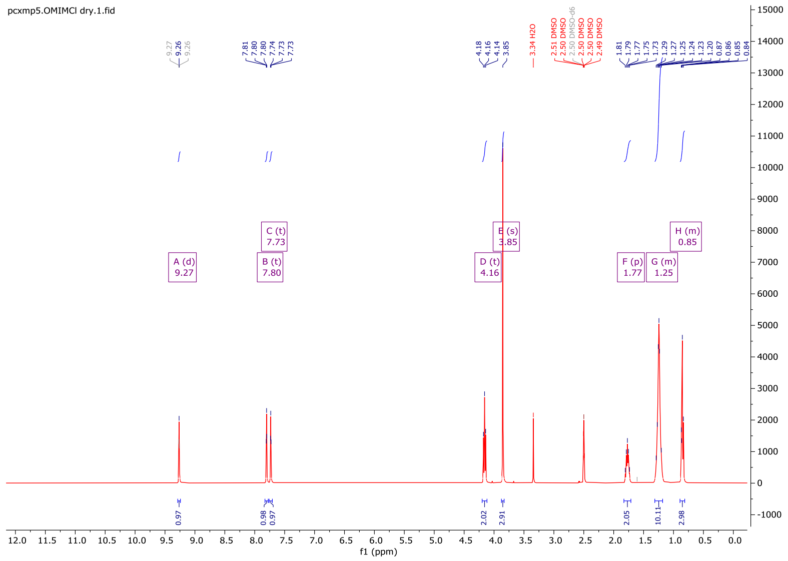

Figure S14 ^{1}H NMR of $[C_8C_1Im]Cl$ in DMSO-d_6.

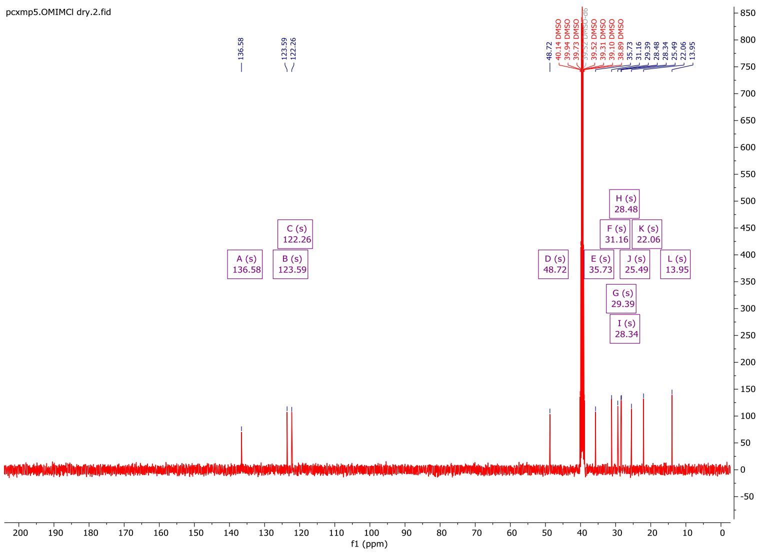

Figure S15 ^{13}C NMR of $[C_8C_1Im]Cl$ in DMSO-d_6.

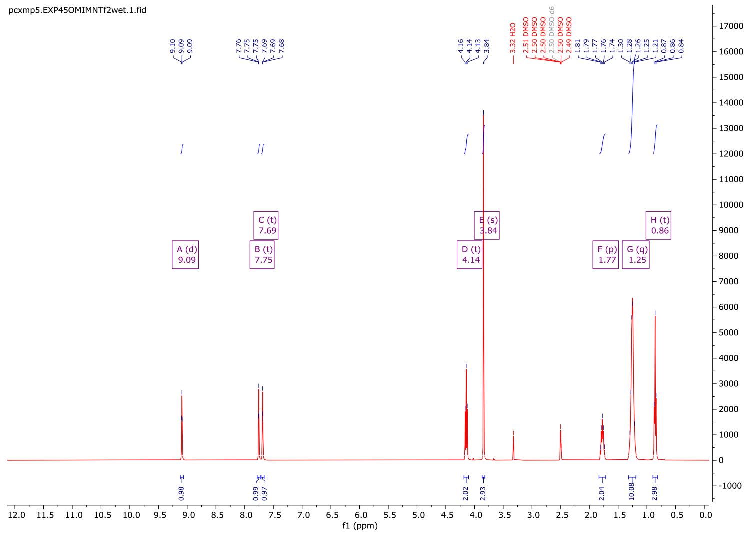

Figure S16 ^{1}H NMR of $[C_8C_1Im][NTf_2]$ in DMSO-d_6.

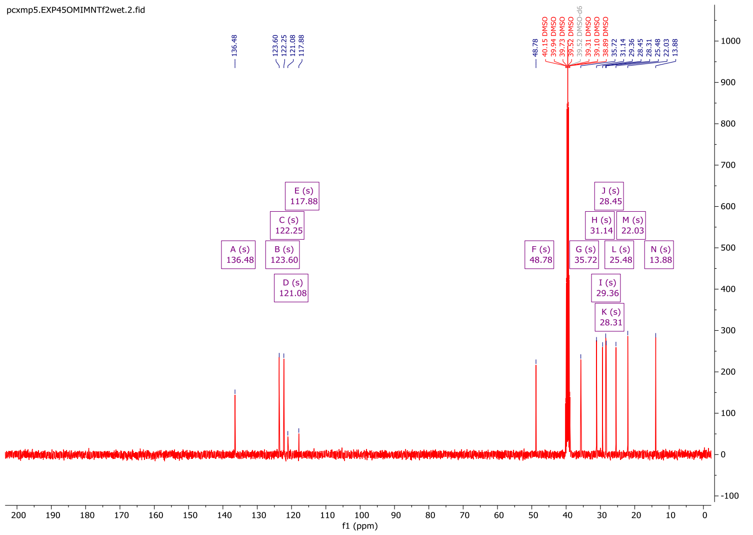

Figure S17 ^{13}C NMR of $[C_8C_1Im][NTf_2]$ in DMSO-d_6.

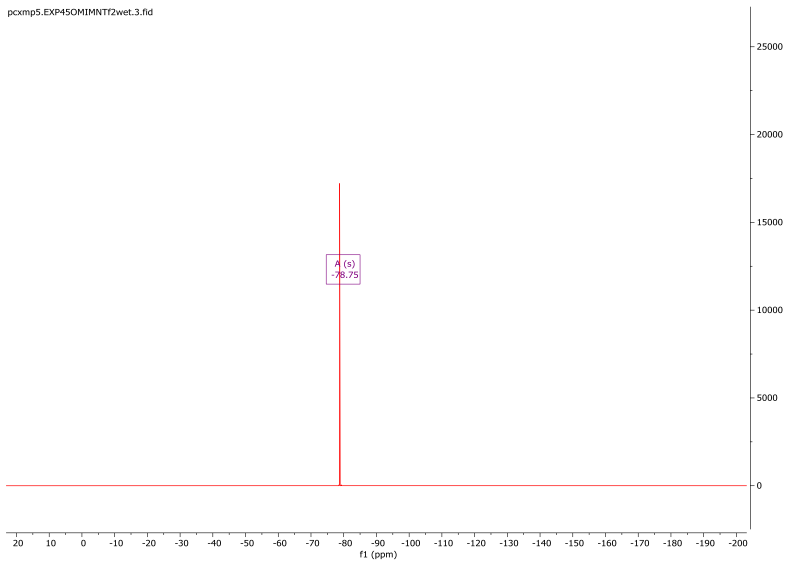

Figure S18 ^{19}F NMR of $[C_8C_1Im][NTf_2]$ in DMSO-d_6.

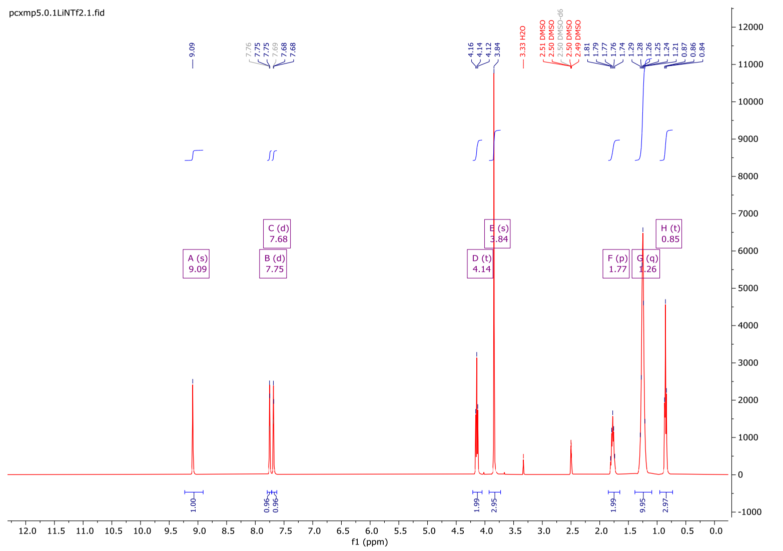

Figure S19 ^{1}H NMR of $[C_8C_1Im][NTf_2]_{0.1}Li[NTf_2]$ in DMSO-d_6.

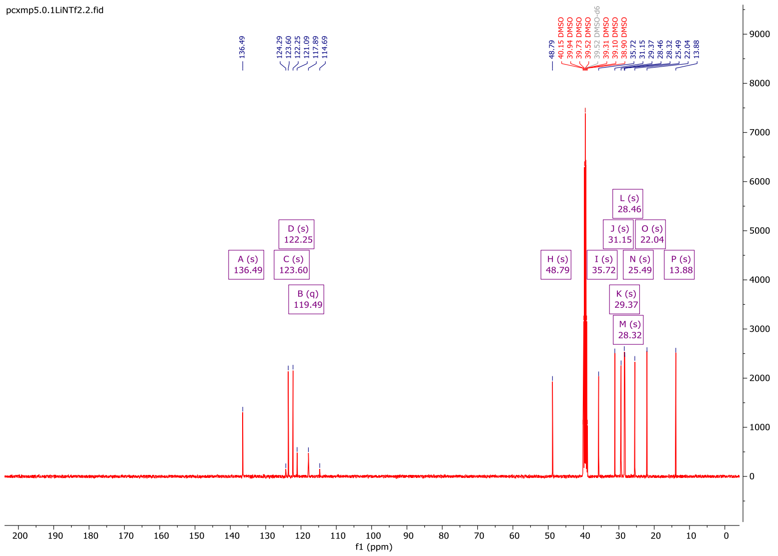

Figure S20 ^{13}C NMR of $[C_8C_1Im][NTf_2]_{0.1}Li[NTf_2]$ in DMSO-d_6.

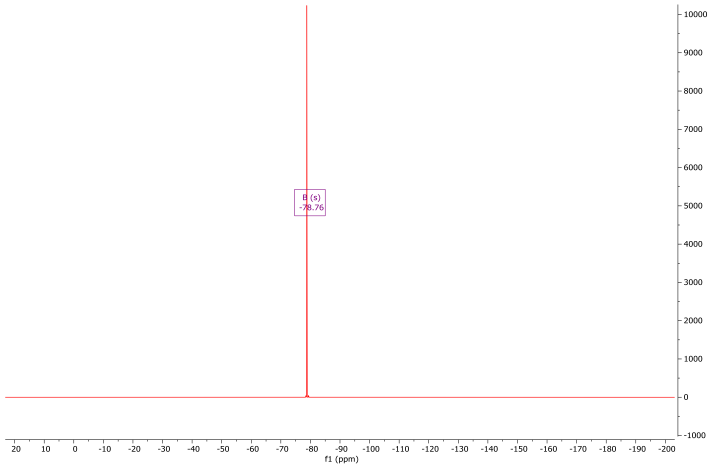

Figure S21 ^{19}F NMR of $[C_8C_1Im][NTf_2]_{0.1}Li[NTf_2]$ in DMSO-d_6.

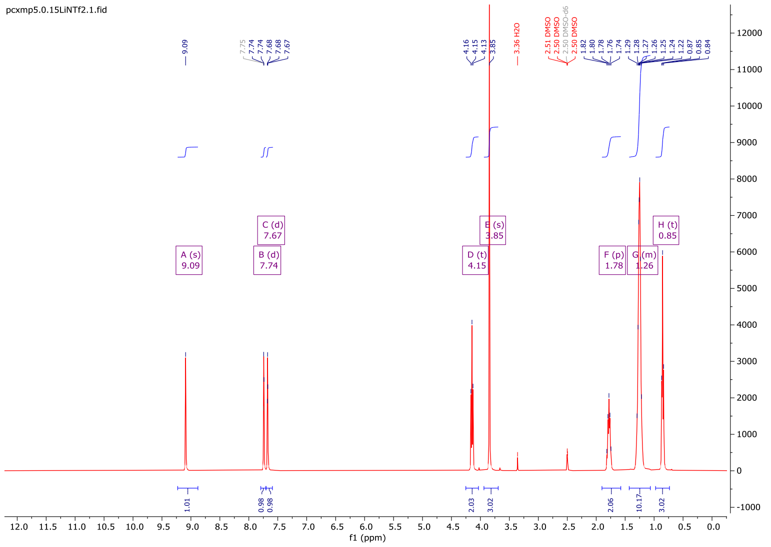

Figure S22 ^{1}H NMR of $[C_8C_1Im][NTf_2]_{0.15}Li[NTf_2]$ in DMSO-d_6.

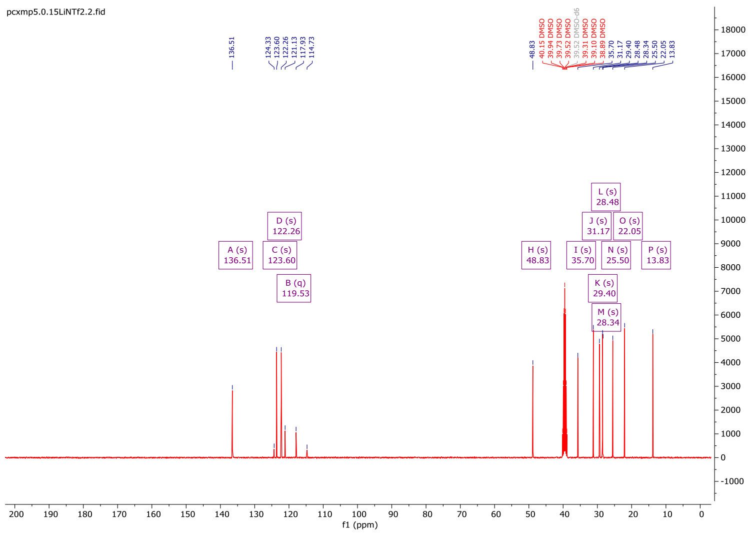

Figure S23 ^{13}C NMR of $[C_8C_1Im][NTf_2]_{0.15}Li[NTf_2]$ in DMSO-d_6.

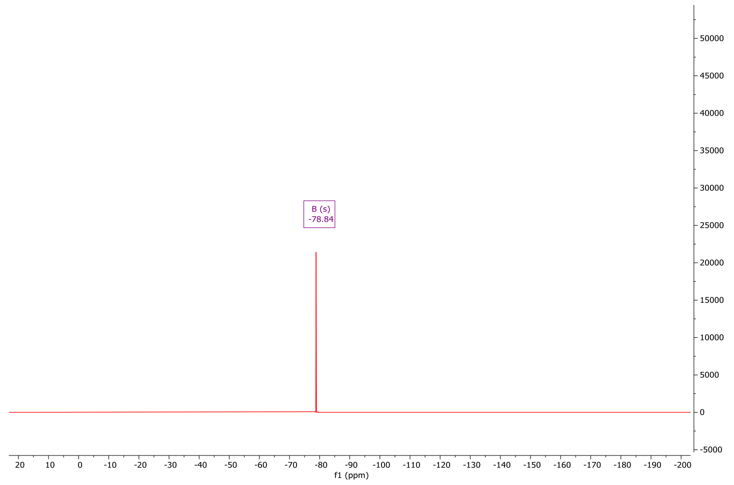

Figure S24 ^{13}C NMR of $[C_8C_1Im][NTf_2]_{0.15}Li[NTf_2]$ in DMSO-d_6.

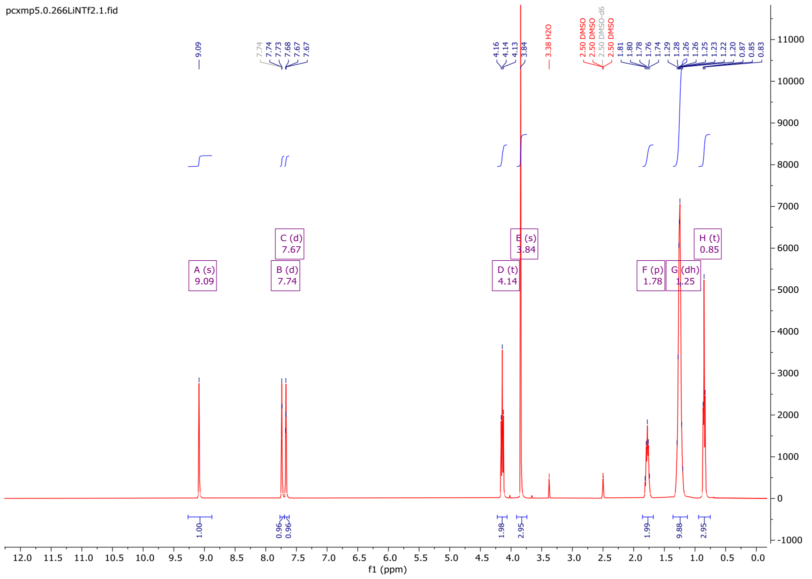

Figure S25 ^{1}H NMR of $[C_8C_1Im][NTf_2]_{0.27}Li[NTf_2]$ in DMSO-d_6.

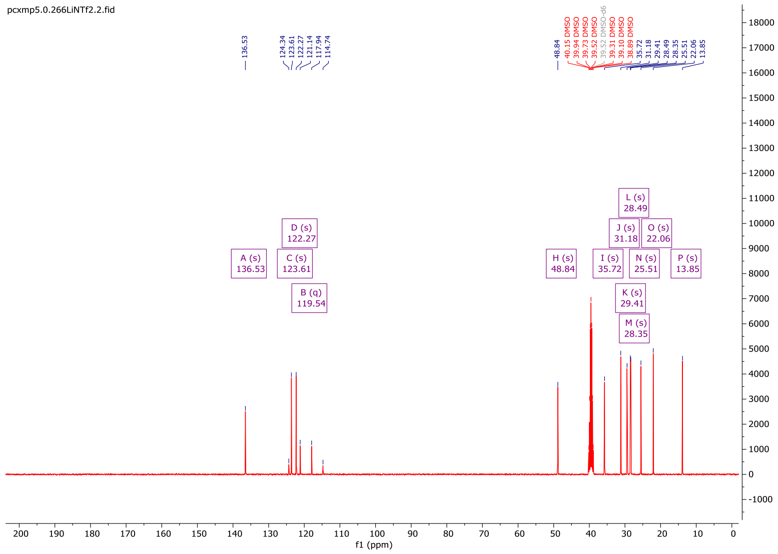

Figure S26 ^{13}C NMR of $[C_8C_1Im][NTf_2]_{0.27}Li[NTf_2]$ in DMSO-d_6.

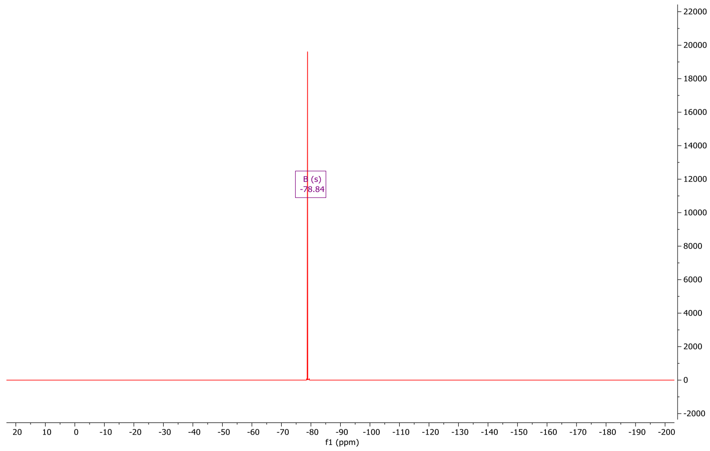

Figure S27 ^{19}F NMR of $[C_8C_1Im][NTf_2]_{0.27}Li[NTf_2]$ in DMSO-d_6.

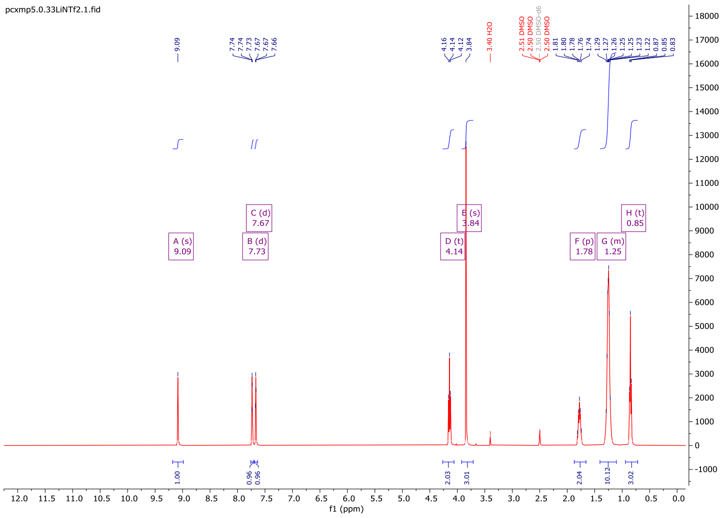

Figure S28 ^{1}H NMR of $[C_8C_1Im][NTf_2]_{0.33}Li[NTf_2]$ in DMSO-d_6.

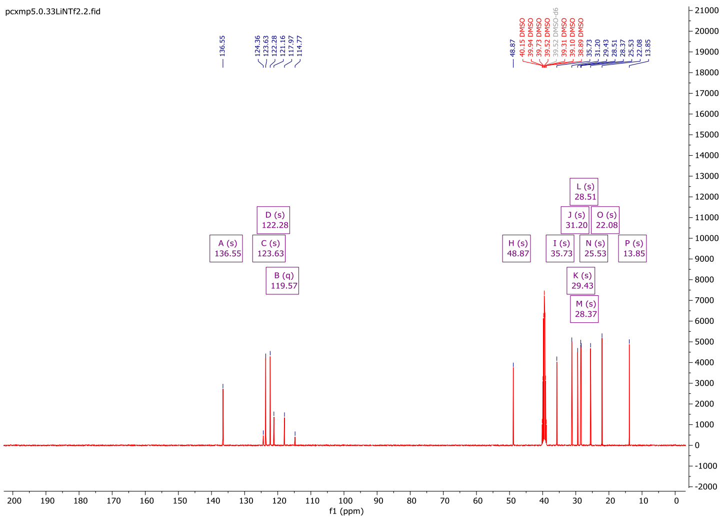

Figure S29 ^{13}C NMR of $[C_8C_1Im][NTf_2]_{0.33}Li[NTf_2]$ in DMSO-d_6.

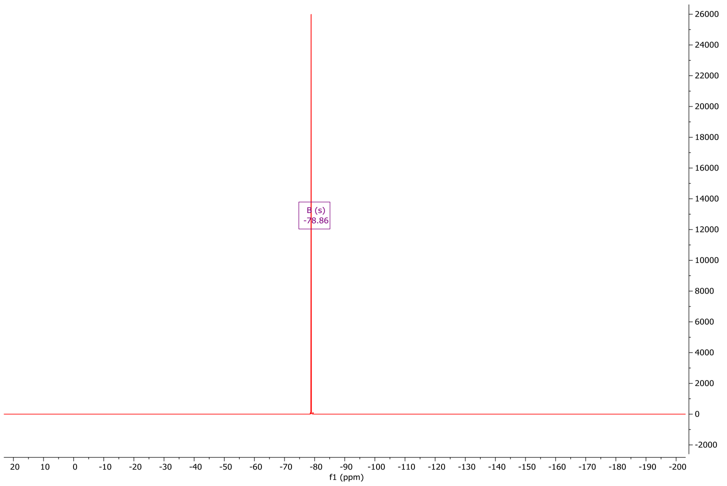

Figure S30 ^{19}F NMR of $[C_8C_1Im][NTf_2]_{0.33}Li[NTf_2]$ in DMSO-d_6.

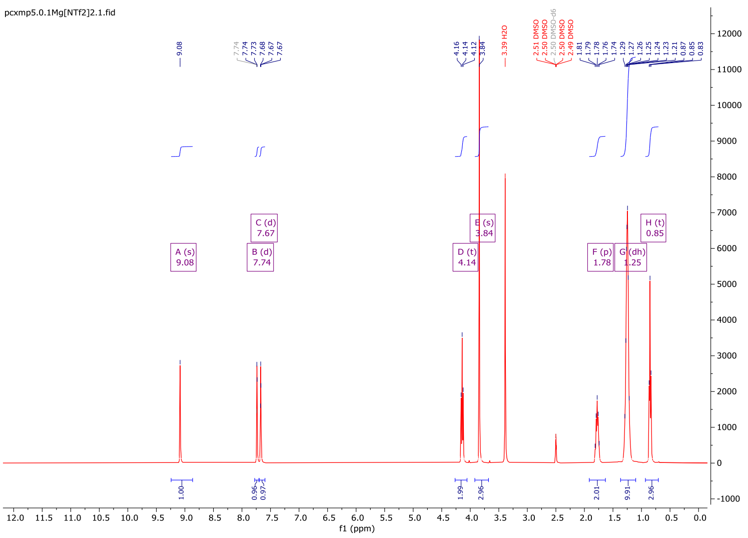

Figure S31 ^{1}H NMR of $[C_8C_1Im][NTf_2]_{0.1}Mg[NTf_2]_2$ in DMSO-d_6.

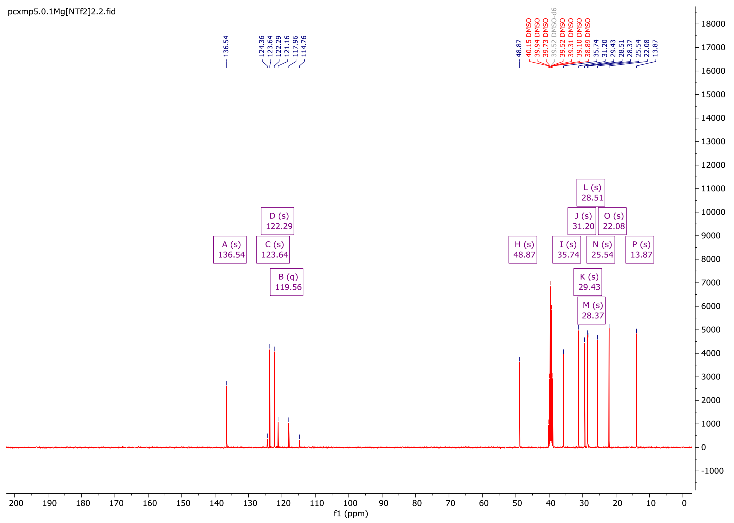

Figure S32 ^{13}C NMR of $[C_8C_1Im][NTf_2]_{0.1}Mg[NTf_2]_2$ in DMSO-d_6.

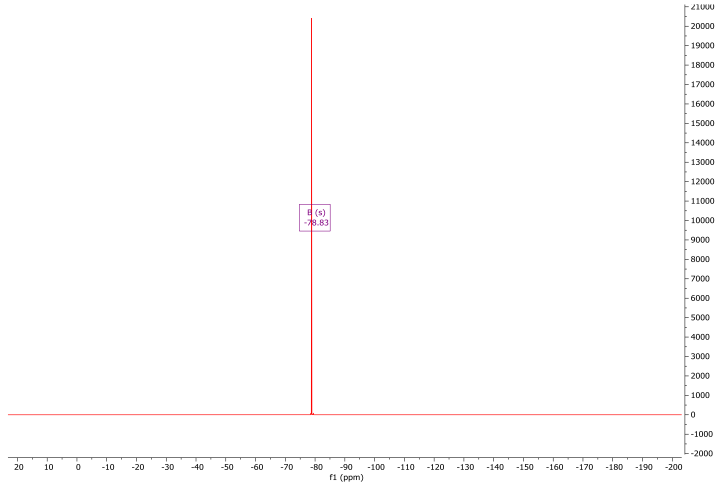

Figure S33 ^{19}F NMR of $[C_8C_1Im][NTf_2]_{0.1}Mg[NTf_2]_2$ in DMSO-d_6.

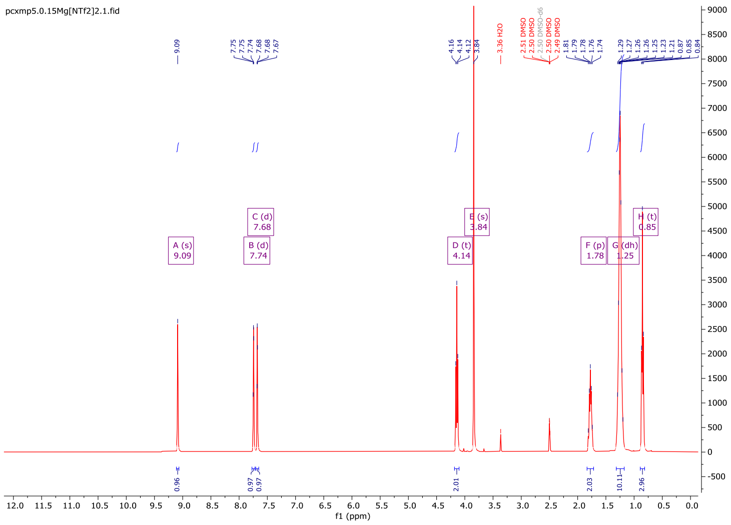

Figure S34 ^{1}H NMR of $[C_8C_1Im][NTf_2]_{0.15}Mg[NTf_2]_2$ in DMSO-d_6.

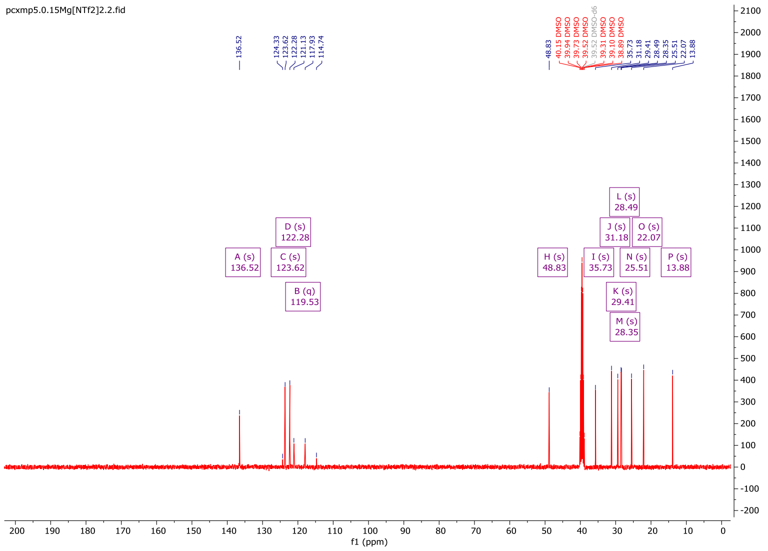

Figure S35 ^{13}C NMR of $[C_8C_1Im][NTf_2]_{0.15}Mg[NTf_2]_2$ in DMSO-d_6.

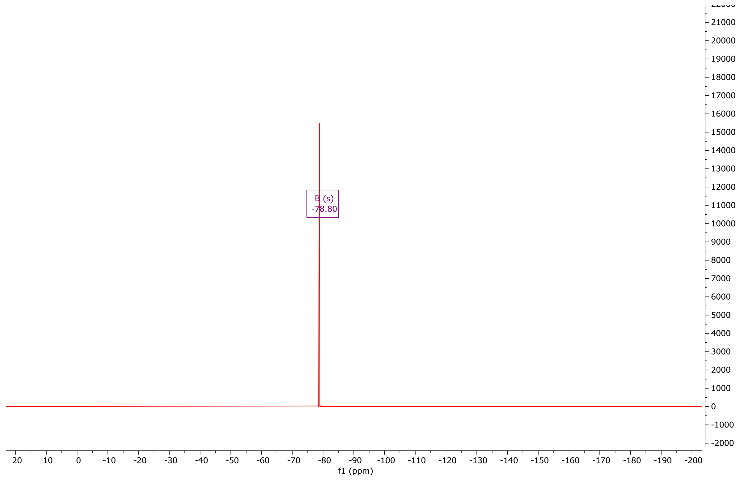

Figure S36 ^{19}F NMR of $[C_8C_1Im][NTf_2]_{0.15}Mg[NTf_2]_2$ in DMSO-d_6.

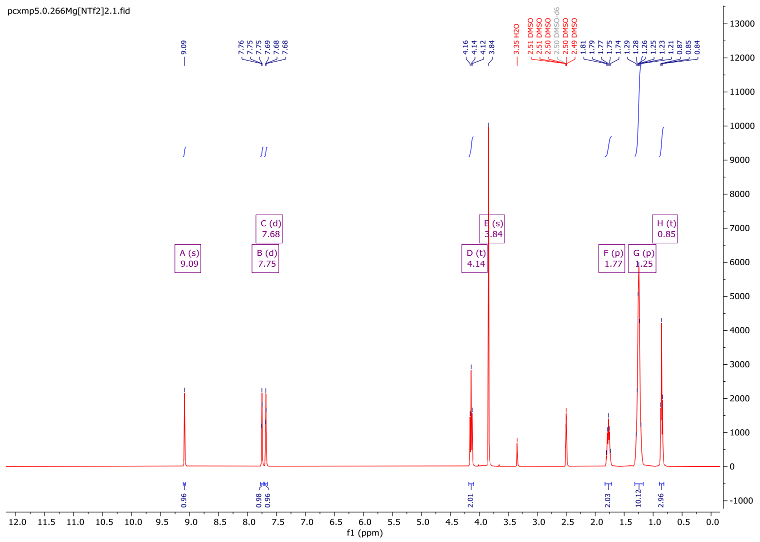

Figure S37 ^{1}H NMR of $[C_8C_1Im][NTf_2]_{0.27}Mg[NTf_2]_2$ in DMSO-d_6.

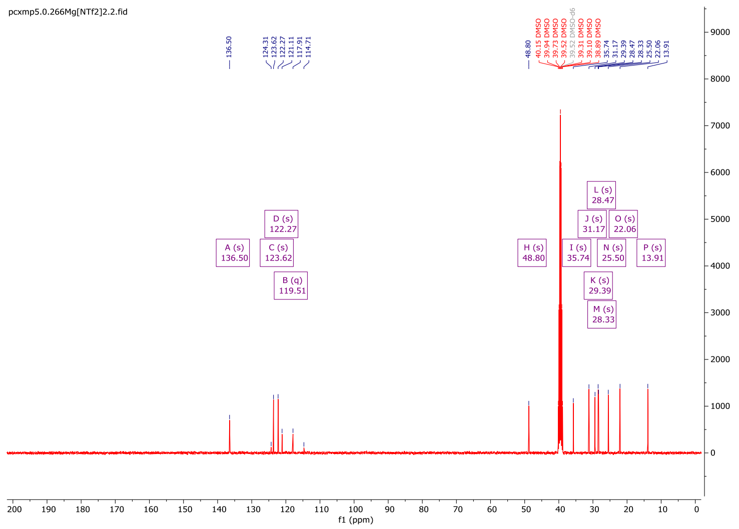

Figure S38 ^{13}C NMR of $[C_8C_1Im][NTf_2]_{0.27}Mg[NTf_2]_2$ in DMSO-d_6.

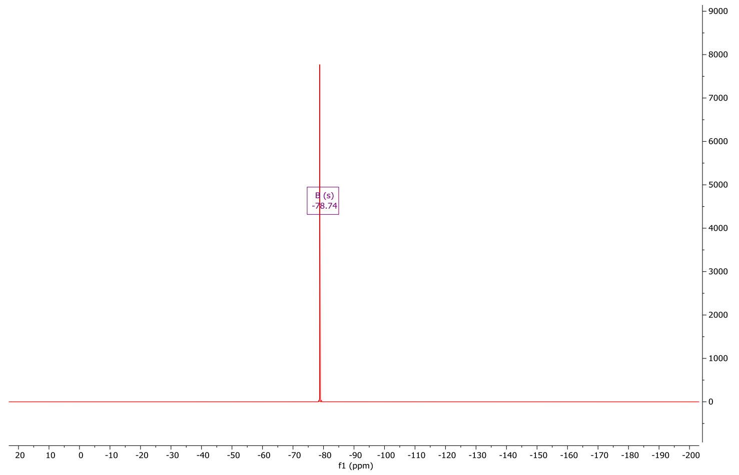

Figure S39 ^{19}F NMR of $[C_8C_1Im][NTf_2]_{0.27}Mg[NTf_2]_2$ in DMSO-d_6.

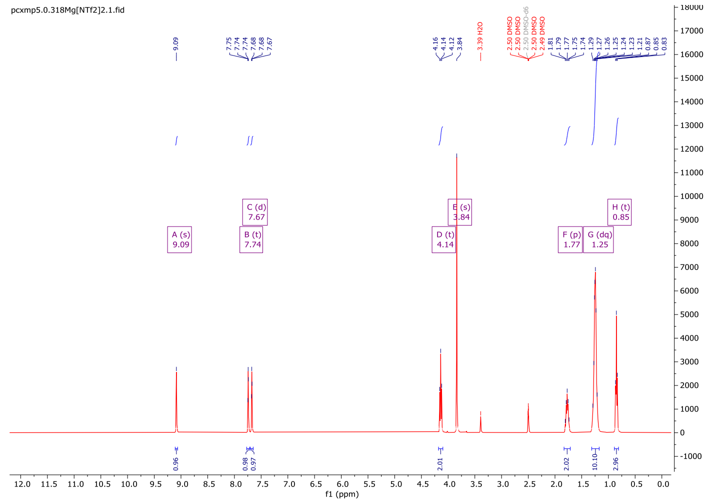

Figure S40 ^{1}H NMR of $[C_8C_1Im][NTf_2]_{0.32}Mg[NTf_2]_2$ in DMSO-d_6.

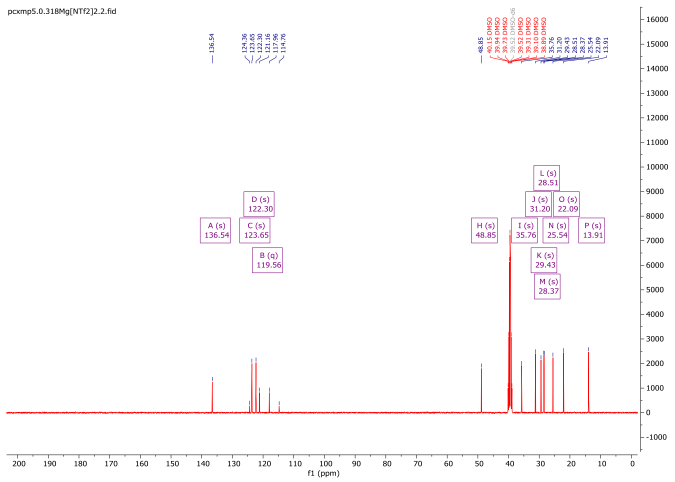

Figure S41 ^{13}C NMR of $[C_8C_1Im][NTf_2]_{0.32}Mg[NTf_2]_2$ in DMSO-d_6.

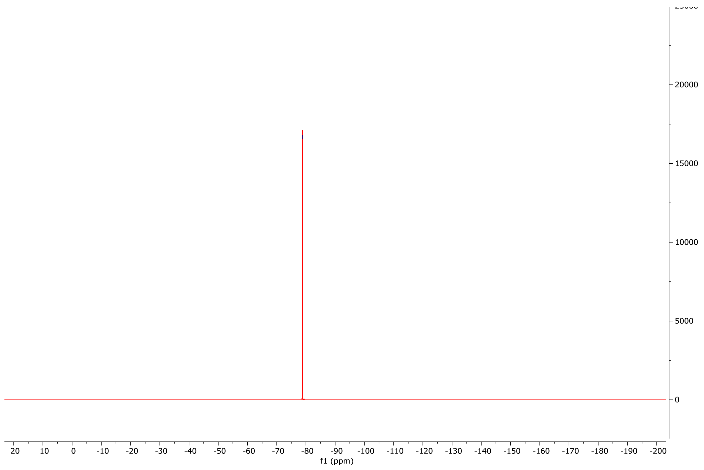

Figure S42 ^{19}F NMR of $[C_8C_1Im][NTf_2]_{0.32}Mg[NTf_2]_2$ in DMSO-d_6.

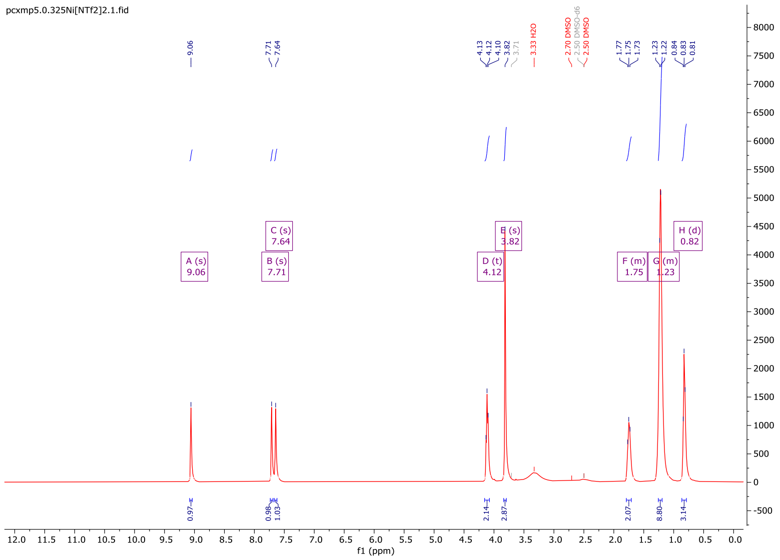

Figure S43 ^{1}H NMR of $[C_8C_1Im][NTf_2]_{0.33}Ni[NTf_2]_2$ in DMSO-d_6.

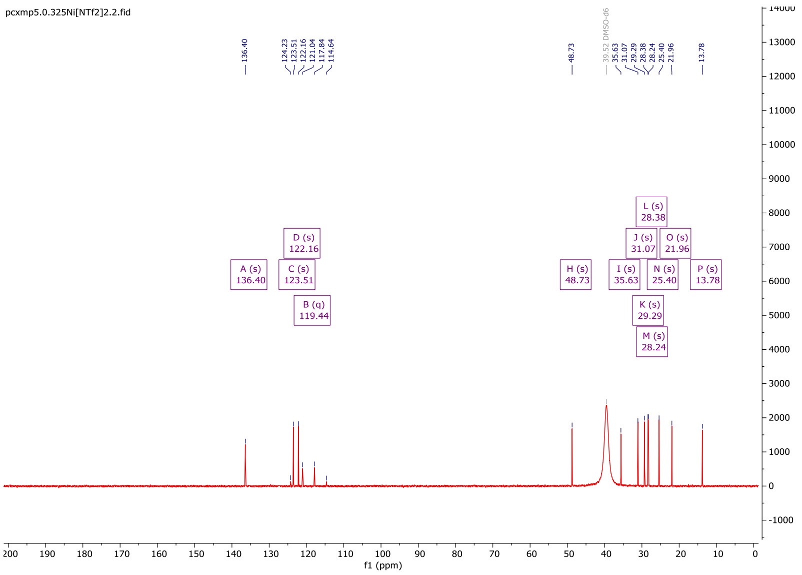

Figure S44 ^{13}C NMR of $[C_8C_1Im][NTf_2]_{0.33}Ni[NTf_2]_2$ in DMSO-d_6.

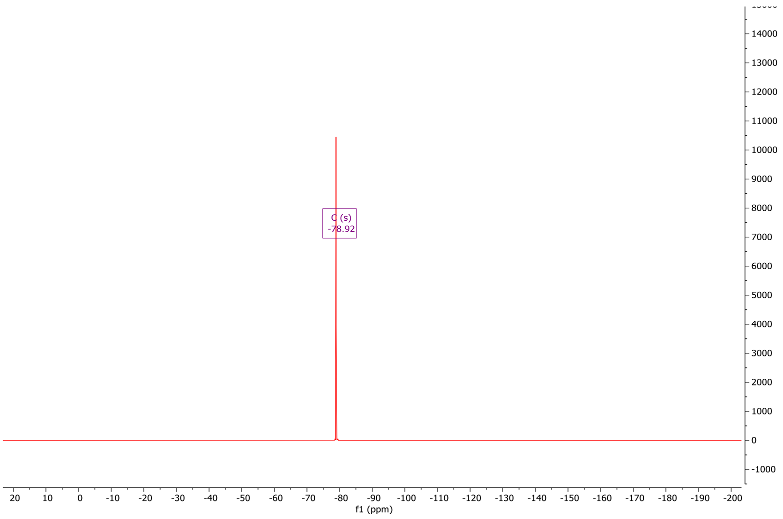

Figure S45 ^{19}F NMR of $[C_8C_1Im][NTf_2]_{0.33}Ni[NTf_2]_2$ in DMSO-d_6.

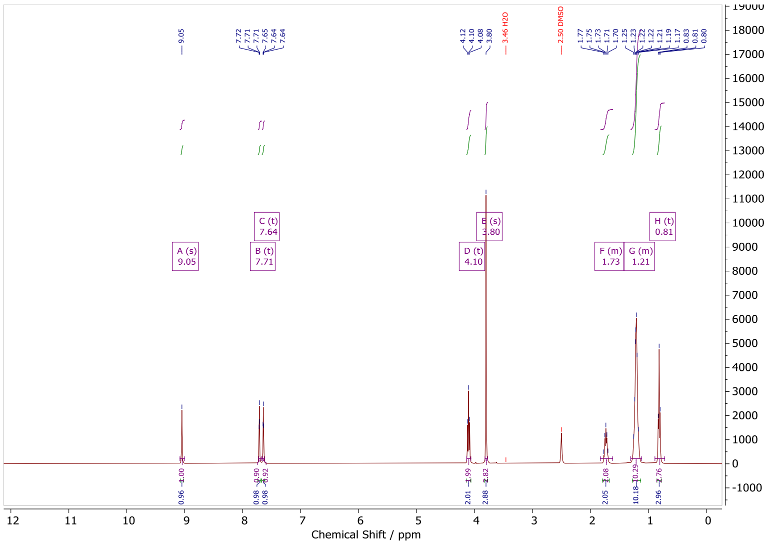

Figure S46 ^{1}H NMR of $[C_8C_1Im][NTf_2]_{0.1}Co[NTf_2]_2$ in DMSO-d_6.

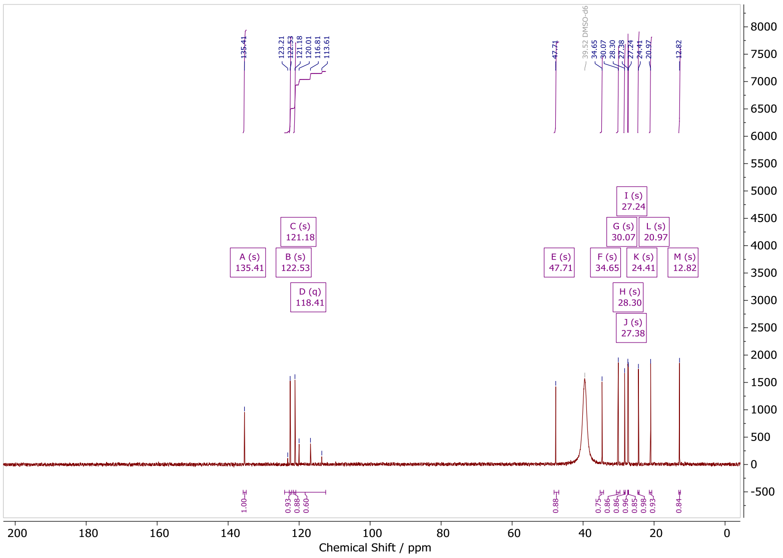

Figure S47 ^{13}C NMR of $[C_8C_1Im][NTf_2]_{0.1}Co[NTf_2]_2$ in DMSO-d_6.

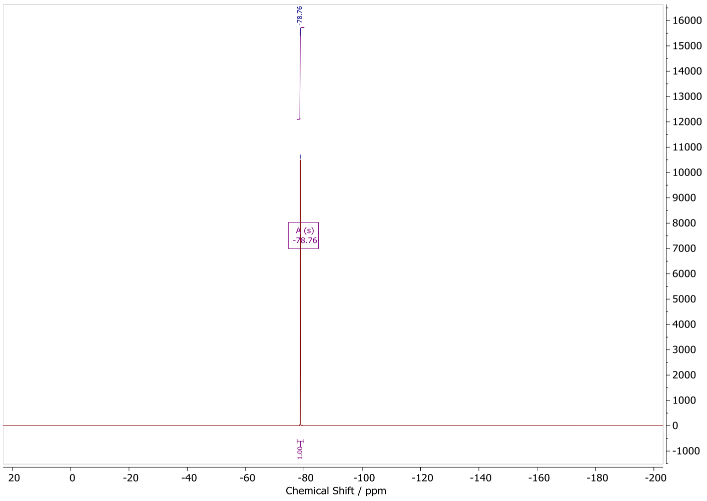

Figure S48 ^{19}F NMR of $[C_8C_1Im][NTf_2]_{0.1}Co[NTf_2]_2$ in DMSO-d_6.

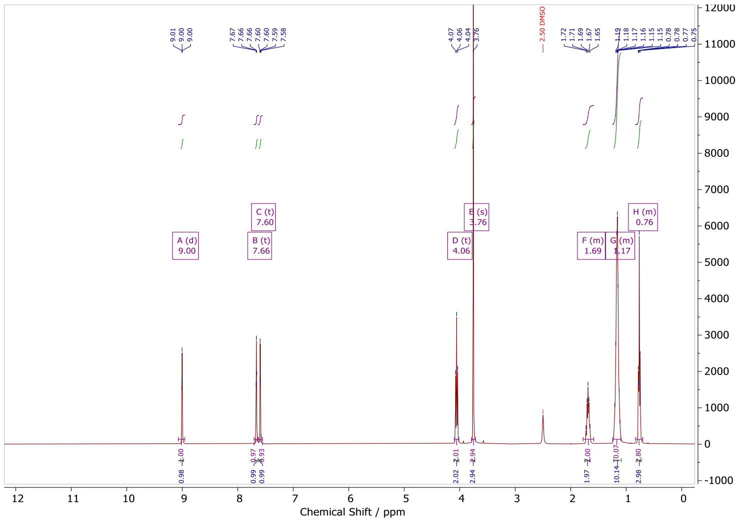

Figure S49 ^{1}H NMR of $[C_8C_1Im][NTf_2]_{0.15}Co[NTf_2]_2$ in DMSO-d_6.

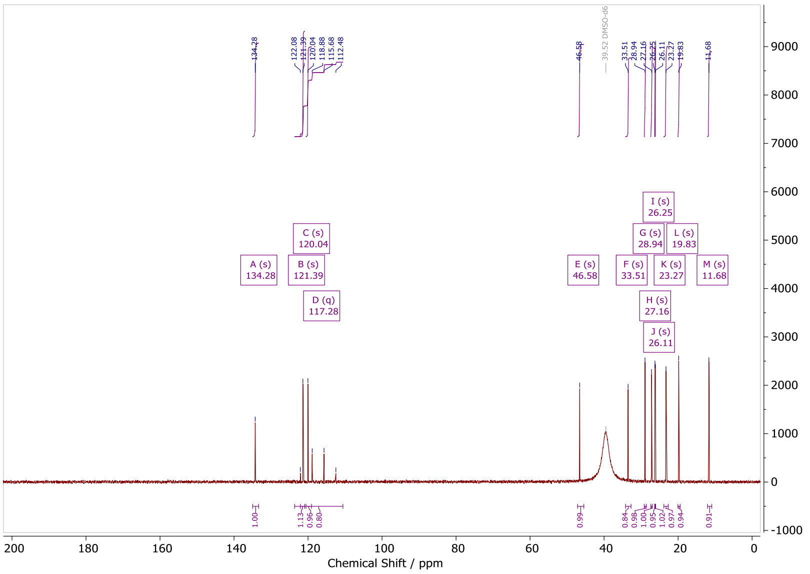

Figure S50 ^{13}C NMR of $[C_8C_1Im][NTf_2]_{0.15}Co[NTf_2]_2$ in DMSO-d_6.

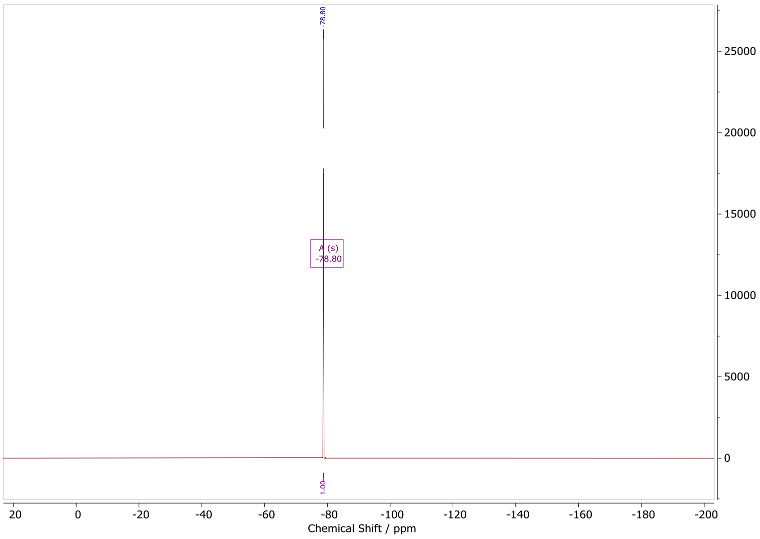

Figure S51 ^{19}F NMR of $[C_8C_1Im][NTf_2]_{0.15}Co[NTf_2]_2$ in DMSO-d_6.

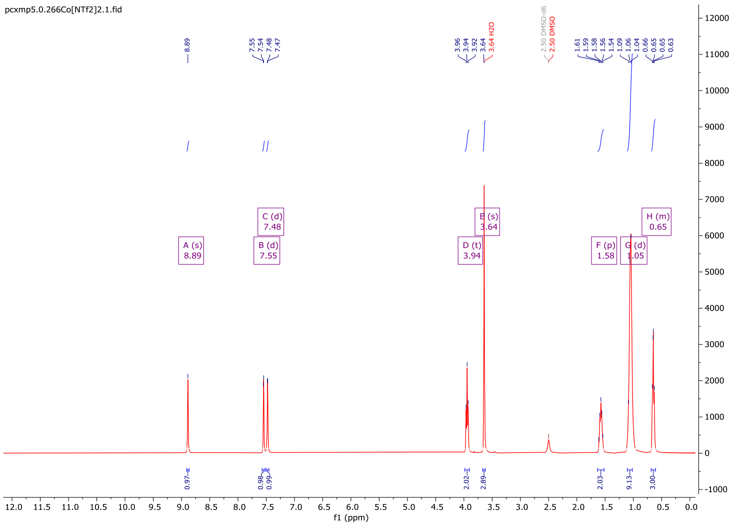

Figure S52 ^{1}H NMR of $[C_8C_1Im][NTf_2]_{0.27}Co[NTf_2]_2$ in DMSO-d_6.

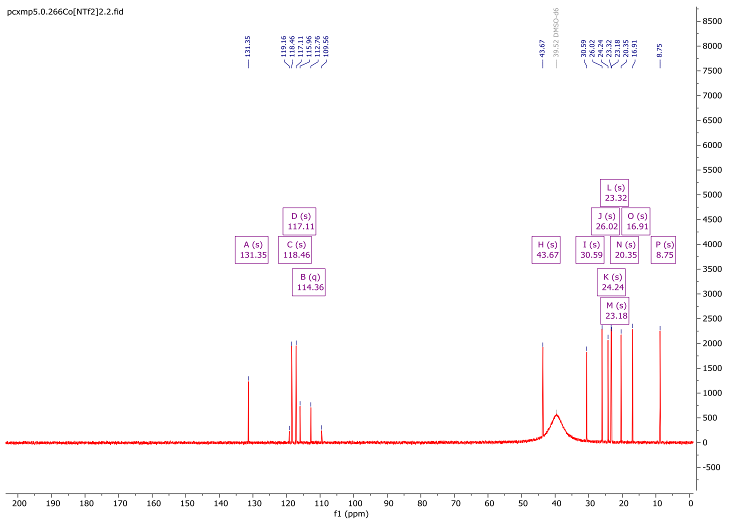

Figure S53 ^{13}C NMR of $[C_8C_1Im][NTf_2]_{0.27}Co[NTf_2]_2$ in DMSO-d_6.

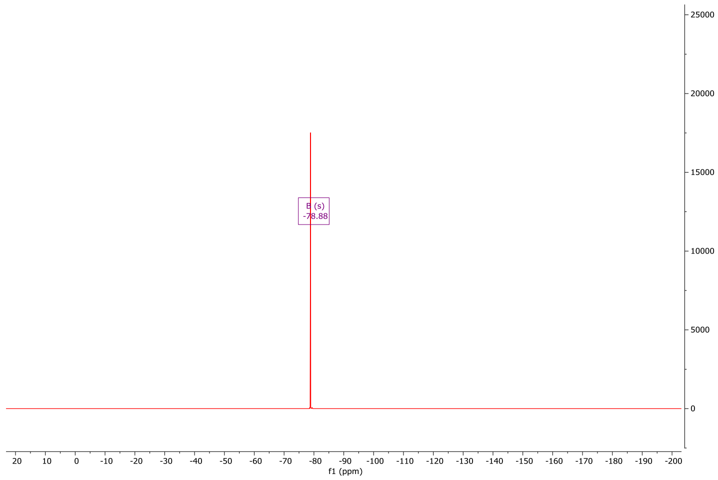

Figure S54 ^{19}F NMR of $[C_8C_1Im][NTf_2]_{0.27}Co[NTf_2]_2$ in DMSO-d_6.

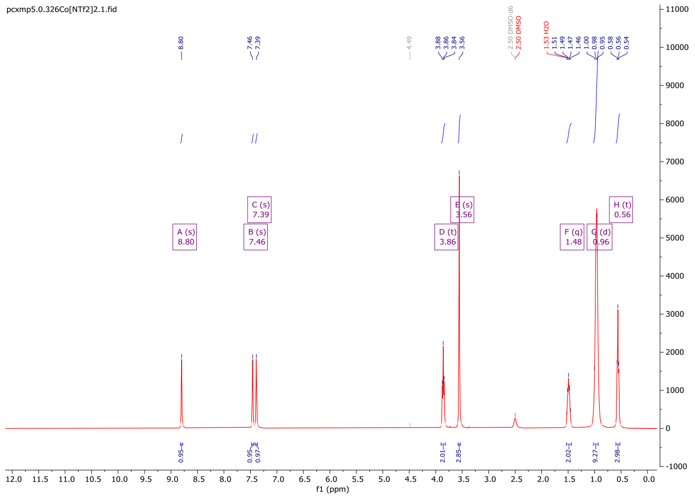

Figure S55 ^{1}H NMR of $[C_8C_1Im][NTf_2]_{0.33}Co[NTf_2]_2$ in DMSO-d_6.

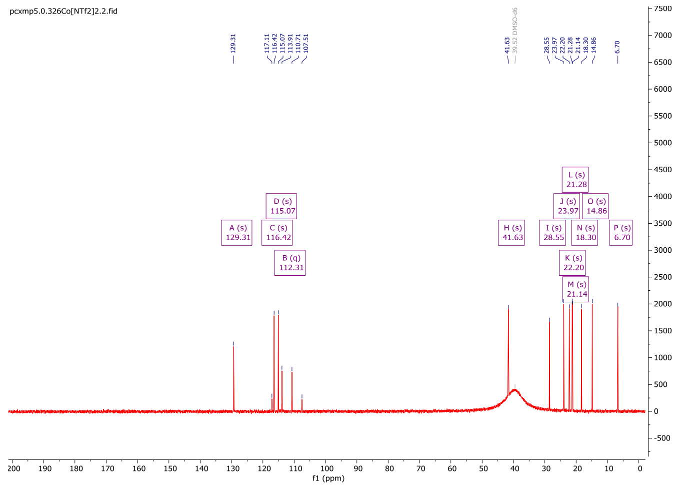

Figure S56 ^{13}C NMR of $[C_8C_1Im][NTf_2]_{0.33}Co[NTf_2]_2$ in DMSO-d_6.

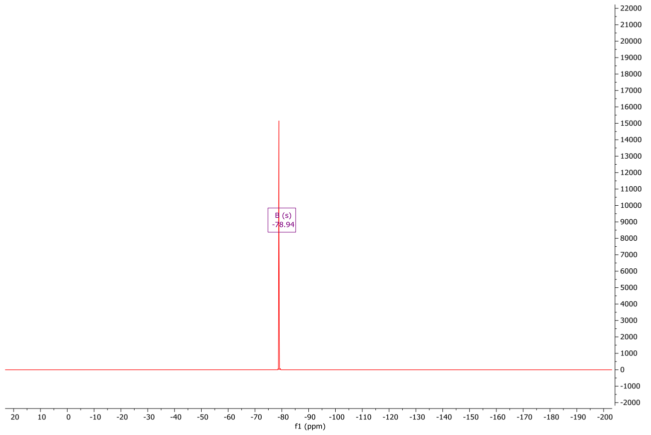

Figure S57 ^{19}F NMR of $[C_8C_1Im][NTf_2]_{0.33}Co[NTf_2]_2$ in DMSO-d_6.

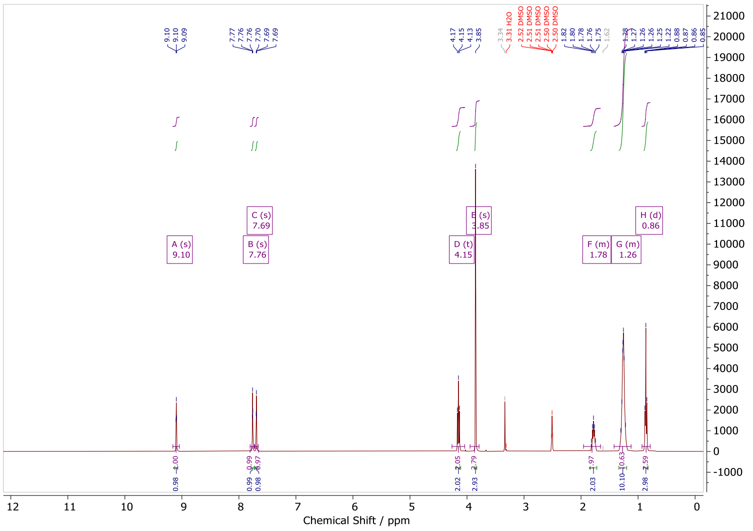

Figure S58 ^{1}H NMR of $[C_8C_1Im][NTf_2]_{0.1}Zn[NTf_2]_2$ in DMSO-d_6.

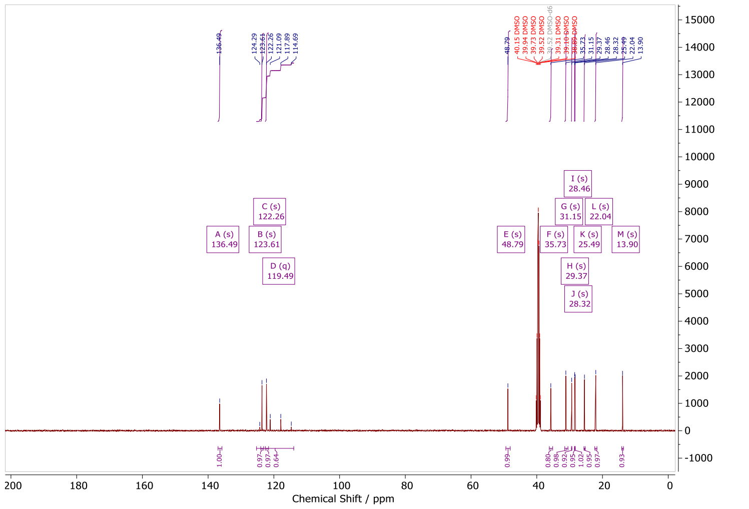

Figure S59 ^{13}C NMR of $[C_8C_1Im][NTf_2]_{0.1}Zn[NTf_2]_2$ in DMSO-d_6.

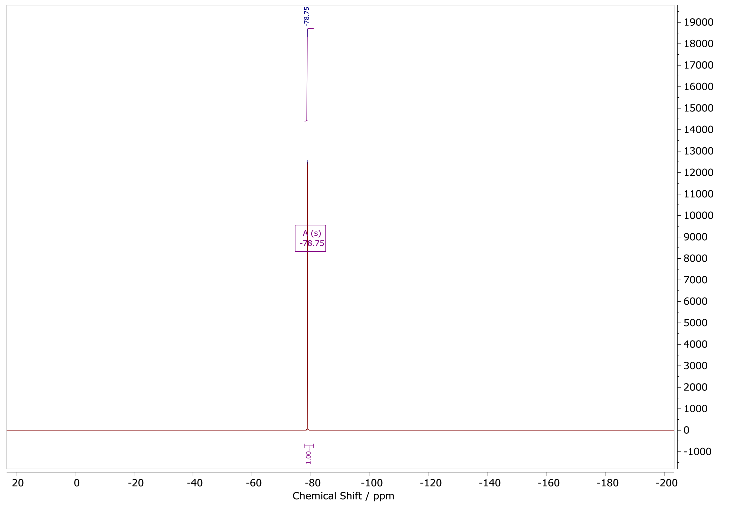

Figure S60 ^{19}F NMR of $[C_8C_1Im][NTf_2]_{0.1}Zn[NTf_2]_2$ in DMSO-d_6.

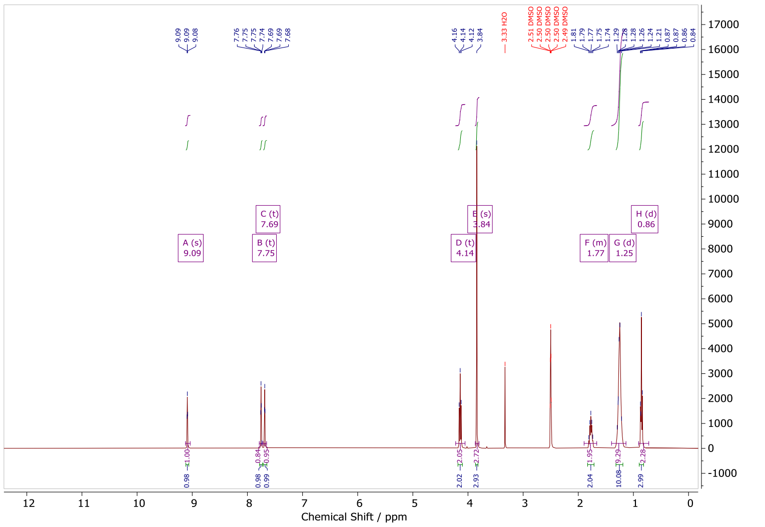

Figure S61 ^{1}H NMR of $[C_8C_1Im][NTf_2]_{0.15}Zn[NTf_2]_2$ in DMSO-d_6.

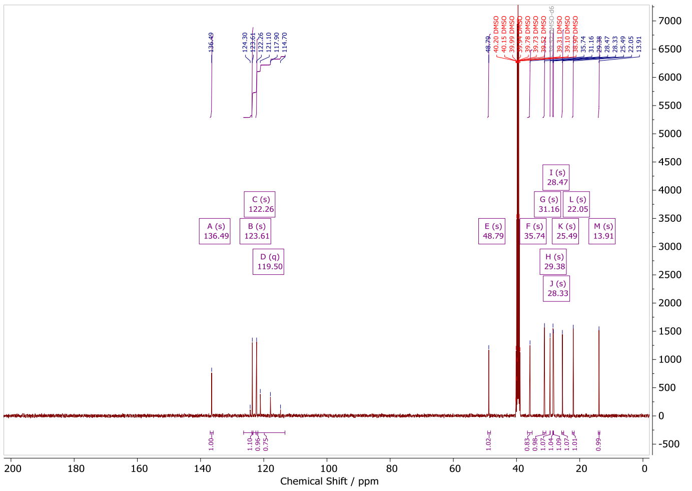

Figure S62 ^{13}C NMR of $[C_8C_1Im][NTf_2]_{0.15}Zn[NTf_2]_2$ in DMSO-d_6.

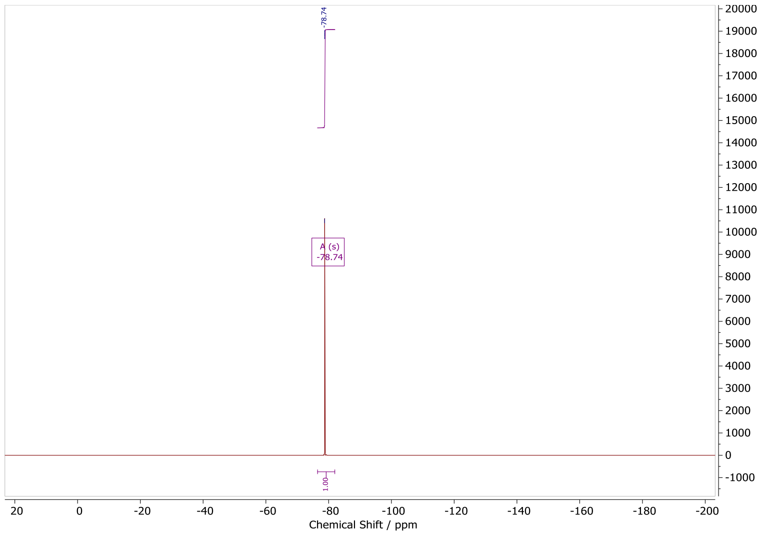

Figure S63 ^{19}F NMR of $[C_8C_1Im][NTf_2]_{0.15}Zn[NTf_2]_2$ in DMSO-d_6.

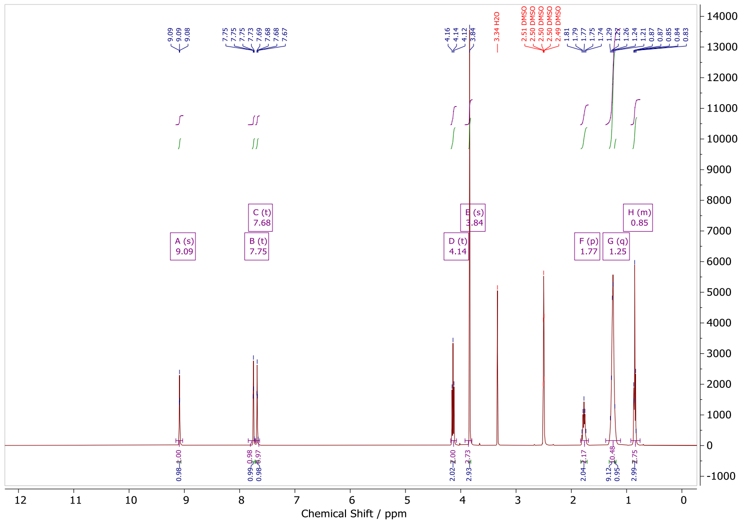

Figure S64 ^1H NMR of $[C_8C_1Im][NTf_2]_{0.27}Zn[NTf_2]_2$ in DMSO-d_6.

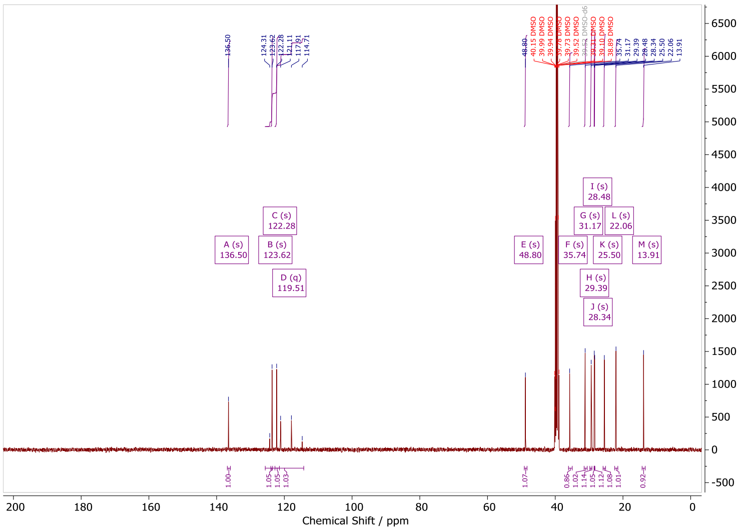

Figure S65 ^{13}C NMR of $[C_8C_1Im][NTf_2]_{0.27}Zn[NTf_2]_2$ in DMSO-d_6.

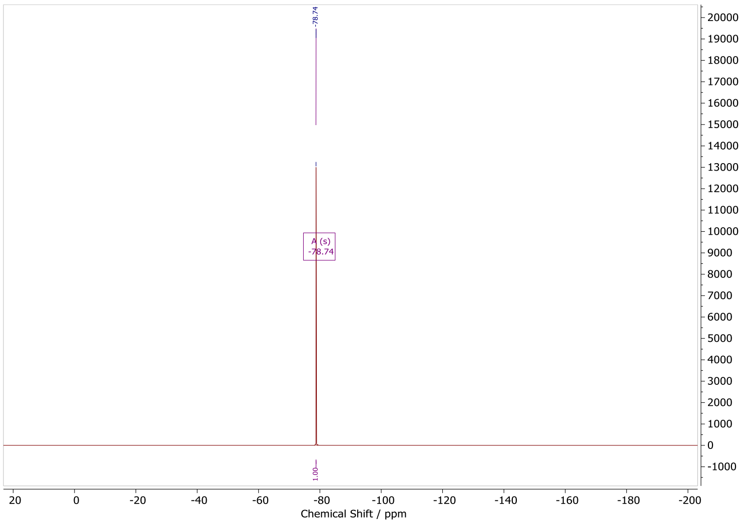

Figure S66 ^{19}F NMR of $[C_8C_1Im][NTf_2]_{0.27}Zn[NTf_2]_2$ in DMSO-d_6.

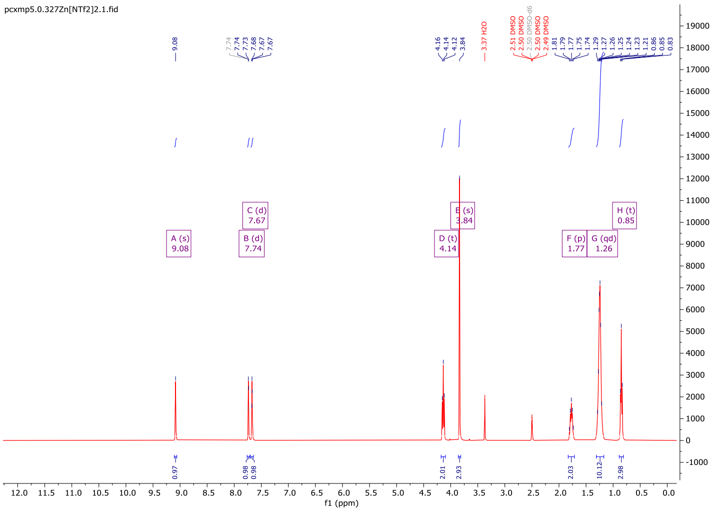

Figure S67 ^{1}H NMR of $[C_8C_1Im][NTf_2]_{0.33}Zn[NTf_2]_2$ in DMSO-d_6.

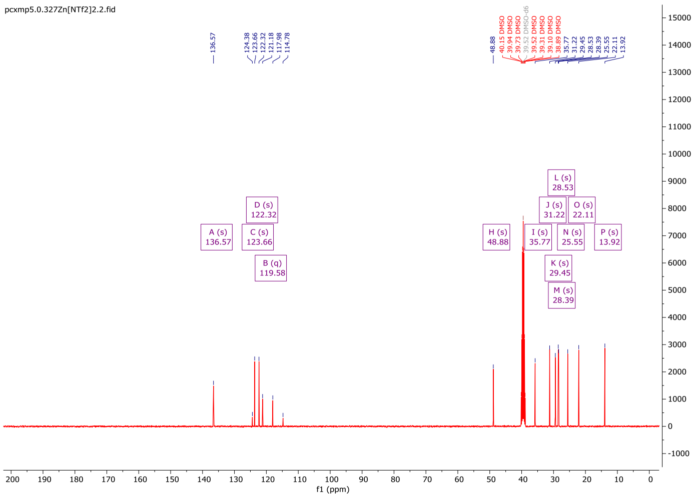

Figure S68 ^{13}C NMR of $[C_8C_1Im][NTf_2]_{0.33}Zn[NTf_2]_2$ in DMSO-d_6.

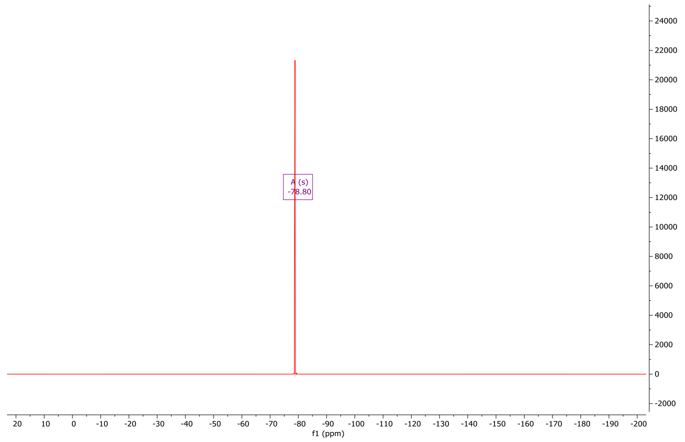

Figure S69 ^{19}F NMR of $[C_8C_1Im][NTf_2]_{0.33}Zn[NTf_2]_2$ in DMSO-d_6.

Table S1 Thermal parameters from TGA and DSC experiments: melting point (T_m), glass transition (T_g), mass loss at 1% ($T_{1\%}$), onset temperature (T_{onset}), liquid range, effective activation energy of decomposition (E_{eff}), temperature at which 1% mass is lost over 10 hours ($T_{0.01/10}$), heat capacity at 20 °C (C_p^o), density at 20 °C (ρ), and viscosity at 20 °C (η).

				Thermal parameters / °C					E_{eff} / kJ mol^{-1}					
Cation	Anion	Metal	χ	T_m	T_g	$T_{1\%}$	T_{onset}	*Liquid Range*	MTGA	Stepwise	$T_{0.01/10}$ / °C	C_p^o / J g^{-1} °C^{-1}	ρ / g mL^{-1}	η / mPa·s
$[C_2C_1Im]$	$[NTf_2]$		0	-18.23		320.8	422.0	339	122.0 ± 1.3	107.4 ± 2.8	317.0		1.524	40.2
		$Zn[NTf_2]_2$	0.33		-60.55	270.0	348.8	331	115.3 ± 0.6	71.9 ± 5.0 / 122.4 ± 4.7	257.9			
$[C_4C_1Im]$	$[NTf_2]$		0		-104 (f)	327.4	407.8	431	118.8 ± 1.0	108 ± 3.1	298.7		1.440	58.4
		$Zn[NTf_2]_2$	0.33		-64.68	293.46	349.7	397	127.1 ± 0.6	92.5 ± 4.0 / 135.4 ± 2.9	301.1			
$[C_8C_1Im]$	$[NTf_2]$		0		-86 (g)	323.9	425.2	410	124.5 ± 1.1	114.4 ± 1.4	298.8		1.324	120.1
		$Zn[NTf_2]_2$	0.1			297.8	386.6			68.0 ± 1.7	298.8	1.30	1.389	221.7
			0.15		-74.3	297.8	369.6	372		57.0 ± 1.6 / 194.1 ± 11.5	285.9	1.25	1.422	315.4
			0.27		-65.3	283.0	376.1	348			278.7	1.11	1.505	841.4
			0.33		-61.6	300.9	362.6	363	136.0 ± 1.0	66.5 ± 2.2 / 125.4 ± 2.5	299.5	1.11	1.546	1490.6
		$Co[NTf_2]_2$	0.1		-78.3	360.25	377.8	439		84.3 ± 4.9 / 149.2 ± 4.2	334.3	1.32	1.387	224.8
			0.15		-74.0	352.4	377.4	426		54.9 ± 3.3 / 164.4 ± 7.9	332.7	1.31	1.418	335.2
			0.27		-64.6	349.5	381.1	414		75.1 ± 6.3 / 151.0 ± 6.5	327.7	1.21	1.496	986.9
			0.33		-61.3	314.6	360.6	376	137.2 ± 0.6	88.9 ± 2.4 / 159.5 ± 3.9	310.3	1.15	1.541	1695.2
		$Mg[NTf_2]_2$	0.1		-78.5	328.76	370.8	407		97.5 ± 3.6	350.7	1.24	1.378	238.8
			0.15		-73.6	325.83	381.5	399		98.2 ± 2.1 / 207.0 ± 18.2	348.1	1.20	1.407	364.0
			0.27		-64.3	325.51	370.0	390		95.4 ± 4.4 / 218.0 ± 22.4	322.2	1.17	1.472	1177.1
			0.32		-58.9	310.06	370.0	369	187.1 ± 31.7	89.9 ± 13.5 / 180.7 ± 11.9	321.0	1.08	1.503	2263.8
		$Li[NTf_2]$	0.1		-79.5	330.9	407.6	410		69.2 ± 2.0 / 113.7 ± 6.0	327.4	1.38	1.350	176.8
			0.15		-78.7	352.3	384.4	431		83.3 ± 5.8 / 142.6 ± 8.3	317.4	1.31	1.366	223.4
			0.27		-71.7	349.3	391.3	421		70.6 ± 4.6 / 144.1 ± 6.3	313.0	1.23	1.404	429.9
			0.33		-67.2	321.24	403.7	388	183.8 ± 5.1	98.9 ± 5.2 / 139.6 ± 1.6	314.7		1.430	710.6
		$Ni[NTf_2]_2$	0.33		-56.8	197.1	359.7	254	130.5 ± 31.7	70.4 ± 4.6 / 144.1 ± 6.3	313.6	1.26	1.531	1137.7

DSC Data

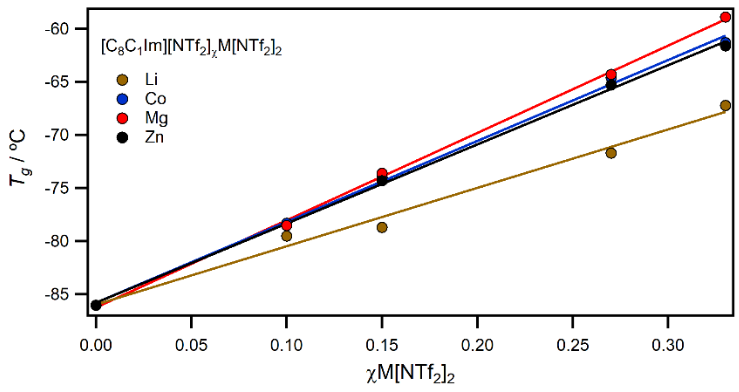

Figure S70 DSC measured T_g values for $[C_8C_1Im][NTf_2]_{\chi}M[NTf_2]_n$ solutions recorded at 10°C min^{-1} in a Tzero alodined aluminium hermetic pan under an N_2 purge gas at 50 mL min^{-1}.

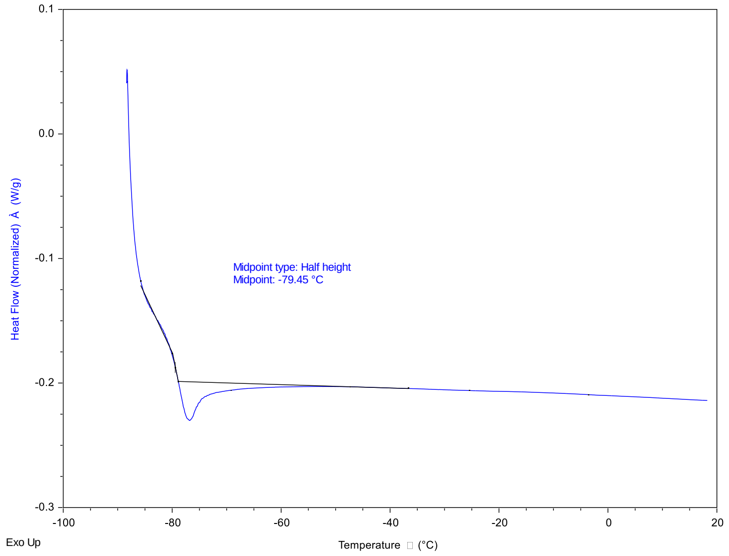

Figure S71 DSC thermogram of $[C_8C_1Im][NTf_2]_{0.1}Li[NTf_2]$ at 10°C min^{-1} in a Tzero alodined aluminium hermetic pan under an N_2 purge gas at 50 mL min^{-1}.

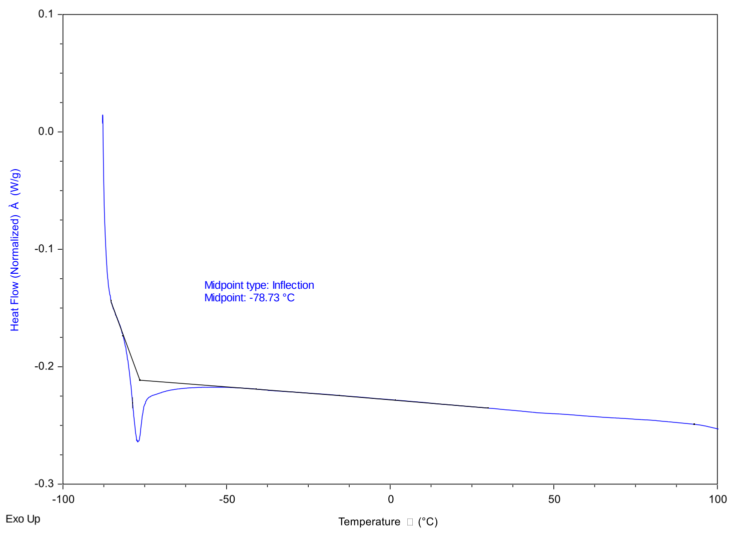

Figure S72 DSC thermogram of $[C_8C_1Im][NTf_2]_{0.15}Li[NTf_2]$ at 10°C min^{-1} in a Tzero alodined aluminium hermetic pan under an N_2 purge gas at 50 mL min^{-1}. [Note: inflection point used because of the proximity of the glass transition to the start-up hook].

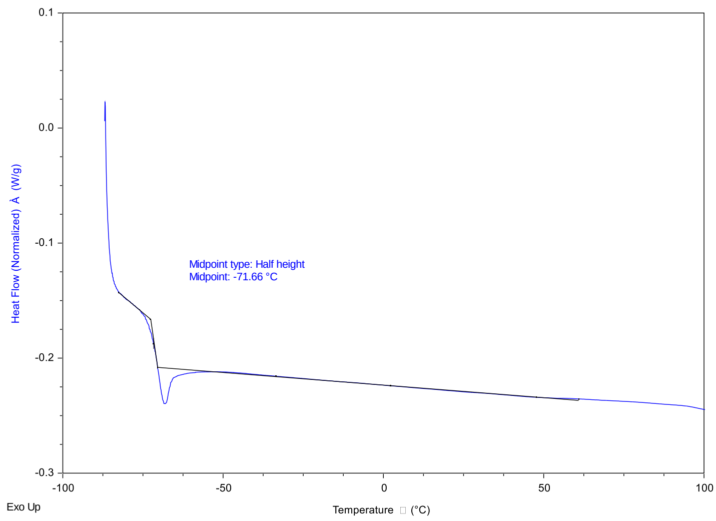

Figure S73 DSC thermogram of $[C_8C_1Im][NTf_2]_{0.27}Li[NTf_2]$ at 10°C min^{-1} in a Tzero alodined aluminium hermetic pan under an N_2 purge gas at 50 mL min^{-1}.

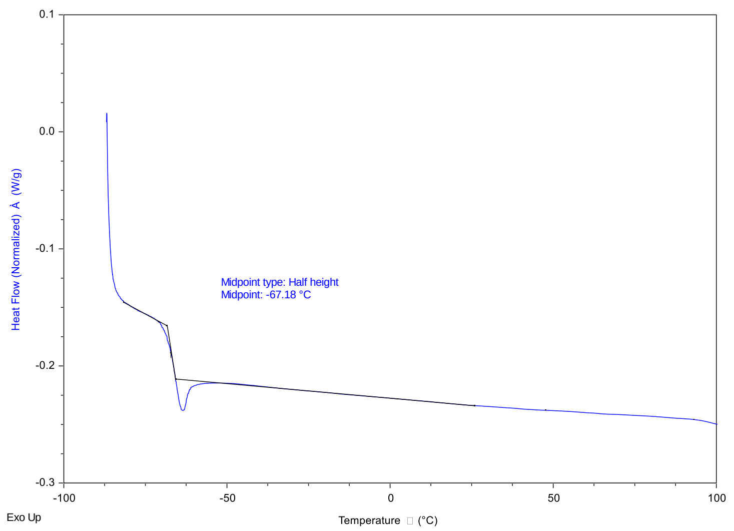

Figure S74 DSC thermogram of $[C_8C_1Im][NTf_2]_{0.33}Li[NTf_2]$ at 10°C min^{-1} in a Tzero alodined aluminium hermetic pan under an N_2 purge gas at 50 mL min^{-1}.

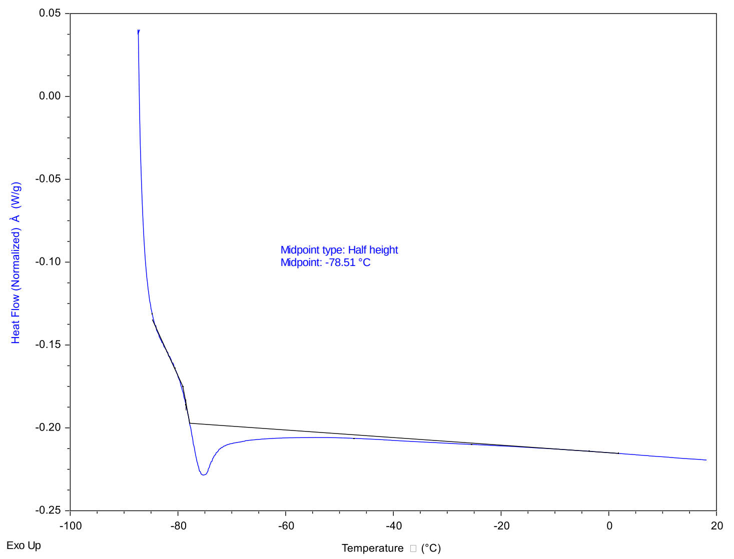

Figure S75 DSC thermogram of $[C_8C_1Im][NTf_2]_{0.1}Mg[NTf_2]_2$ at 10°C min^{-1} in a Tzero alodined aluminium hermetic pan under an N_2 purge gas at 50 mL min^{-1}.

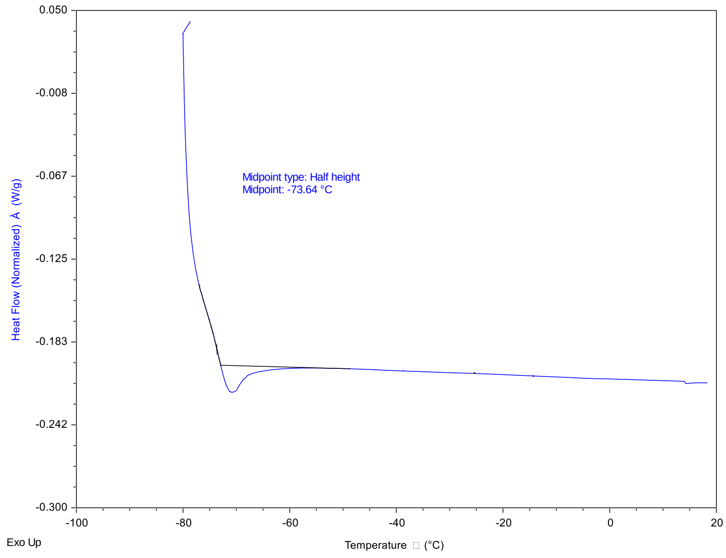

Figure S76 DSC thermogram of $[C_8C_1Im][NTf_2]_{0.15}Mg[NTf_2]_2$ at 10°C min^{-1} in a Tzero alodined aluminium hermetic pan under an N_2 purge gas at 50 mL min^{-1}.

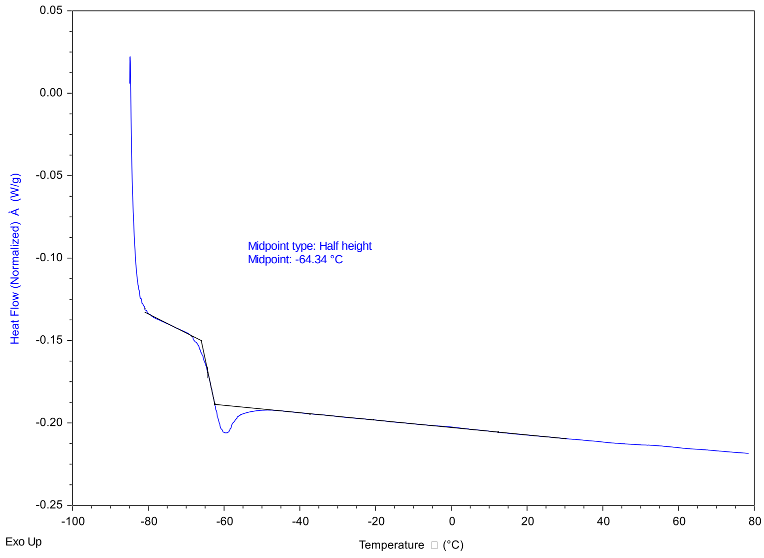

Figure S77 DSC thermogram of $[C_8C_1Im][NTf_2]_{0.27}Mg[NTf_2]_2$ at 10°C min^{-1} in a Tzero alodined aluminium hermetic pan under an N_2 purge gas at 50 mL min^{-1}. [Note: inflection point used because of the proximity of the glass transition to the start-up hook].

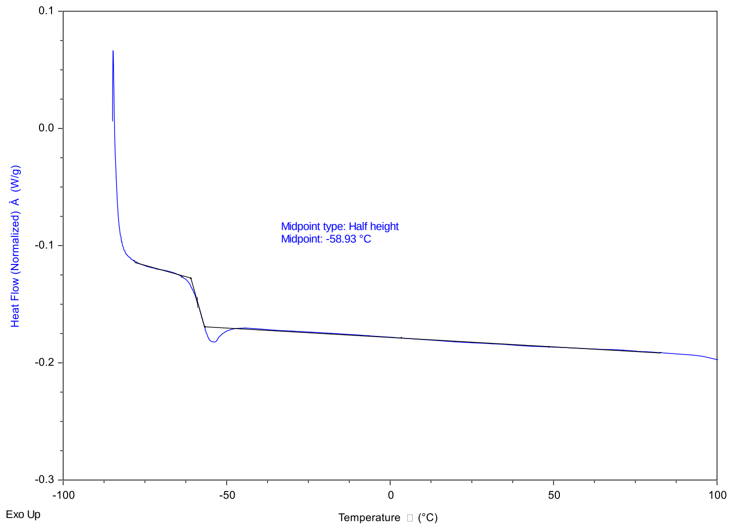

Figure S78 DSC thermogram of $[C_8C_1Im][NTf_2]_{0.32}Mg[NTf_2]_2$ at 10°C min^{-1} in a Tzero alodined aluminium hermetic pan under an N_2 purge gas at 50 mL min^{-1}.

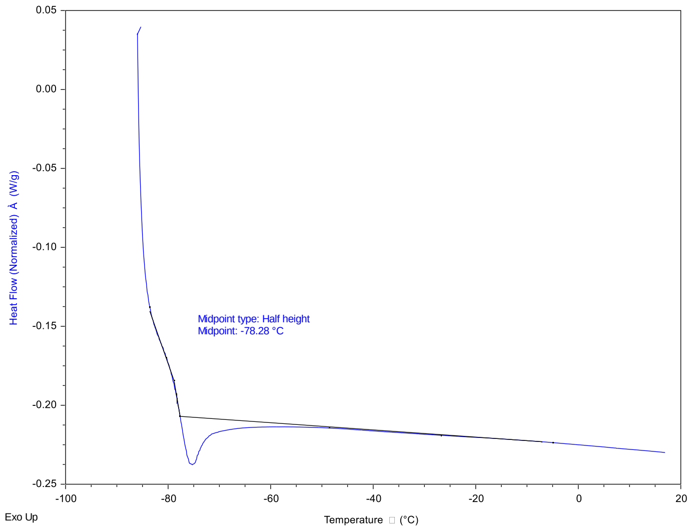

Figure S79 DSC thermogram of $[C_8C_1Im][NTf_2]_{0.1}Co[NTf_2]_2$ at 10°C min^{-1} in a Tzero alodined aluminium hermetic pan under an N_2 purge gas at 50 mL min^{-1}.

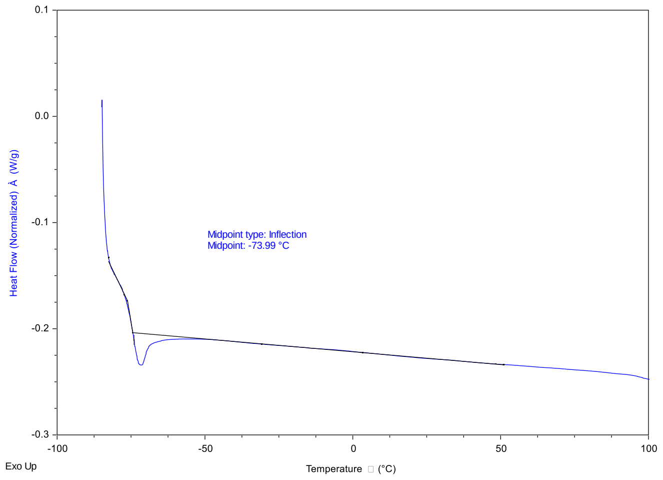

Figure S80 DSC thermogram of $[C_8C_1Im][NTf_2]_{0.15}Co[NTf_2]_2$ at 10°C min^{-1} in a Tzero alodined aluminium hermetic pan under an N_2 purge gas at 50 mL min^{-1}. [Note: inflection point used because of the proximity of the glass transition to the start-up hook].

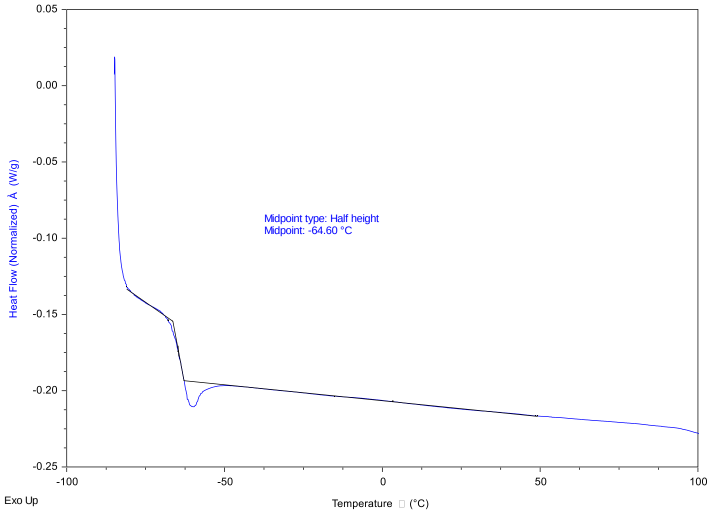

Figure S81 DSC thermogram of $[C_8C_1Im][NTf_2]_{0.27}Co[NTf_2]_2$ at 10°C min^{-1} in a Tzero alodined aluminium hermetic pan under an N_2 purge gas at 50 mL min^{-1}.

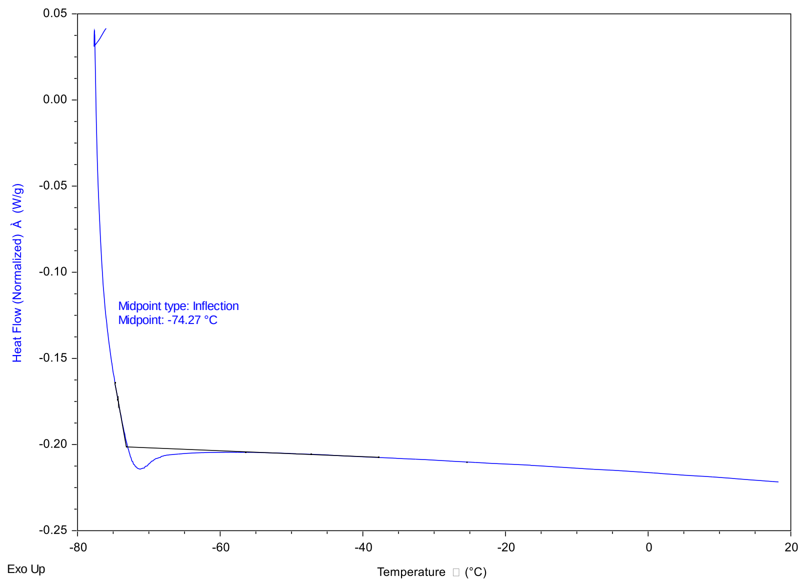

Figure S82 DSC thermogram of $[C_8C_1Im][NTf_2]_{0.15}Zn[NTf_2]_2$ at 10°C min^{-1} in a Tzero alodined aluminium hermetic pan under an N_2 purge gas at 50 mL min^{-1}. [Note: inflection point used because of the proximity of the glass transition to the start-up hook].

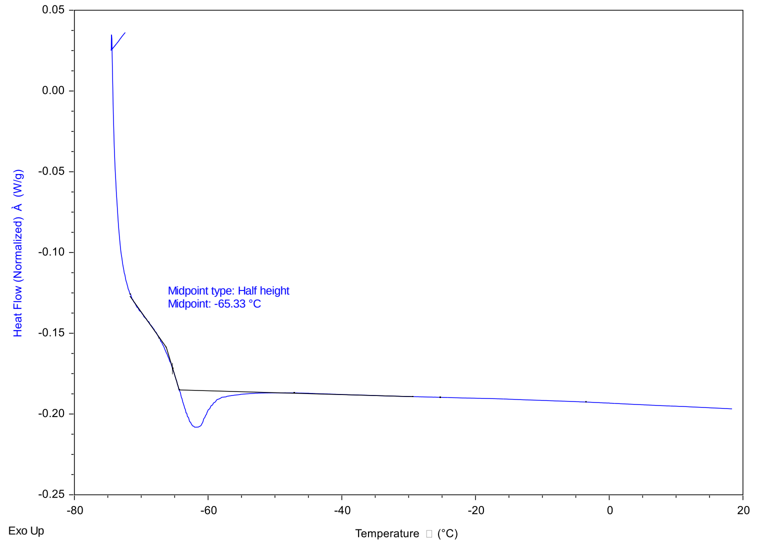

Figure S83 DSC thermogram of $[C_8C_1Im][NTf_2]_{0.27}Zn[NTf_2]_2$ at 10°C min^{-1} in a Tzero alodined aluminium hermetic pan under an N_2 purge gas at 50 mL min^{-1}.

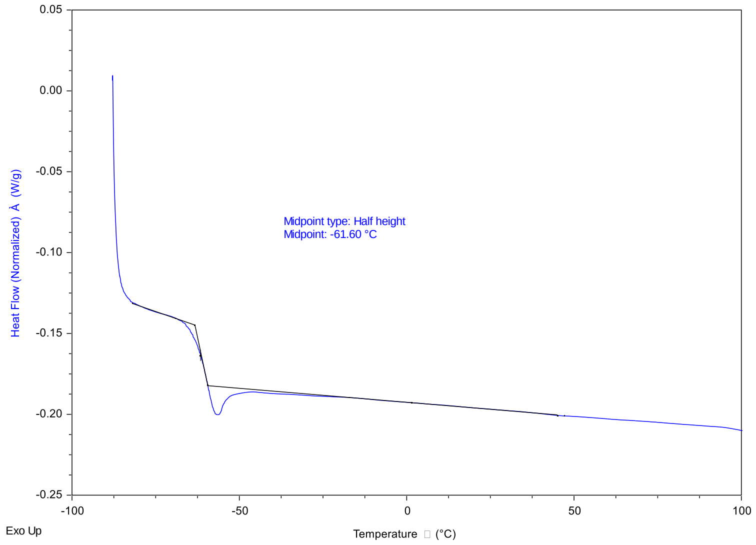

Figure S84 DSC thermogram of $[C_8C_1Im][NTf_2]_{0.33}Zn[NTf_2]_2$ at 10°C min^{-1} in a Tzero alodined aluminium hermetic pan under an N_2 purge gas at 50 mL min^{-1}.

TGA Data

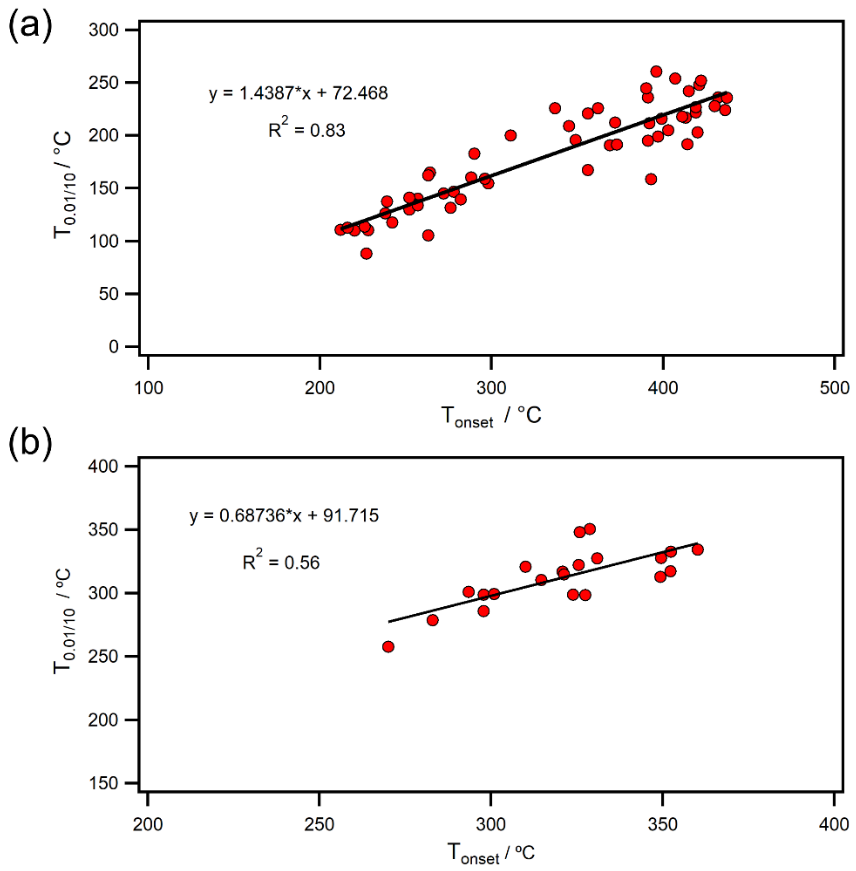

Figure S85 Correlations of long term thermal stability parameter $T_{0.01/10}$ and short term thermal stability parameter $T_{1\%}$ for: (a) data taken from *Ind. Eng. Chem. Res.*, 2014, 53, 20, 8651–8664 for metal-free ionic liquids, and (b) metal ionic liquids from this work.

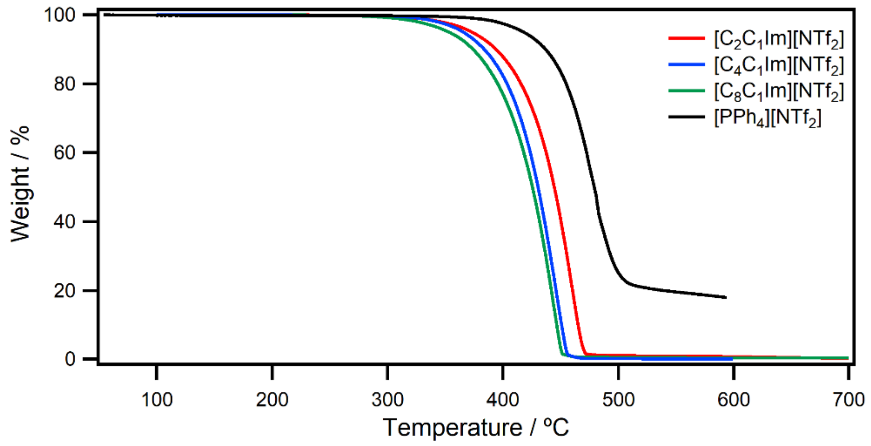

Figure S86 TGA data for monocationic [C_nC_1Im][NTf_2] and [PPh_4][NTf_2] in 50 mL min^{-1} N_2 at 10 °C min^{-1}.

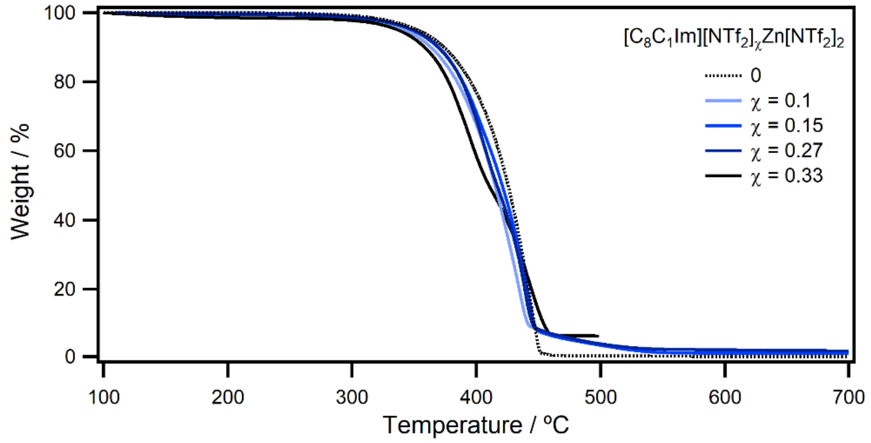

Figure S87 TGA data for [C_8C_1Im][NTf_2]$_x$Zn[NTf_2]$_2$ in 50 mL min^{-1} N_2 at 10 °C min^{-1}.

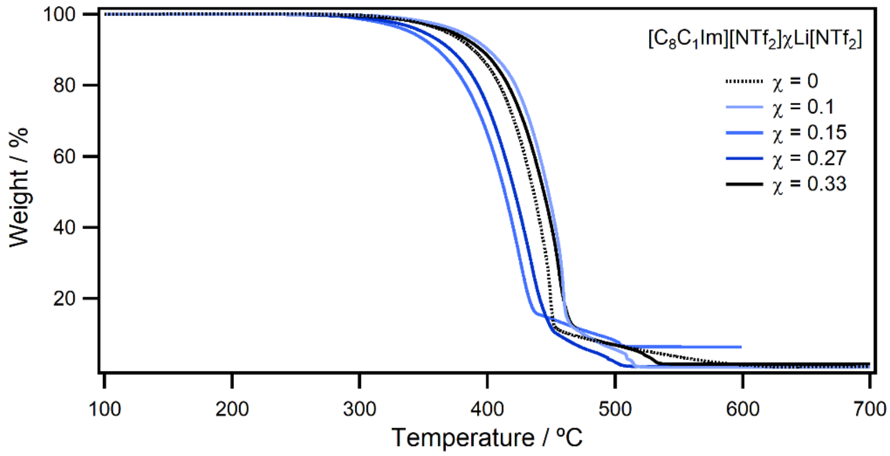

Figure S88 TGA data for [C_8C_1Im][NTf_2]$_x$Li[NTf_2] in 50 mL min^{-1} N_2 at 10 °C min^{-1}.

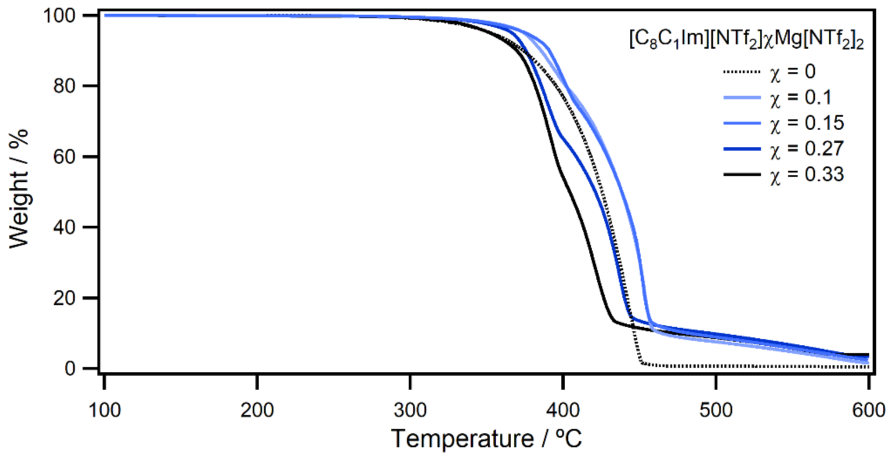

Figure S89 TGA data for $[C_8C_1Im][NTf_2]_xMg[NTf_2]_2$ in 50 mL min^{-1} N_2 at 10 °C min^{-1}.

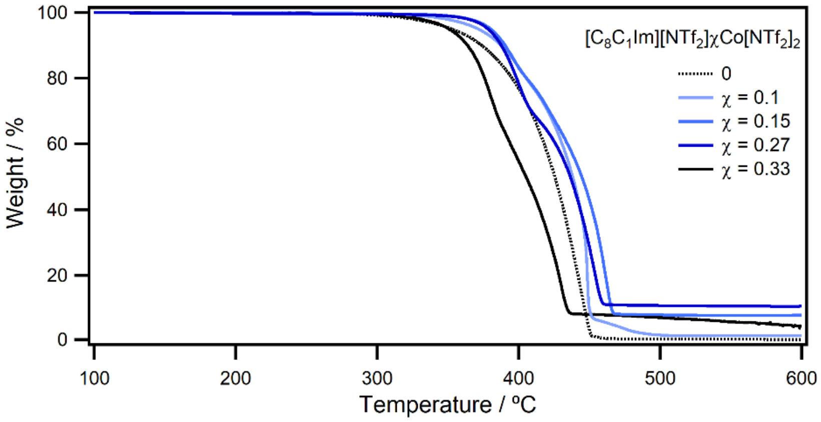

Figure S90 TGA data for $[C_8C_1Im][NTf_2]_xCo[NTf_2]_2$ in 50 mL min^{-1} N_2 at 10 °C min^{-1}.

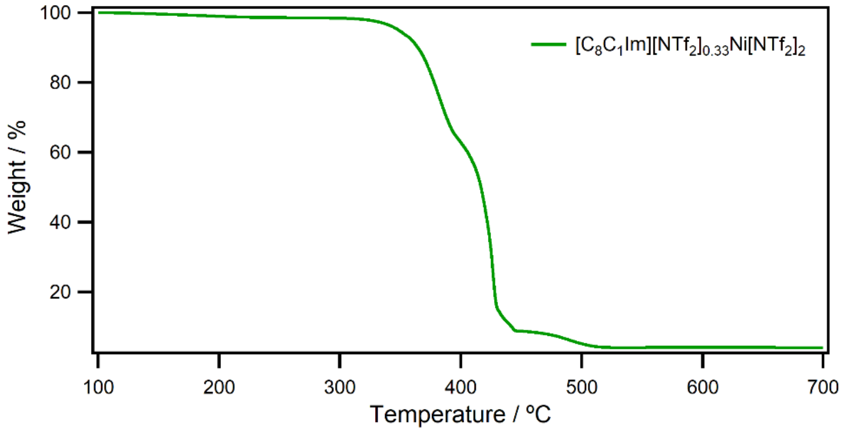

Figure S91 TGA data for $[C_8C_1Im][NTf_2]_xNi[NTf_2]_2$ in 50 mL min^{-1} N_2 at 10 °C min^{-1}.

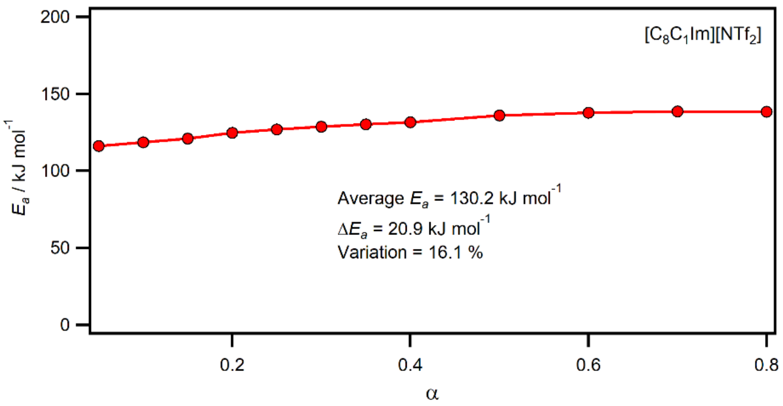

Figure S92 Activation energy with degree of conversion for $[C_8C_1Im][NTf_2]$ in 50 mL min^{-1} N_2 from Flynn-Wall isoconversion.

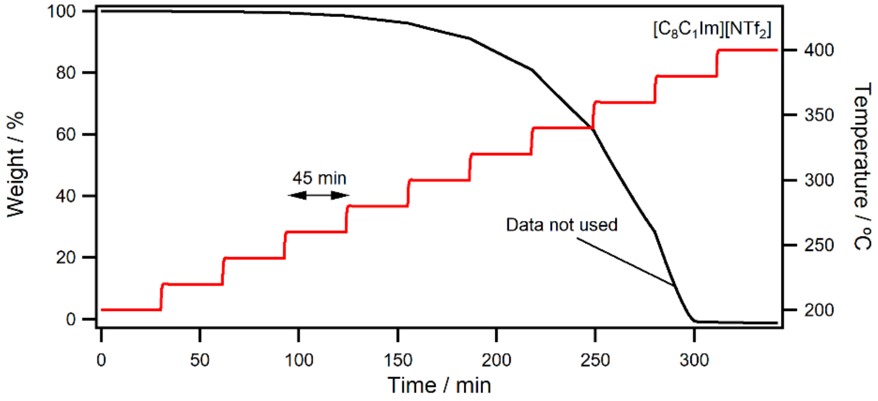

Figure S93 Stepwise TGA data showing weight against time for $[C_8C_1Im][NTf_2]$ in 50 mL min^{-1} N_2 at 10 °C min^{-1}.

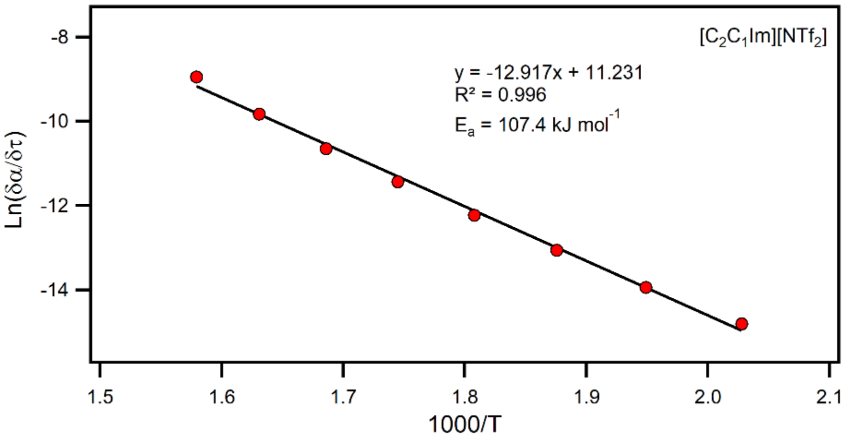

Figure S94 Arrhenius plot for $[C_2C_1Im][NTf_2]$.

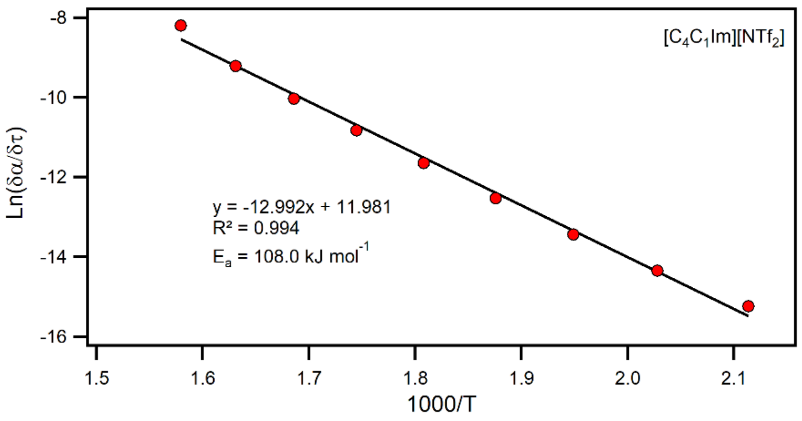

Figure S95 Arrhenius plot for $[C_4C_1Im][NTf_2]$.

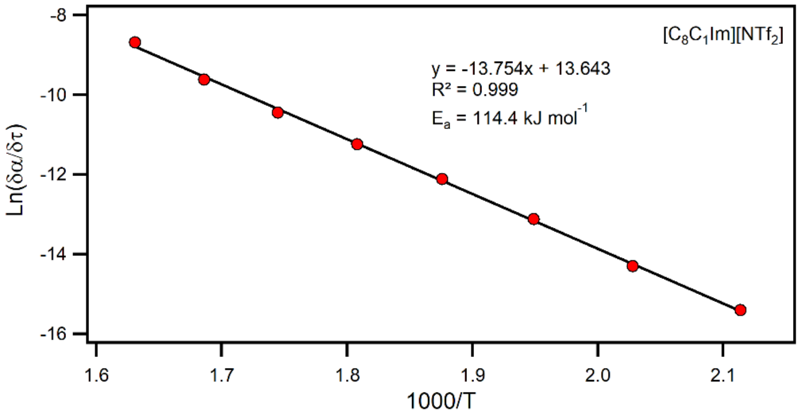

Figure S96 Arrhenius plot for $[C_8C_1Im][NTf_2]$.

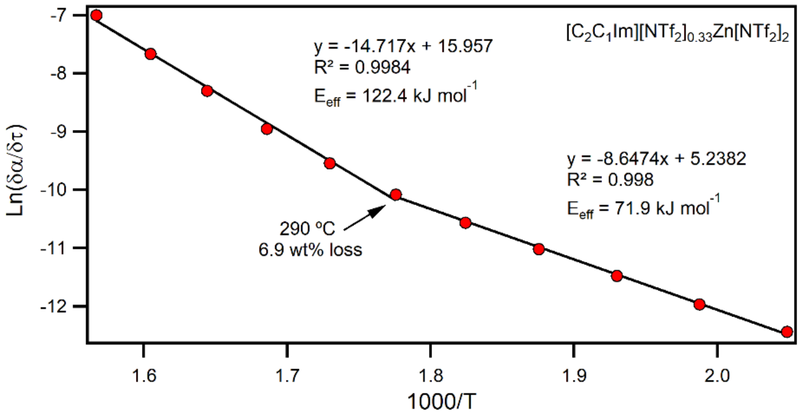

Figure S97 Arrhenius plot for $[C_2C_1Im][NTf_2]_{0.33}Zn[NTf_2]_2$ with two linear fits and the inflection point temperature and weight loss.

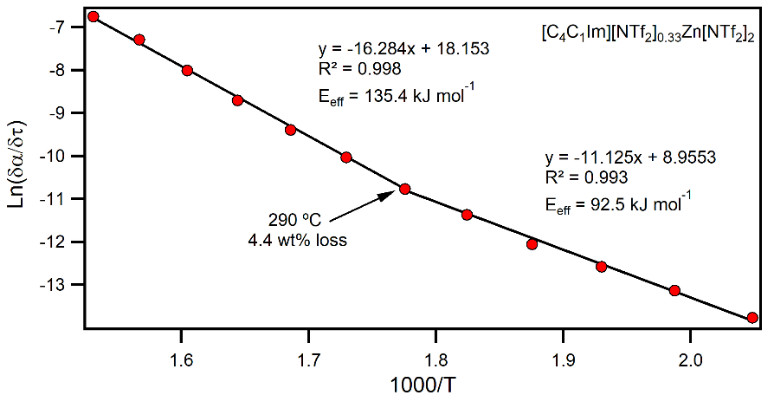

Figure S98 Arrhenius plot for $[C_4C_1Im][NTf_2]_{0.33}Zn[NTf_2]_2$ with two linear fits and the inflection point temperature and weight loss.

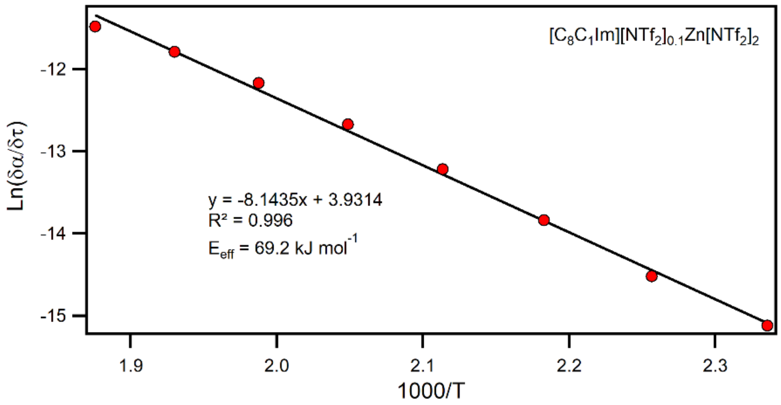

Figure S99 Arrhenius plot for $[C_8C_1Im][NTf_2]_{0.1}Zn[NTf_2]_2$ with two linear fits and the inflection point temperature and weight loss.

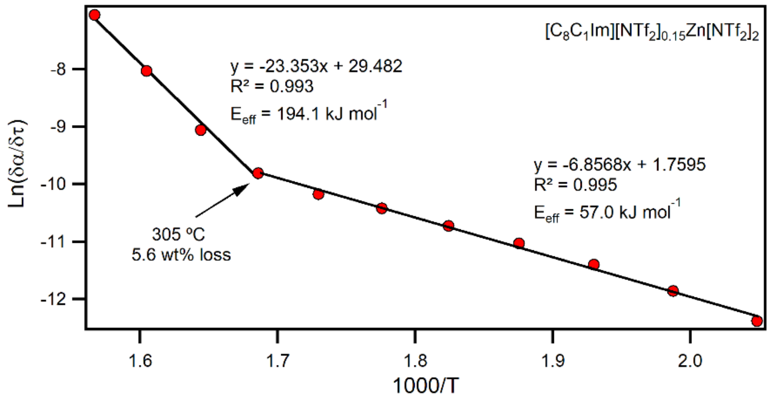

Figure S100 Arrhenius plot for $[C_8C_1Im][NTf_2]_{0.15}Zn[NTf_2]_2$ with two linear fits and the inflection point temperature and weight loss.

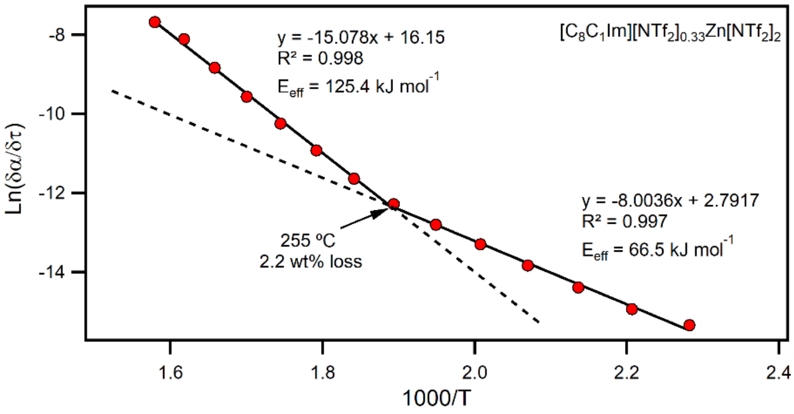

Figure S101 Arrhenius plot for $[C_8C_1Im][NTf_2]_{0.33}Zn[NTf_2]_2$ with two linear fits and the inflection point temperature and weight loss.

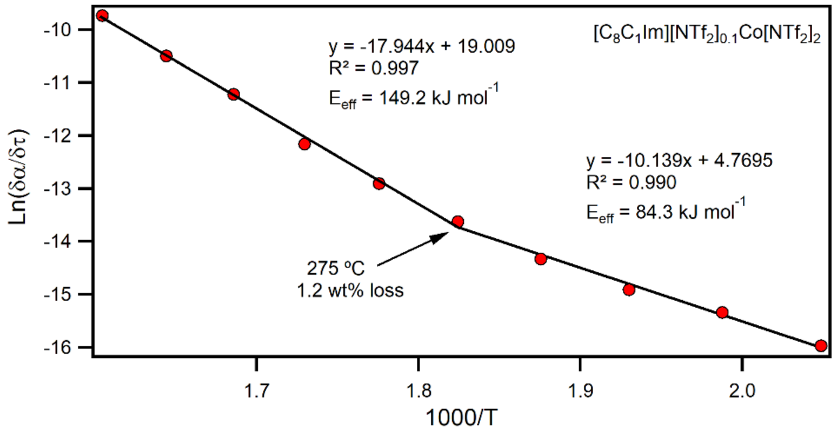

Figure S102 Arrhenius plot for $[C_8C_1Im][NTf_2]_{0.1}Co[NTf_2]_2$ with two linear fits and the inflection point temperature and weight loss.

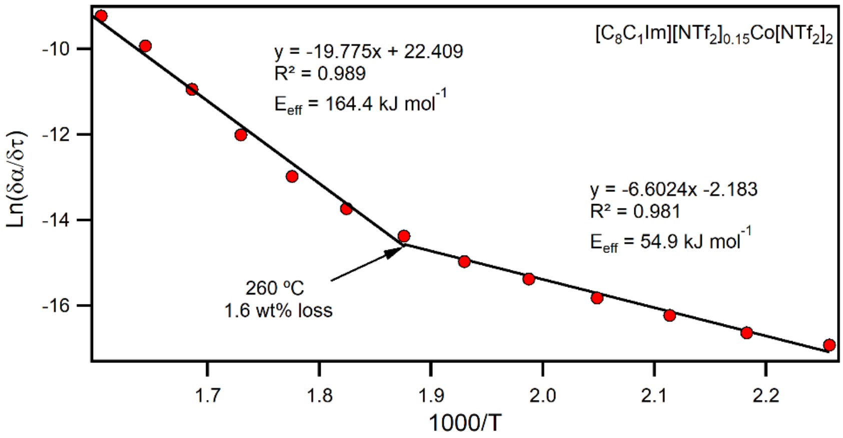

Figure S103 Arrhenius plot for $[C_8C_1Im][NTf_2]_{0.15}Co[NTf_2]_2$ with two linear fits and the inflection point temperature and weight loss.

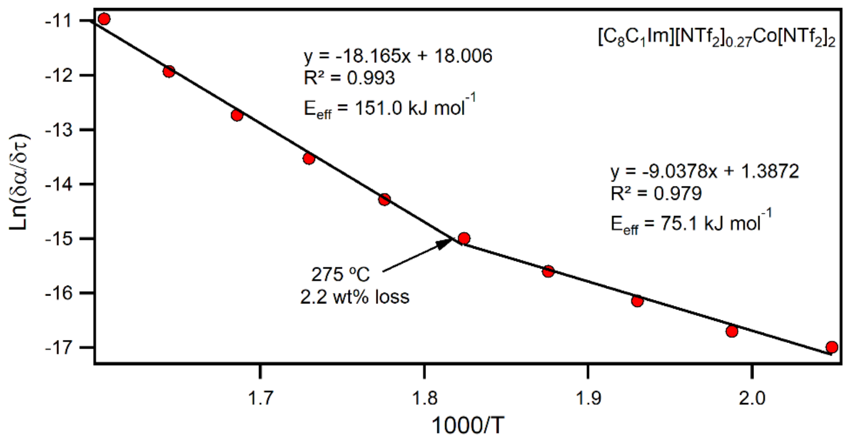

Figure S104 Arrhenius plot for $[C_8C_1Im][NTf_2]_{0.27}Co[NTf_2]_2$ with two linear fits and the inflection point temperature and weight loss.

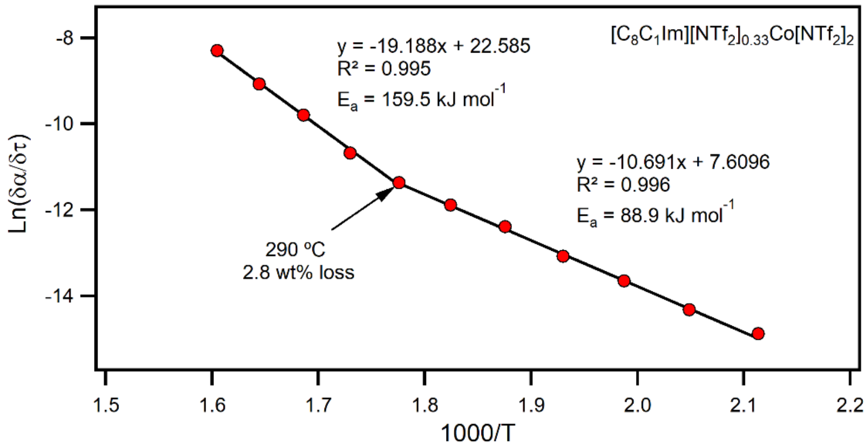

Figure S105 Arrhenius plot for $[C_8C_1Im][NTf_2]_{0.33}Co[NTf_2]_2$ with two linear fits and the inflection point temperature and weight loss.

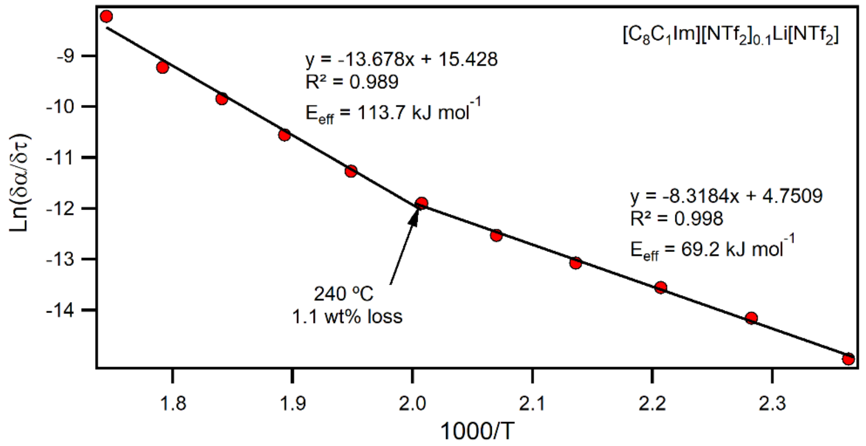

Figure S106 Arrhenius plot for $[C_8C_1Im][NTf_2]_{0.1}Li[NTf_2]$ with two linear fits and the inflection point temperature and weight loss.

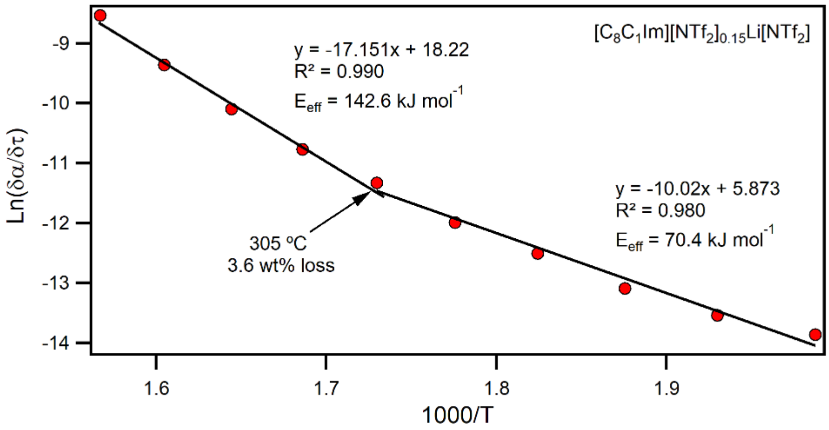

Figure S107 Arrhenius plot for $[C_8C_1Im][NTf_2]_{0.15}Li[NTf_2]$ with two linear fits and the inflection point temperature and weight loss.

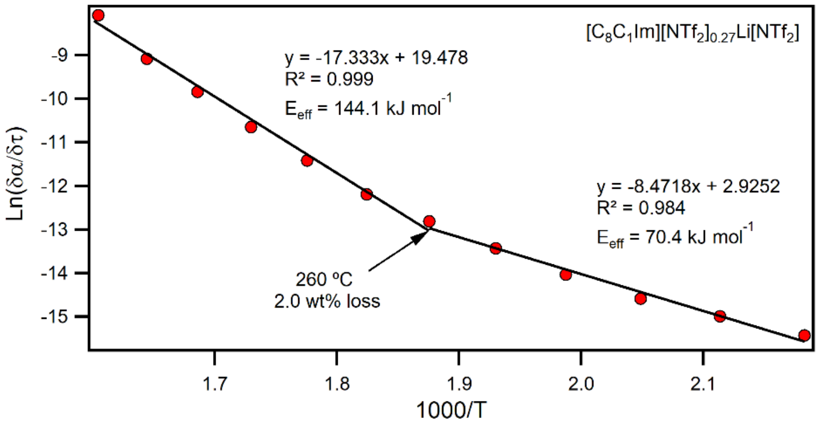

Figure S108 Arrhenius plot for $[C_8C_1Im][NTf_2]_{0.27}Li[NTf_2]$ with two linear fits and the inflection point temperature and weight loss.

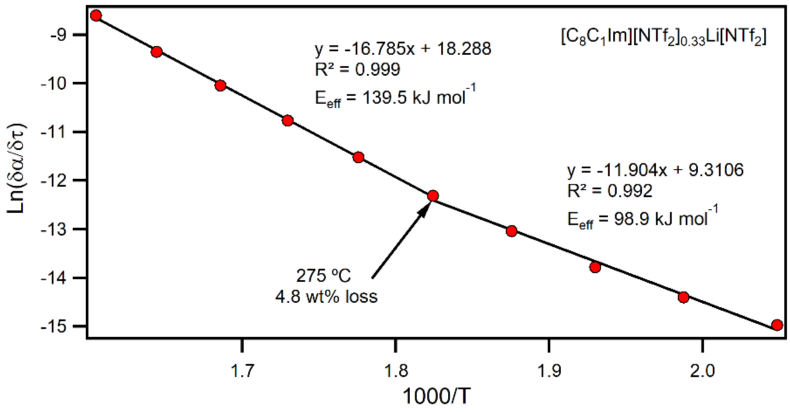

Figure S109 Arrhenius plot for $[C_8C_1Im][NTf_2]_{0.33}Li[NTf_2]$ with two linear fits and the inflection point temperature and weight loss.

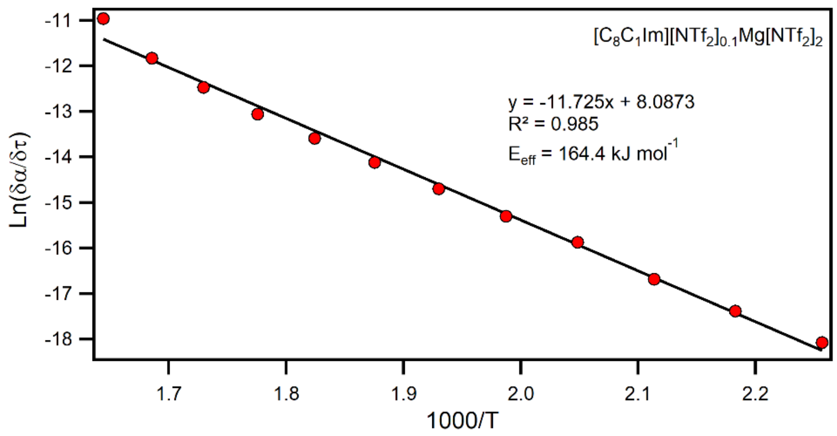

Figure S110 Arrhenius plot for $[C_8C_1Im][NTf_2]_{0.1}Mg[NTf_2]_2$ with two linear fits and the inflection point temperature and weight loss.

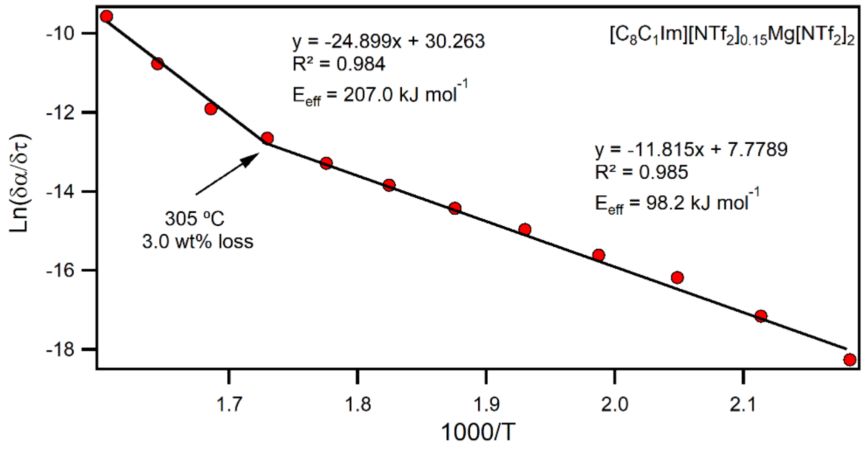

Figure S111 Arrhenius plot for $[C_8C_1Im][NTf_2]_{0.15}Mg[NTf_2]_2$ with two linear fits and the inflection point temperature and weight loss.

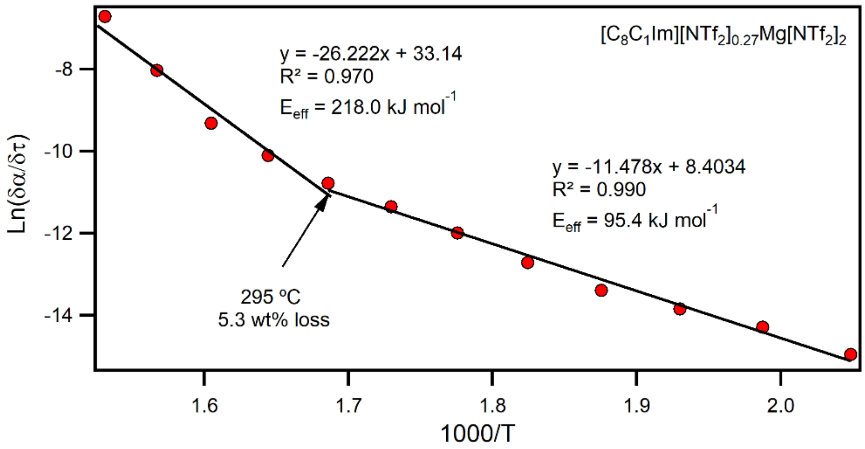

Figure S112 Arrhenius plot for $[C_8C_1Im][NTf_2]_{0.27}Mg[NTf_2]_2$ with two linear fits and the inflection point temperature and weight loss.

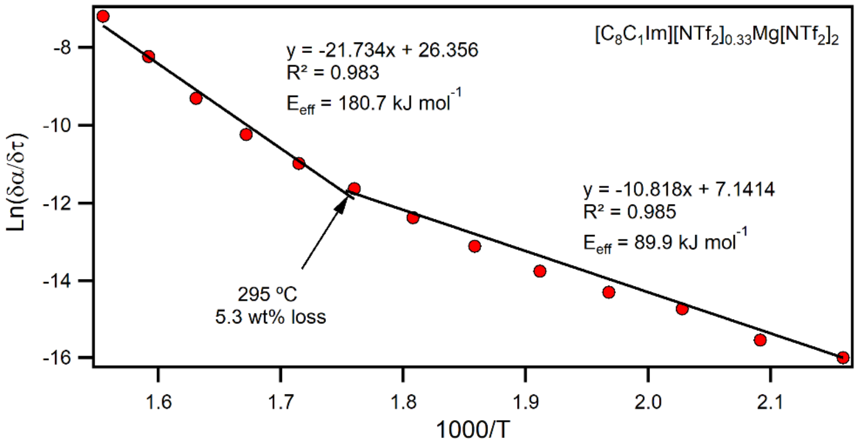

Figure S113 Arrhenius plot for $[C_8C_1Im][NTf_2]_{0.33}Mg[NTf_2]_2$ with two linear fits and the inflection point temperature and weight loss.

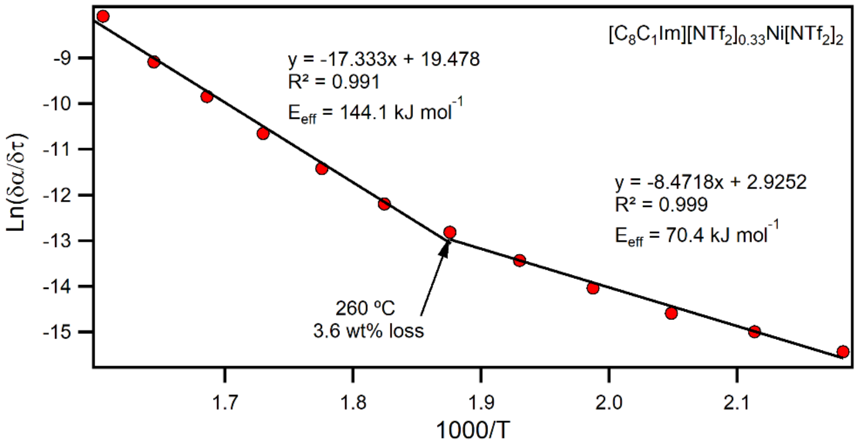

Figure S114 Arrhenius plot for $[C_8C_1Im][NTf_2]_{0.33}Ni[NTf_2]_2$ with two linear fits and the inflection point temperature and weight loss.

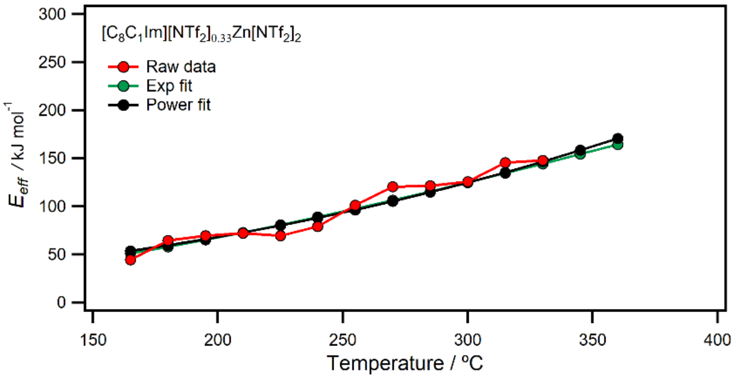

Figure S115 Effective activation energies from stepwise isothermal TGA data of $[C_8C_1Im][NTf_2]_{0.33}Zn[NTf_2]_2$ (15 °C steps for 45-minute isothermal runs). Data is calculated from changing gradients of Arrhenius plots for either the raw data (red) or from exponential (green) or power (black) fits. Average values for data points < 255 °C was 69.9 kJ mol^{-1} compared to 66.5 kJ mol^{-1} for a linear fit, while average values for >255 °C the average value was 130.3 kJ mol^{-1} compared to 125.4 kJ mol^{-1} for a linear fit.

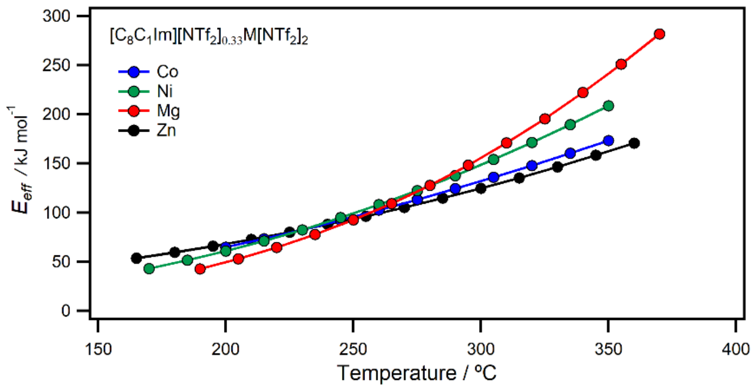

Figure S116 Effective activation energies as a function of temperature from exponential fits of Arrhenius plots for $[C_8C_1Im][NTf_2]_{0.33}M[NTf_2]_2$ where M = Co (blue), Ni (green), Mg (red), and Zn (black).

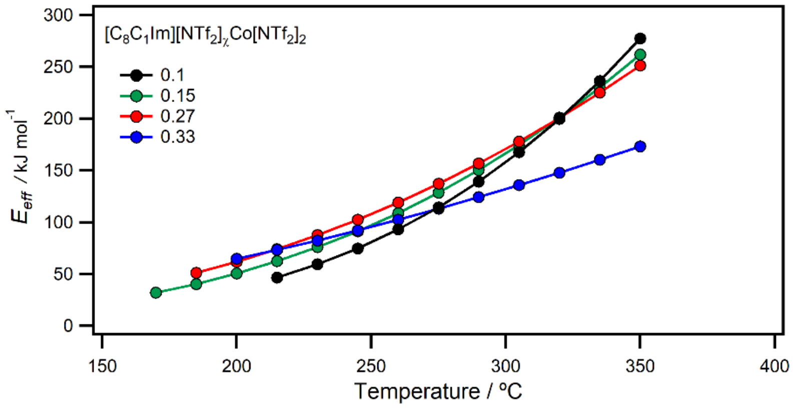

Figure S117 Effective activation energies as a function of temperature from exponential fits of Arrhenius plots for $[C_8C_1Im][NTf_2]_xCo[NTf_2]_2$ for variable mole fractions of metal.

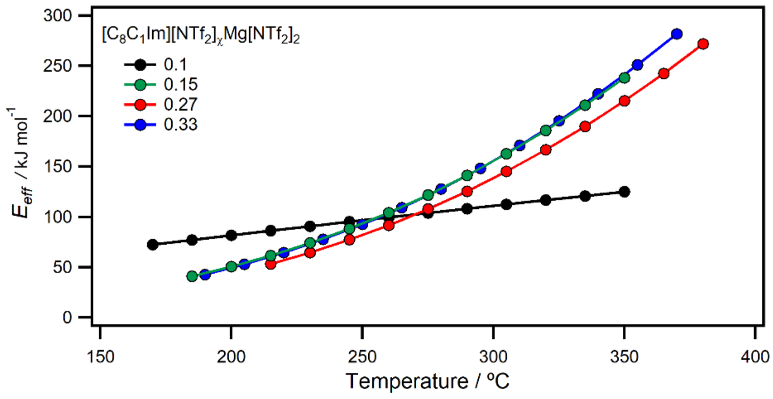

Figure S118 Effective activation energies as a function of temperature from exponential fits of Arrhenius plots for $[C_8C_1Im][NTf_2]_xMg[NTf_2]_2$ for variable mole fractions of metal.

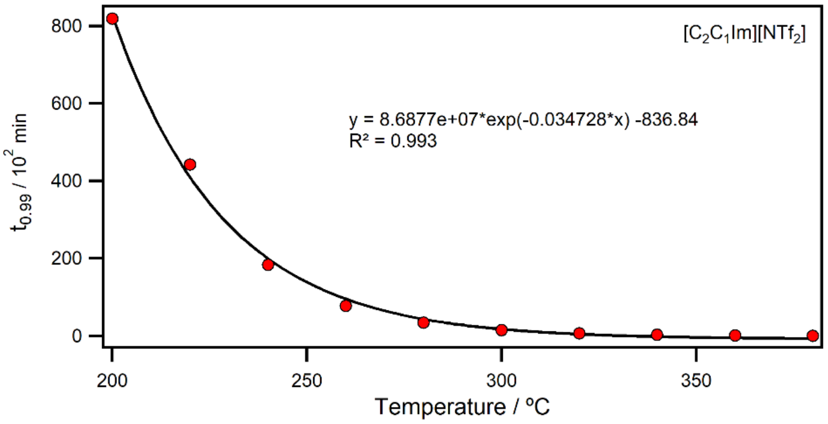

Figure S119 Plot of $t_{0.99}$ versus temperature to calculate the parameter $T_{0.01/10}$ (the temperature at which 1% mass loss occurs over 10 hours of isothermal heating) for $[C_2C_1Im][NTf_2]$.

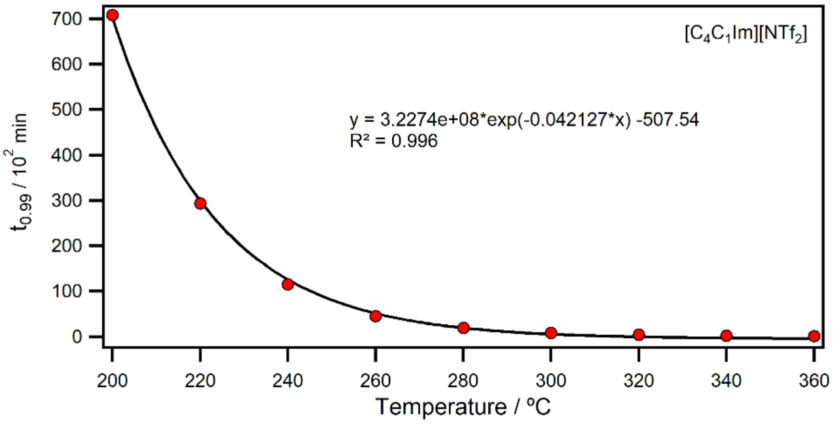

Figure S120 Plot of $t_{0.99}$ versus temperature to calculate the parameter $T_{0.01/10}$ (the temperature at which 1% mass loss occurs over 10 hours of isothermal heating) for $[C_4C_1Im][NTf_2]$.

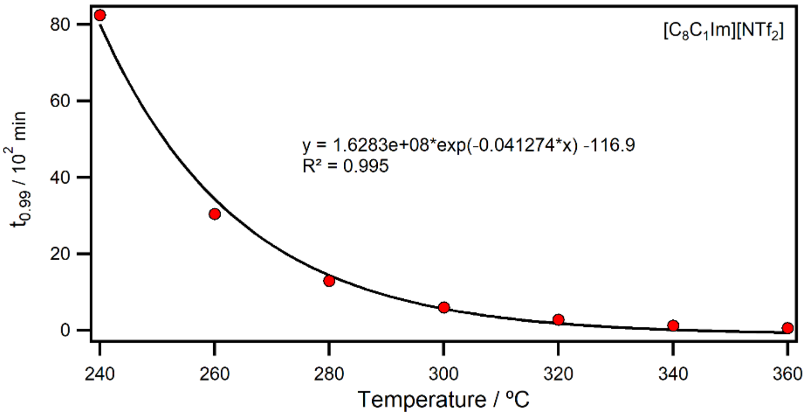

Figure S121 Plot of $t_{0.99}$ versus temperature to calculate the parameter $T_{0.01/10}$ (the temperature at which 1% mass loss occurs over 10 hours of isothermal heating) for $[C_8C_1Im][NTf_2]$.

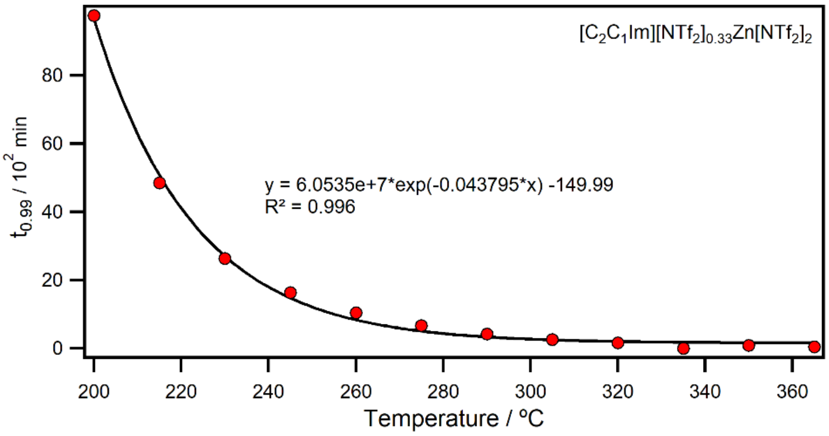

Figure S122 Plot of $t_{0.99}$ versus temperature to calculate the parameter $T_{0.01/10}$ (the temperature at which 1% mass loss occurs over 10 hours of isothermal heating) for $[C_2C_1Im][NTf_2]_{0.33}Zn[NTf_2]_2$.

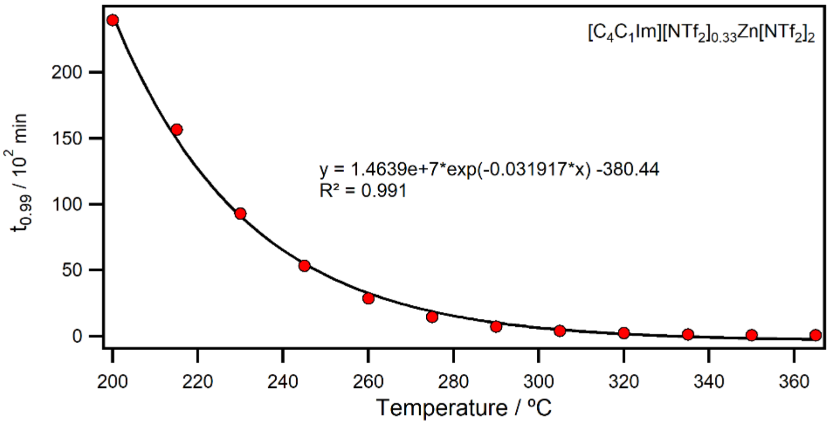

Figure S123 Plot of $t_{0.99}$ versus temperature to calculate the parameter $T_{0.01/10}$ (the temperature at which 1% mass loss occurs over 10 hours of isothermal heating) for $[C_4C_1Im][NTf_2]_{0.33}Zn[NTf_2]_2$.

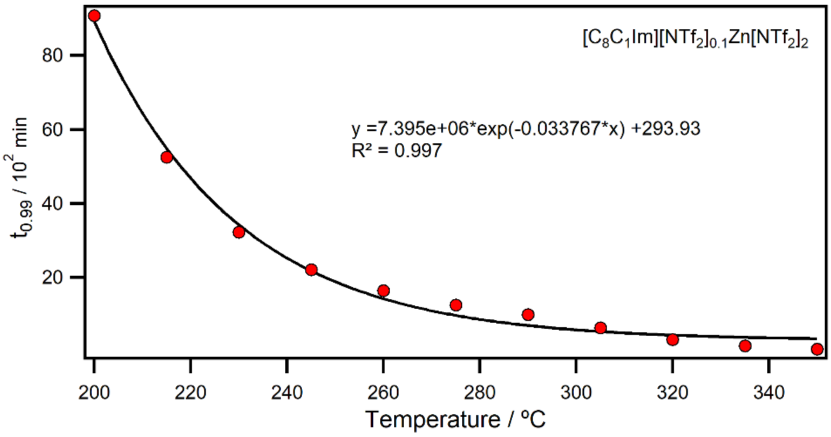

Figure S124 Plot of $t_{0.99}$ versus temperature to calculate the parameter $T_{0.01/10}$ (the temperature at which 1% mass loss occurs over 10 hours of isothermal heating) for $[C_8C_1Im][NTf_2]_{0.1}Zn[NTf_2]_2$.

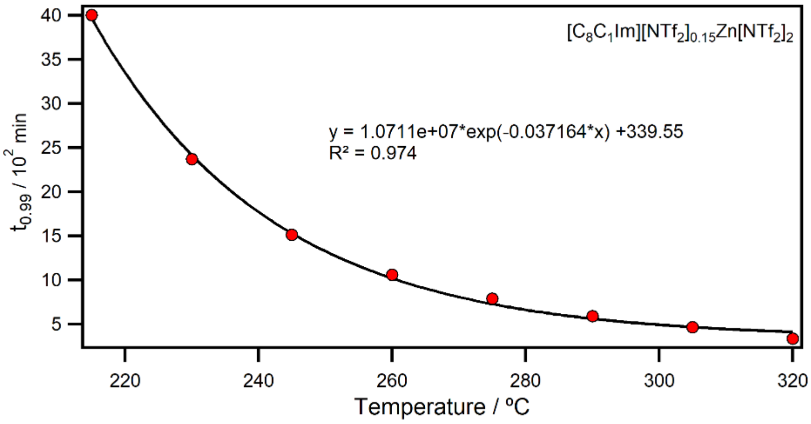

Figure S125 Plot of $t_{0.99}$ versus temperature to calculate the parameter $T_{0.01/10}$ (the temperature at which 1% mass loss occurs over 10 hours of isothermal heating) for $[C_8C_1Im][NTf_2]_{0.15}Zn[NTf_2]_2$.

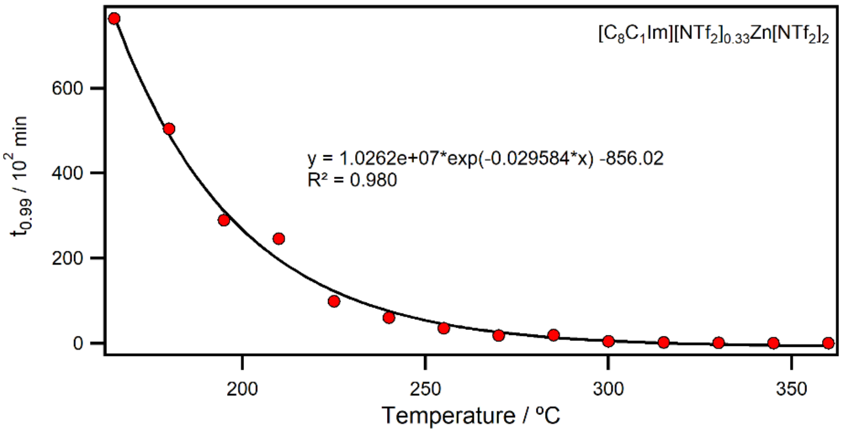

Figure S126 Plot of $t_{0.99}$ versus temperature to calculate the parameter $T_{0.01/10}$ (the temperature at which 1% mass loss occurs over 10 hours of isothermal heating) for $[C_8C_1Im][NTf_2]_{0.33}Zn[NTf_2]_2$.

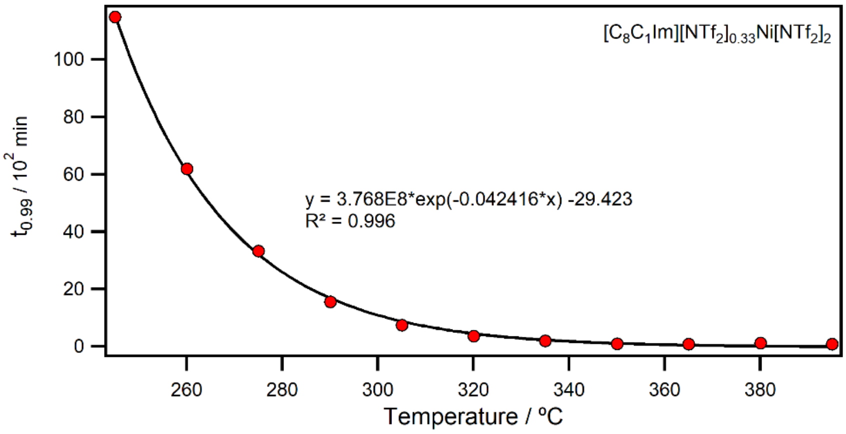

Figure S127 Plot of $t_{0.99}$ versus temperature to calculate the parameter $T_{0.01/10}$ (the temperature at which 1% mass loss occurs over 10 hours of isothermal heating) for $[C_8C_1Im][NTf_2]_{0.33}Ni[NTf_2]_2$.

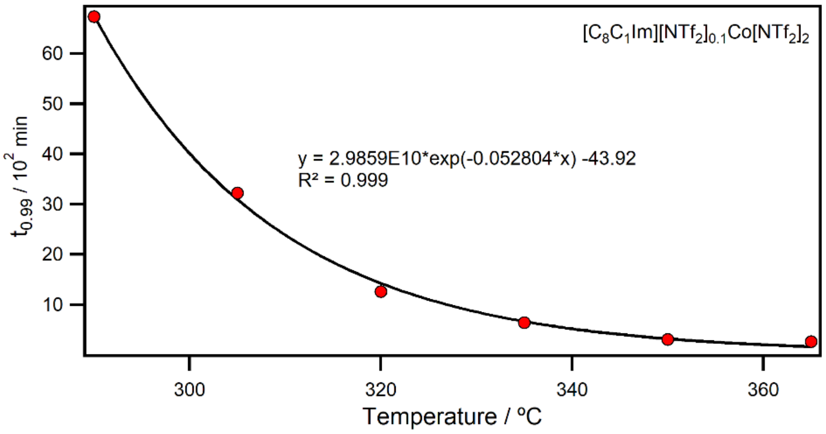

Figure S128 Plot of $t_{0.99}$ versus temperature to calculate the parameter $T_{0.01/10}$ (the temperature at which 1% mass loss occurs over 10 hours of isothermal heating) for $[C_8C_1Im][NTf_2]_{0.1}Co[NTf_2]_2$.

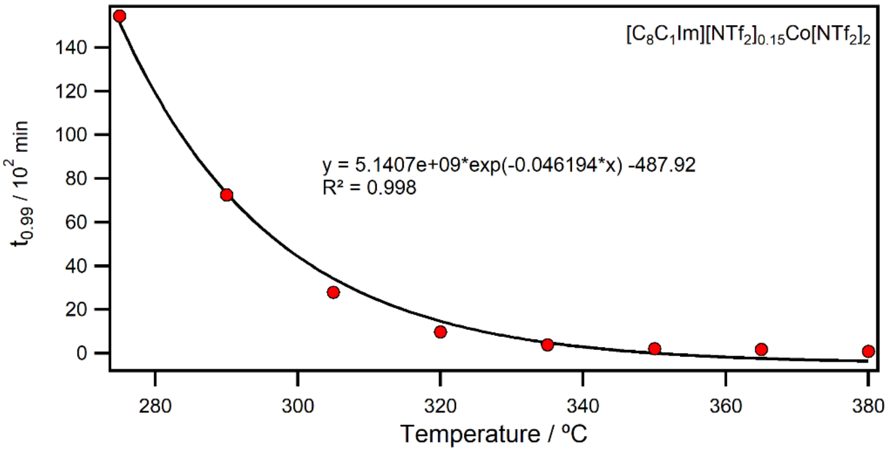

Figure S129 Plot of $t_{0.99}$ versus temperature to calculate the parameter $T_{0.01/10}$ (the temperature at which 1% mass loss occurs over 10 hours of isothermal heating) for $[C_8C_1Im][NTf_2]_{0.15}Co[NTf_2]_2$.

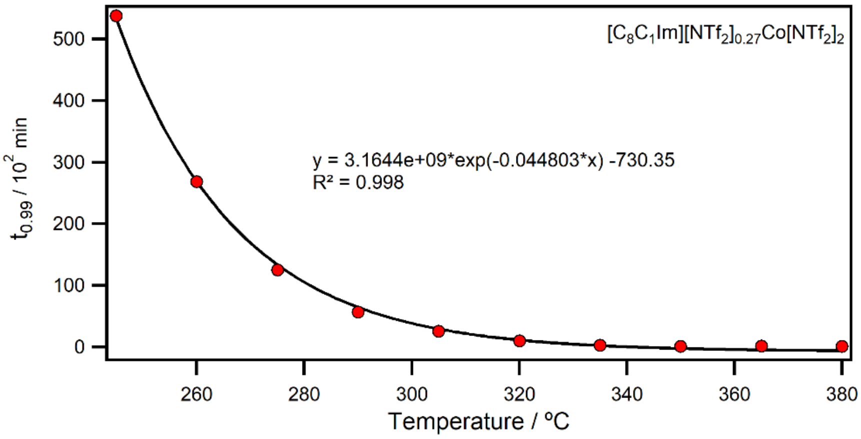

Figure S130 Plot of $t_{0.99}$ versus temperature to calculate the parameter $T_{0.01/10}$ (the temperature at which 1% mass loss occurs over 10 hours of isothermal heating) for $[C_8C_1Im][NTf_2]_{0.27}Co[NTf_2]_2$.

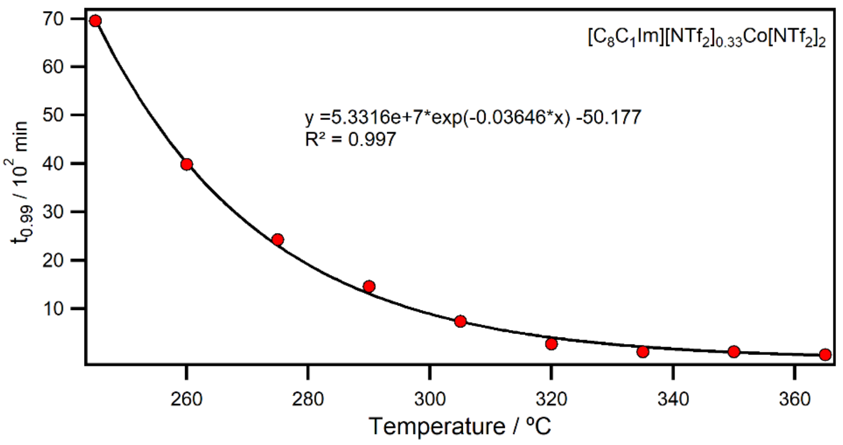

Figure S131 Plot of $t_{0.99}$ versus temperature to calculate the parameter $T_{0.01/10}$ (the temperature at which 1% mass loss occurs over 10 hours of isothermal heating) for $[C_8C_1Im][NTf_2]_{0.33}Co[NTf_2]_2$.

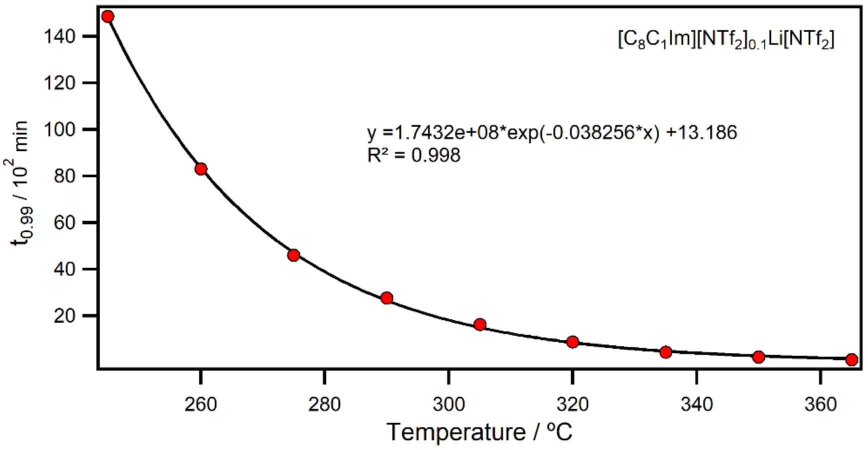

Figure S132 Plot of $t_{0.99}$ versus temperature to calculate the parameter $T_{0.01/10}$ (the temperature at which 1% mass loss occurs over 10 hours of isothermal heating) for $[C_8C_1Im][NTf_2]_{0.1}Li[NTf_2]$.

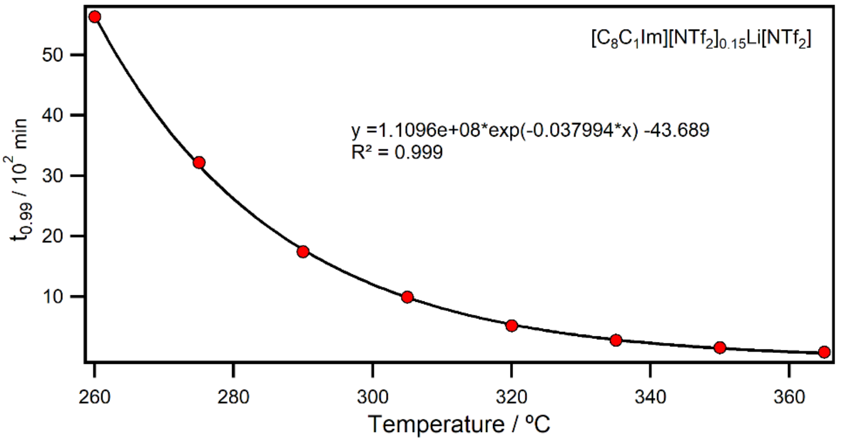

Figure S133 Plot of $t_{0.99}$ versus temperature to calculate the parameter $T_{0.01/10}$ (the temperature at which 1% mass loss occurs over 10 hours of isothermal heating) for $[C_8C_1Im][NTf_2]_{0.15}Li[NTf_2]$.

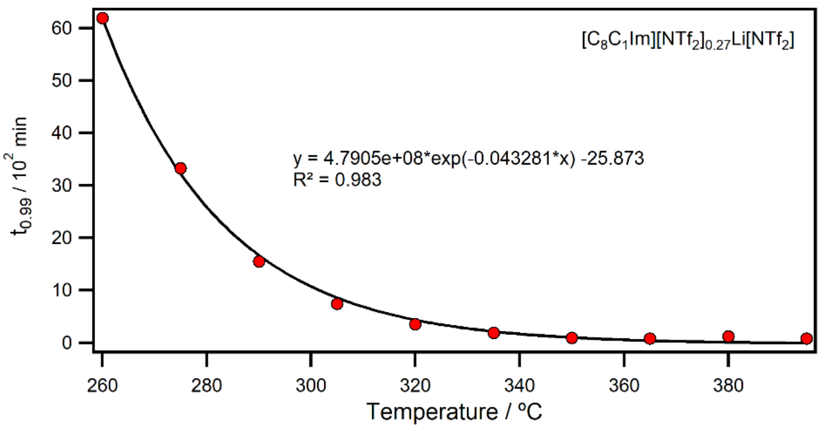

Figure S134 Plot of $t_{0.99}$ versus temperature to calculate the parameter $T_{0.01/10}$ (the temperature at which 1% mass loss occurs over 10 hours of isothermal heating) for $[C_8C_1Im][NTf_2]_{0.27}Li[NTf_2]$.

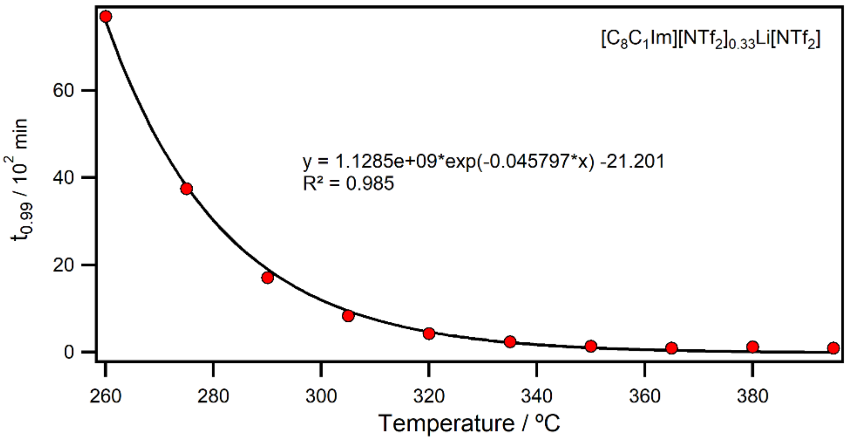

Figure S135 Plot of $t_{0.99}$ versus temperature to calculate the parameter $T_{0.01/10}$ (the temperature at which 1% mass loss occurs over 10 hours of isothermal heating) for $[C_8C_1Im][NTf_2]_{0.33}Li[NTf_2]$.

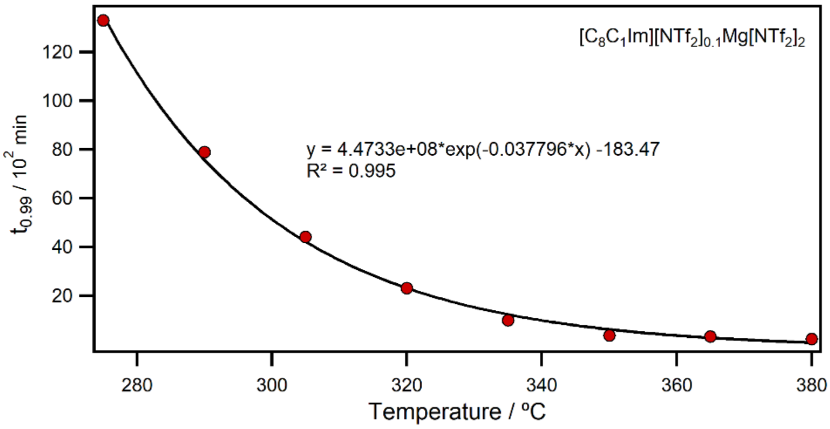

Figure S136 Plot of $t_{0.99}$ versus temperature to calculate the parameter $T_{0.01/10}$ (the temperature at which 1% mass loss occurs over 10 hours of isothermal heating) for $[C_8C_1Im][NTf_2]_{0.1}Mg[NTf_2]_2$.

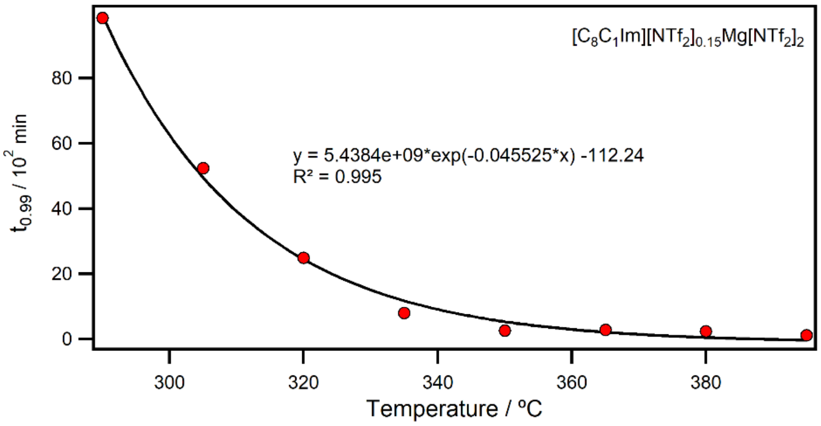

Figure S137 Plot of $t_{0.99}$ versus temperature to calculate the parameter $T_{0.01/10}$ (the temperature at which 1% mass loss occurs over 10 hours of isothermal heating) for $[C_8C_1Im][NTf_2]_{0.15}Mg[NTf_2]_2$.

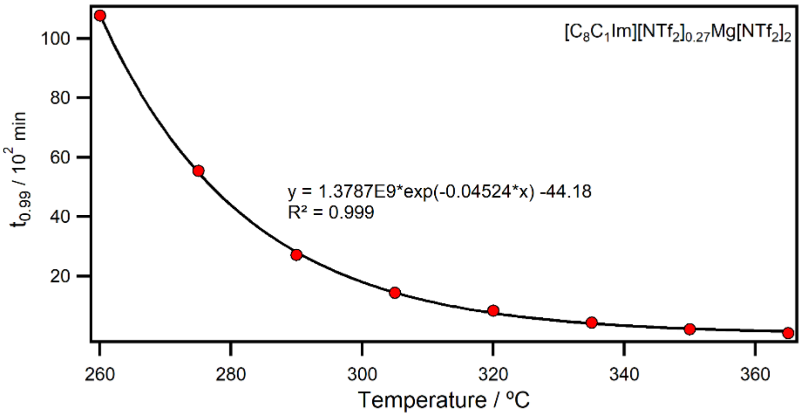

Figure S138 Plot of $t_{0.99}$ versus temperature to calculate the parameter $T_{0.01/10}$ (the temperature at which 1% mass loss occurs over 10 hours of isothermal heating) for $[C_8C_1Im][NTf_2]_{0.27}Mg[NTf_2]_2$.

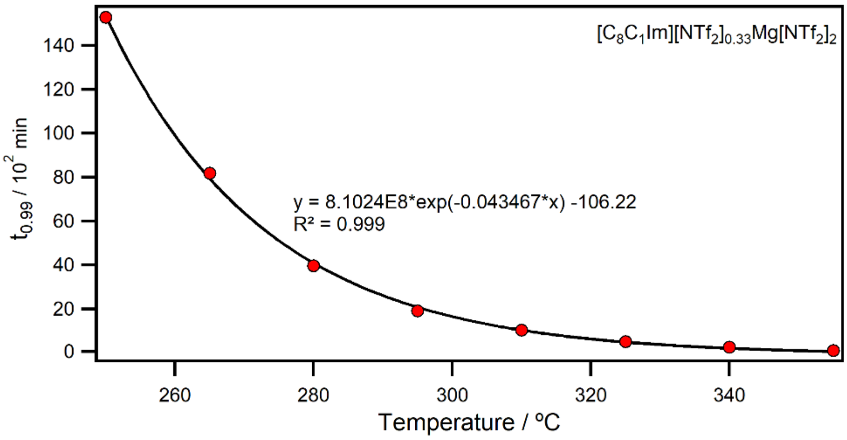

Figure S139 Plot of $t_{0.99}$ versus temperature to calculate the parameter $T_{0.01/10}$ (the temperature at which 1% mass loss occurs over 10 hours of isothermal heating) for $[C_8C_1Im][NTf_2]_{0.33}Mg[NTf_2]_2$.

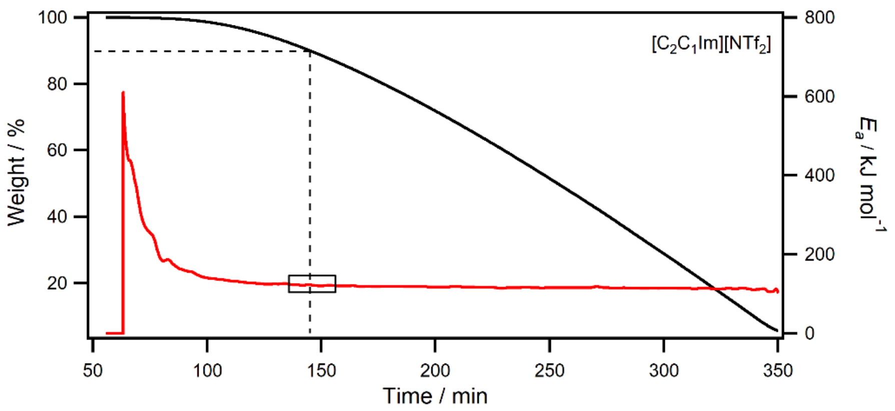

Figure S140 MTGA data for [C_2C_1Im][NTf_2] under Hi-Res ramping (sensitivity = 1, resolution = 6) at 2 °C min^{-1} with a modulation temperature amplitude of 5 °C and a period of 200s. Activation energies taken at 10 wt% mass loss over a 20 minute window to ensure a minimum of 5 modulation periods.

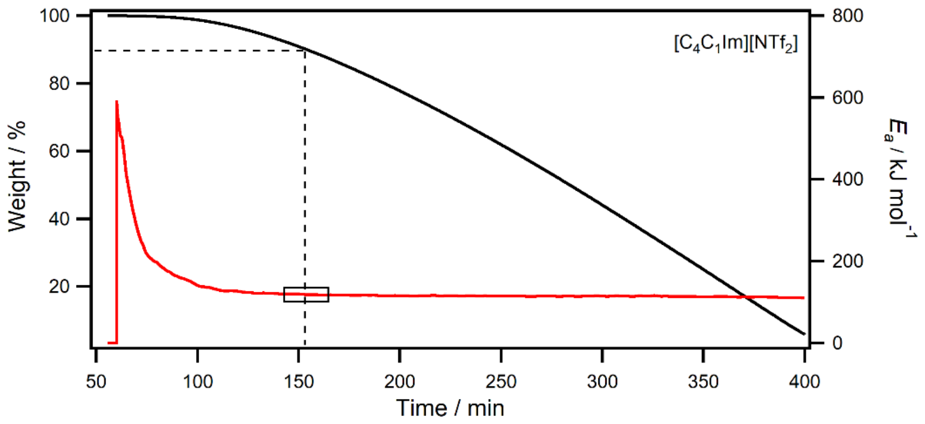

Figure S141 MTGA data for [C_4C_1Im][NTf_2] under Hi-Res ramping (sensitivity = 1, resolution = 6) at 2 °C min^{-1} with a modulation temperature amplitude of 5 °C and a period of 200s. Activation energies taken at 10 wt% mass loss over a 20 minute window to ensure a minimum of 5 modulation periods.

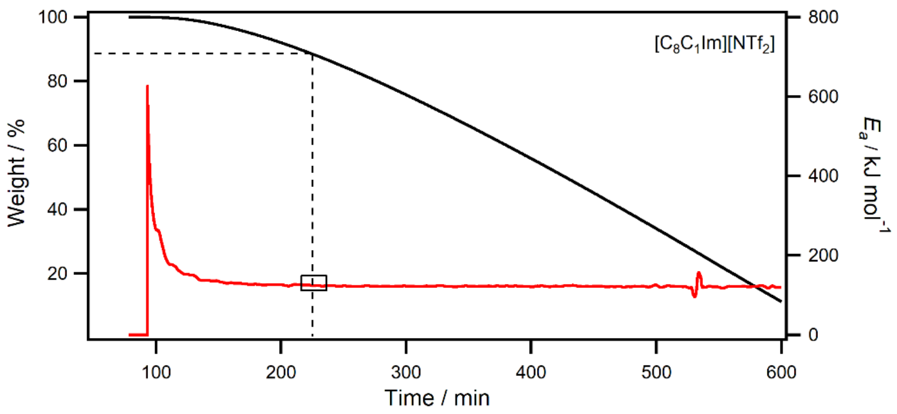

Figure S142 MTGA data for [C_8C_1Im][NTf_2] under Hi-Res ramping (sensitivity = 1, resolution = 6) at 2 °C min^{-1} with a modulation temperature amplitude of 5 °C and a period of 200s. Activation energies taken at 10 wt% mass loss over a 20 minute window to ensure a minimum of 5 modulation periods.

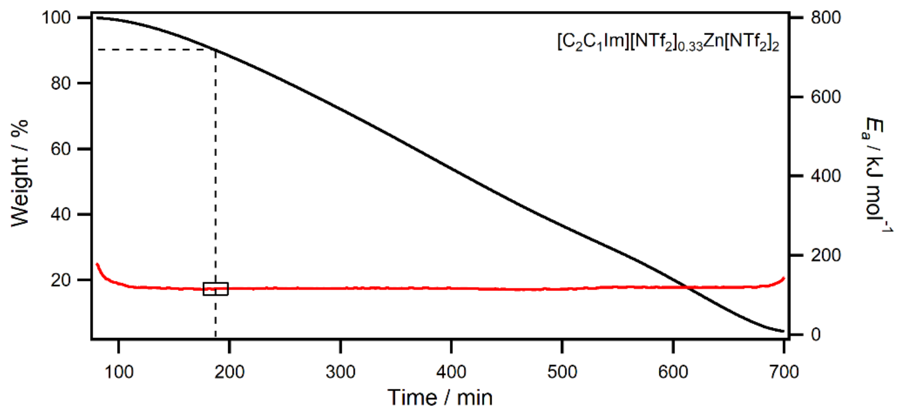

Figure S143 MTGA data for $[C_2C_1Im][NTf_2]_{0.33}Zn[NTf_2]_2$ under Hi-Res ramping (sensitivity = 1, resolution = 6) at 2 °C min^{-1} with a modulation temperature amplitude of 5 °C and a period of 200s. Activation energies taken at 10 wt% mass loss over a 20 minute window to ensure a minimum of 5 modulation periods.

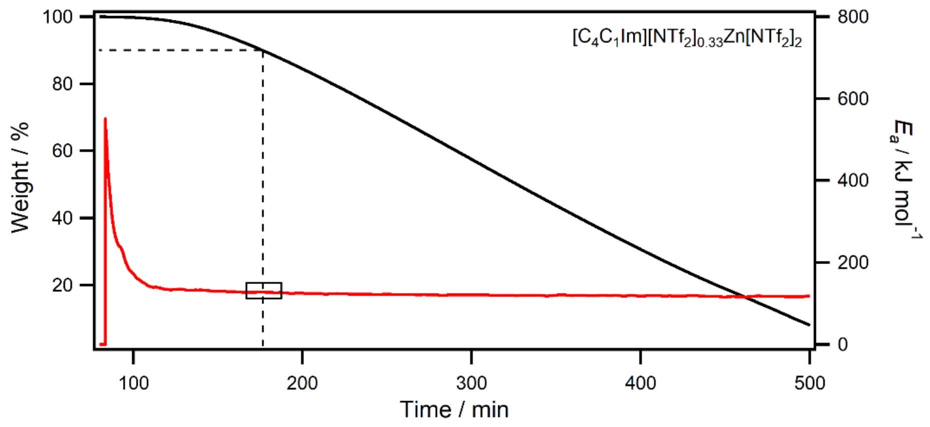

Figure S144 MTGA data for $[C_4C_1Im][NTf_2]_{0.33}Zn[NTf_2]_2$ under Hi-Res ramping (sensitivity = 1, resolution = 6) at 2 °C min^{-1} with a modulation temperature amplitude of 5 °C and a period of 200s. Activation energies taken at 10 wt% mass loss over a 20 minute window to ensure a minimum of 5 modulation periods.

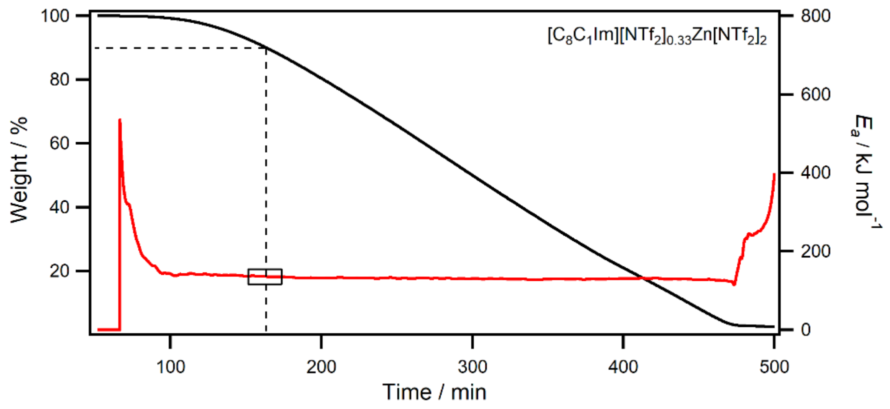

Figure S145 MTGA data for $[C_8C_1Im][NTf_2]_{0.33}Zn[NTf_2]_2$ under Hi-Res ramping (sensitivity = 1, resolution = 6) at 2 °C min^{-1} with a modulation temperature amplitude of 5 °C and a period of 200s. Activation energies taken at 10 wt% mass loss over a 20 minute window to ensure a minimum of 5 modulation periods.

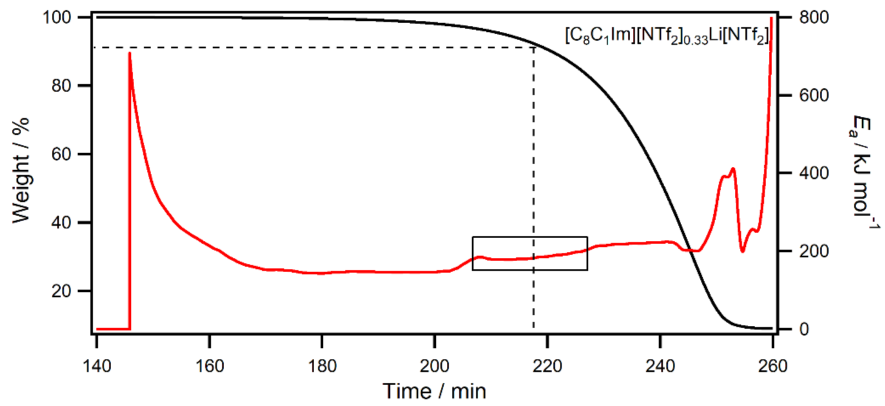

Figure S146 MTGA data for $[C_8C_1Im][NTf_2]_{0.33}Li[NTf_2]$ under Hi-Res ramping (sensitivity = 1, resolution = 6) at 2 °C min^{-1} with a modulation temperature amplitude of 5 °C and a period of 200s. Activation energies taken at 10 wt% mass loss over a 20 minute window to ensure a minimum of 5 modulation periods.

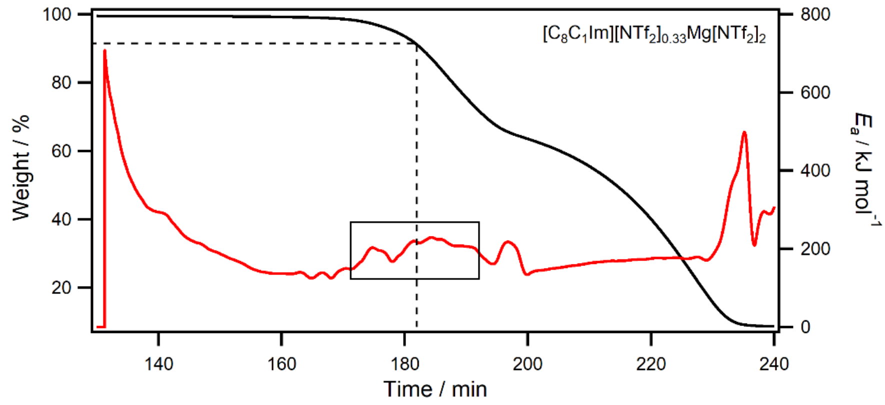

Figure S147 MTGA data for $[C_8C_1Im][NTf_2]_{0.33}Mg[NTf_2]_2$ under Hi-Res ramping (sensitivity = 1, resolution = 6) at 2 °C min^{-1} with a modulation temperature amplitude of 5 °C and a period of 200s. Activation energies taken at 10 wt% mass loss over a 20 minute window to ensure a minimum of 5 modulation periods.

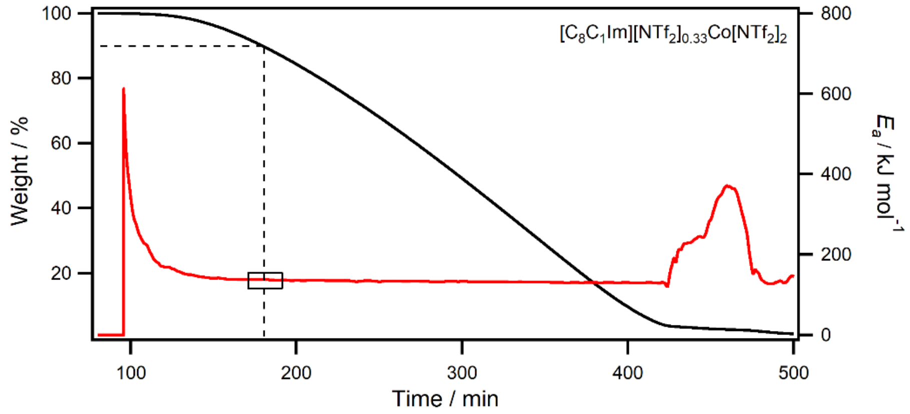

Figure S148 MTGA data for $[C_8C_1Im][NTf_2]_{0.33}Co[NTf_2]_2$ under Hi-Res ramping (sensitivity = 1, resolution = 6) at 2 °C min^{-1} with a modulation temperature amplitude of 5 °C and a period of 200s. Activation energies taken at 10 wt% mass loss over a 20 minute window to ensure a minimum of 5 modulation periods.

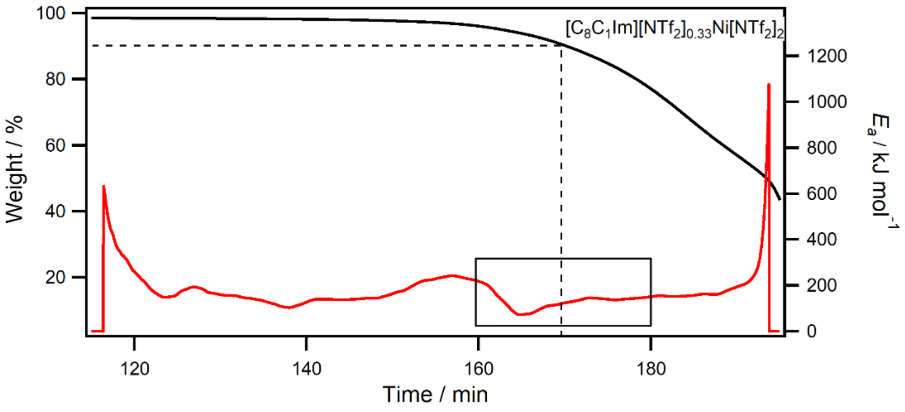

Figure S149 MTGA data for $[C_8C_1Im][NTf_2]_{0.33}Ni[NTf_2]_2$ under Hi-Res ramping (sensitivity = 1, resolution = 6) at 2 °C min^{-1} with a modulation temperature amplitude of 5 °C and a period of 200s. Activation energies taken at 10 wt% mass loss over a 20 minute window to ensure a minimum of 5 modulation periods.

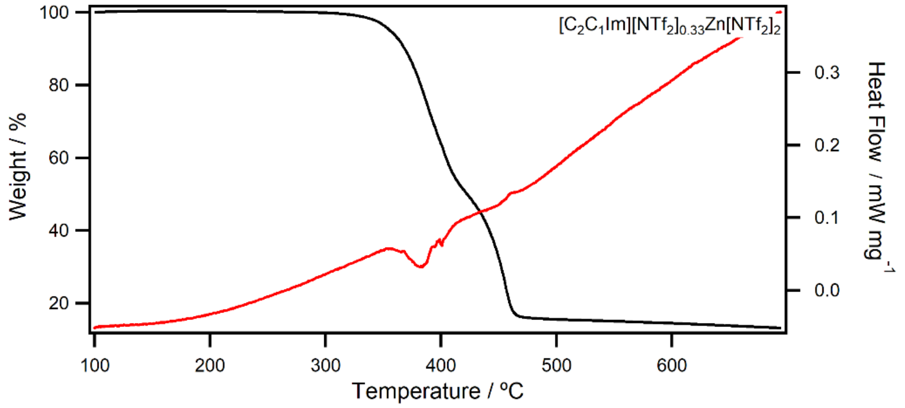

Figure S150 STA data showing mass loss (left axis) and corresponding DSC signals (right axis) for $[C_2C_1Im][NTf_2]_{0.33}Zn[NTf_2]_2$ in 50 mL min^{-1} N_2 at 10 °C min^{-1}.

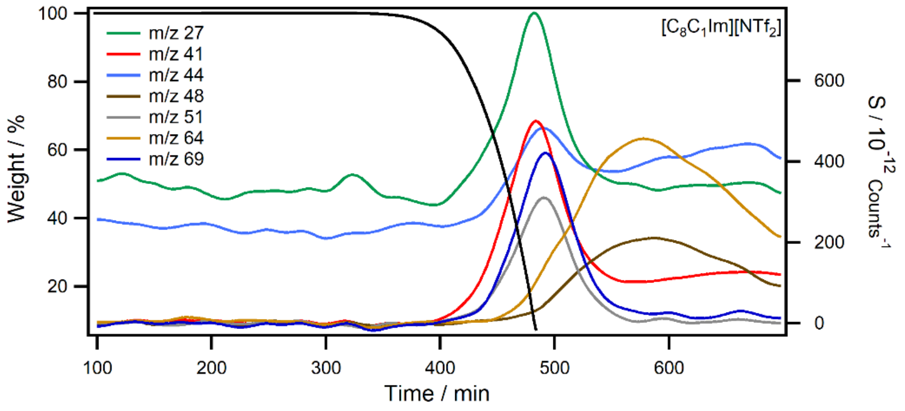

Figure S151 TGA-MS data showing mass loss (left axis) and corresponding MS signals (right axis) for $[C_8C_1Im][NTf_2]$ in 50 mL min^{-1} N_2 at 10 °C min^{-1}.

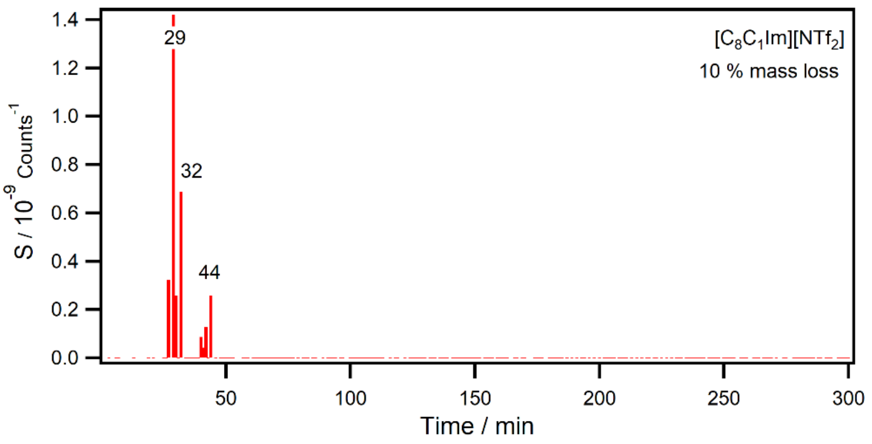

Figure S152 EI-MS mass spectrum from TGA-MS experiments of $[C_8C_1Im][NTf_2]$ in 50 mL min^{-1} N_2 at 10 °C min^{-1} at 10% mass loss.

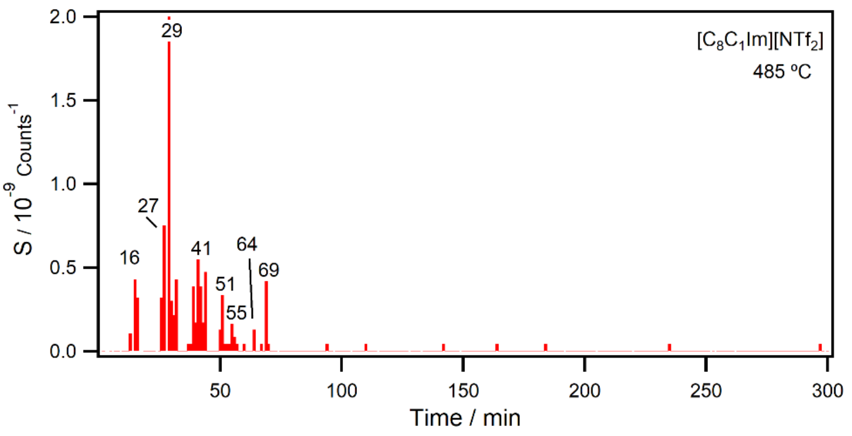

Figure S153 EI-MS mass spectrum from TGA-MS experiments of $[C_8C_1Im][NTf_2]$ in 50 mL min^{-1} N_2 at 10 °C min^{-1} at 485 °C.

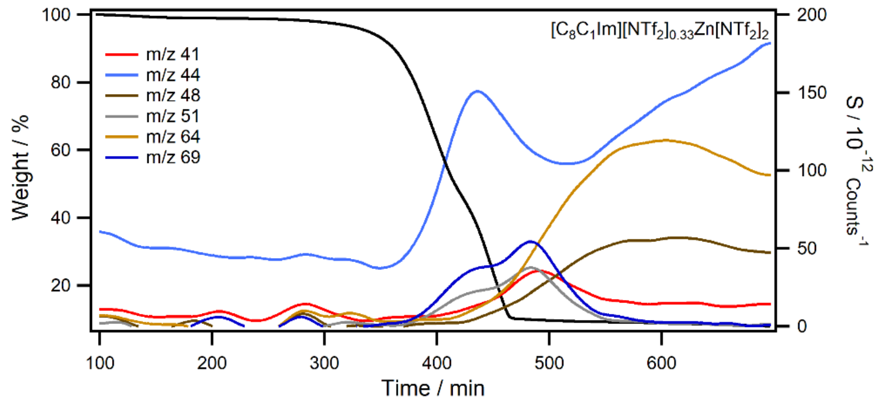

Figure S154 TGA-MS data showing mass loss (left axis) and corresponding MS signals (right axis) for $[C_8C_1Im][NTf_2]_{0.33}Zn[NTf_2]_2$ in 50 mL min^{-1} N_2 at 10 °C min^{-1}.

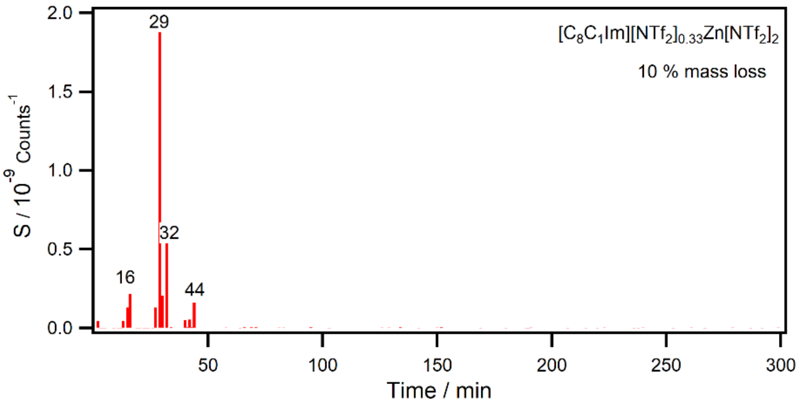

Figure S155 EI-MS mass spectrum from TGA-MS experiments of $[C_8C_1Im][NTf_2]_{0.33}Zn[NTf_2]_2$ in 50 mL min^{-1} N_2 at 10 °C min^{-1} at 10% mass loss.

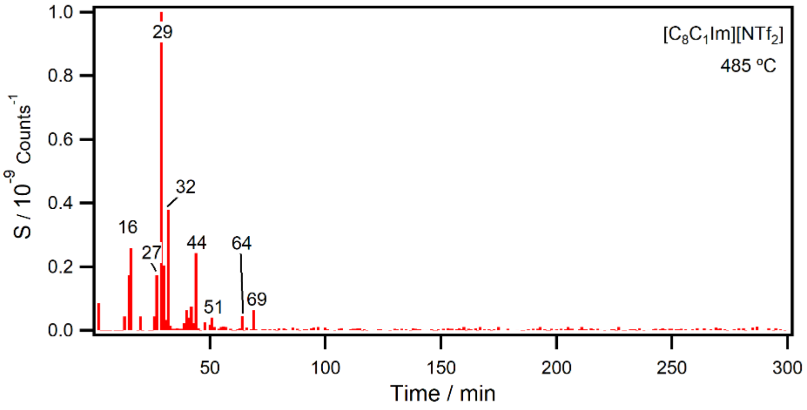

Figure S156 EI-MS mass spectrum from TGA-MS experiments of $[C_8C_1Im][NTf_2]_{0.33}Zn[NTf_2]_2$ in 50 mL min^{-1} N_2 at 10 °C min^{-1} at 485 °C.

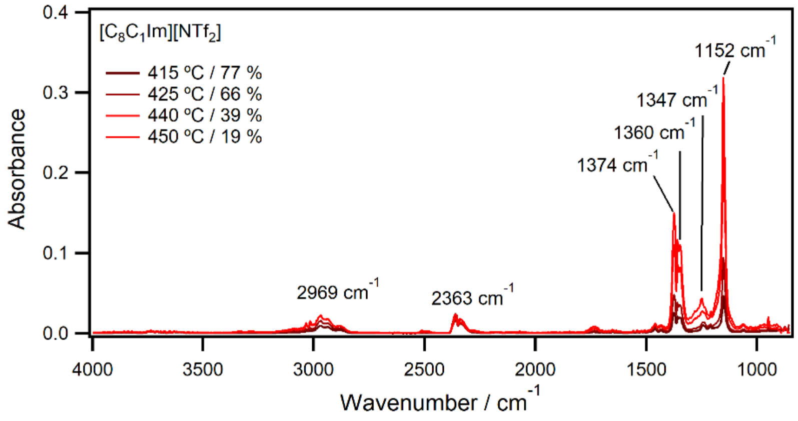

Figure S157 TGA-IR IR spectra for $[C_8C_1Im][NTf_2]$ in 50 mL min^{-1} N_2 at 10 °C min^{-1}.

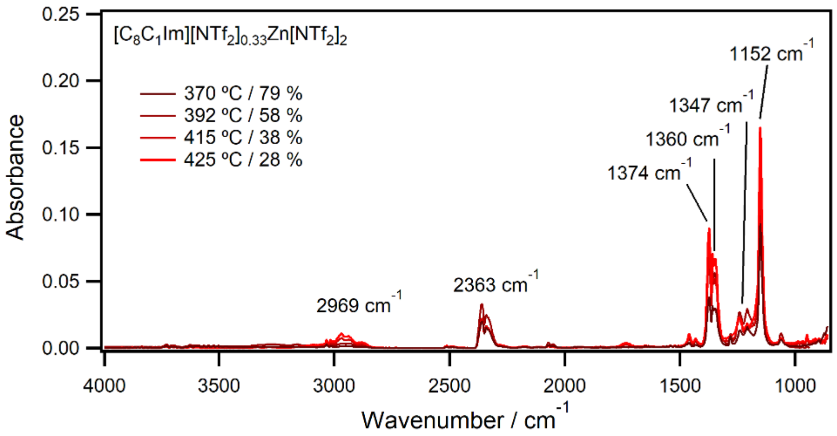

Figure S158 TGA-IR IR spectra for $[C_8C_1Im][NTf_2]_{0.33}Zn[NTf_2]_2$ in 50 mL min^{-1} N_2 at 10 °C min^{-1}.

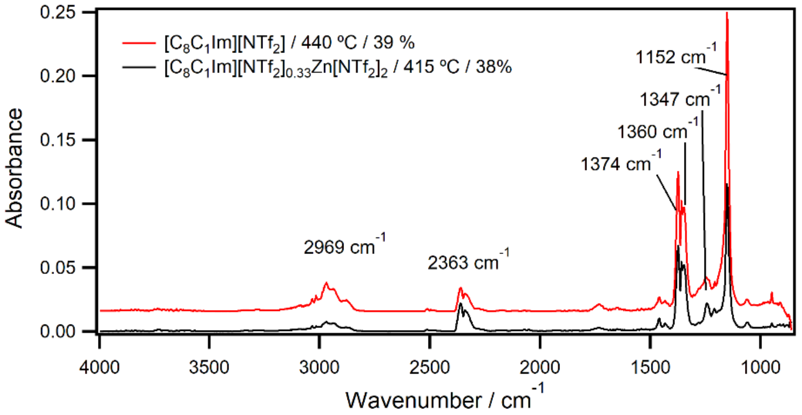

Figure S159 Comparison of TGA-IR IR spectra for $[C_8C_1Im][NTf_2]$ (red) and $[C_8C_1Im][NTf_2]_{0.33}Zn[NTf_2]_2$ (black) at approximately 40 % remaining mass in 50 mL min^{-1} N_2 at 10 °C min^{-1}.

Thermal Desorption Data

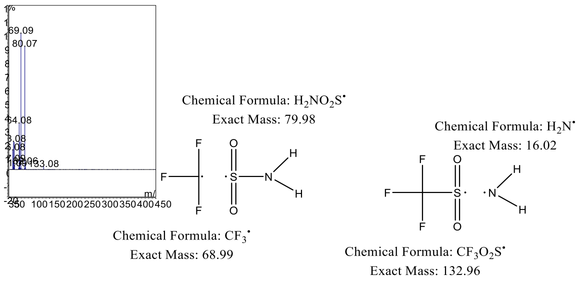

Figure S160 Mass spectrum (EI) from TD-GC-MS experiments at 14.53 minutes retention time with peak assignments.

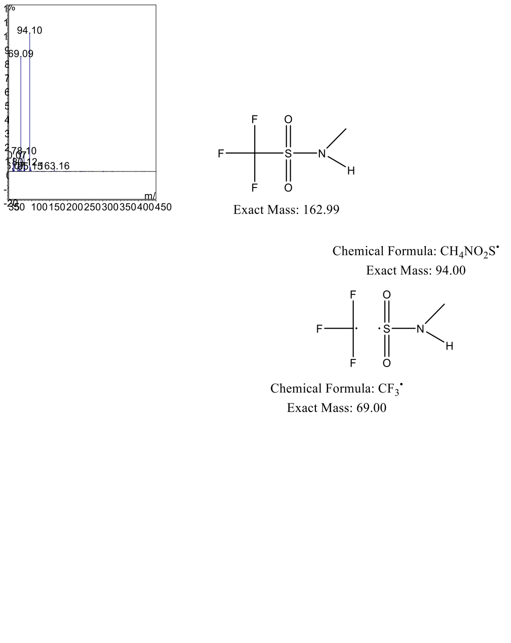

Figure S161 Mass spectrum (EI) from TD-GC-MS experiments at 13.23 minutes retention time with peak assignments.

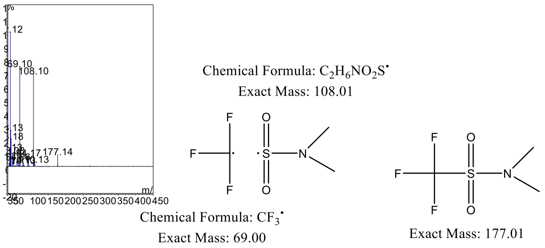

Figure S163 Mass spectrum (EI) from TD-GC-MS experiments at 10.52 minutes retention time with peak assignments.

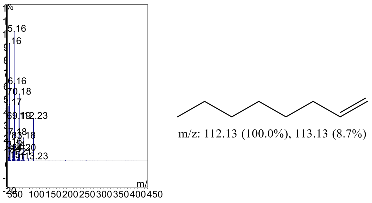

Figure S162 Mass spectrum (EI) from TD-GC-MS experiments at 10.00 minutes retention time with peak assignments.

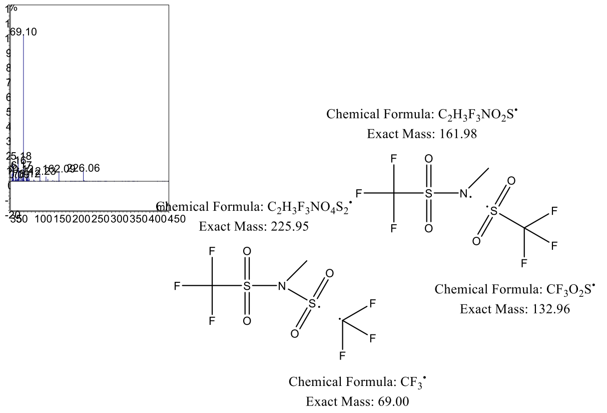

Figure S165 Mass spectrum (EI) from TD-GC-MS experiments at 9.78 minutes retention time with peak assignments.

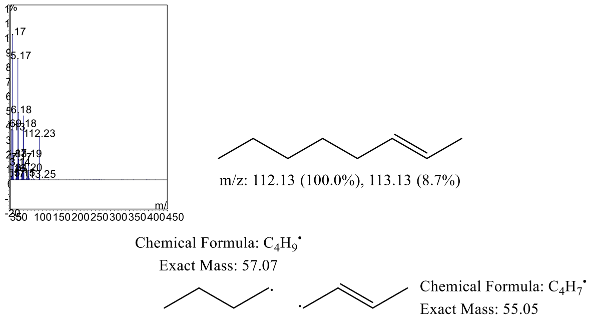

Figure S164 Mass spectrum (EI) from TD-GC-MS experiments at 9.65 minutes retention time with peak assignments.

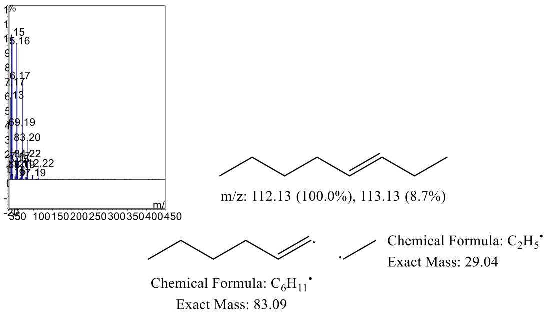

Figure S167 Mass spectrum (EI) from TD-GC-MS experiments at 9.65 minutes retention time with peak assignments.

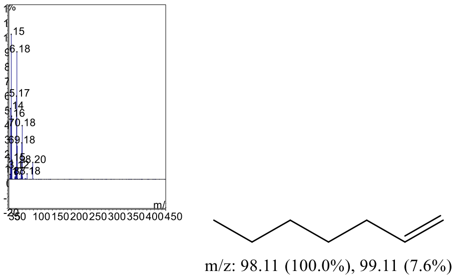

Figure S166 Mass spectrum (EI) from TD-GC-MS experiments at 7.80 minutes retention time with peak assignments.

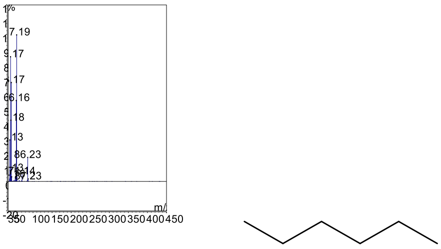

m/z: 86.12 (100.0%), 87.11 (6.5%)

Figure S169 Mass spectrum (EI) from TD-GC-MS experiments at 6.02 minutes retention time with peak assignments.

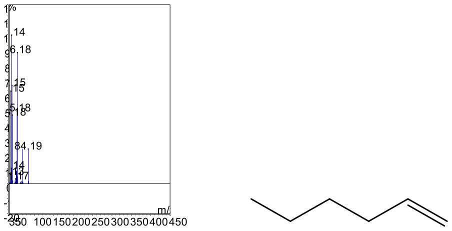

m/z: 84.09 (100.0%), 85.10 (6.5%)

Figure S168 Mass spectrum (EI) from TD-GC-MS experiments at 5.95 minutes retention time with peak assignments.

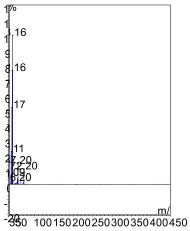

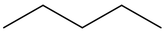

m/z: 72.09 (100.0%), 73.10 (5.4%)

Figure S171 Mass spectrum (EI) from TD-GC-MS experiments at 4.34 minutes retention time with peak assignments.

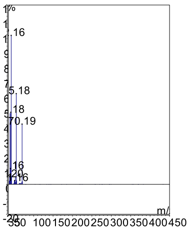

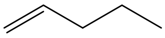

m/z: 70.08 (100.0%), 71.08 (5.4%)

Figure S170 Mass spectrum (EI) from TD-GC-MS experiments at 4.32 minutes retention time with peak assignments.

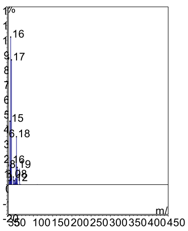

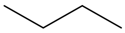

m/z: 58.08 (100.0%), 59.08 (4.3%)

Figure S173 Mass spectrum (EI) from TD-GC-MS experiments at 3.30 minutes retention time with peak assignments.

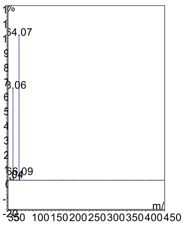

m/z: 63.96 (100.0%), 65.96 (4.5%)

Chemical Formula: OS
Exact Mass: 47.97

Chemical Formula: $O^{2\bullet}$
Exact Mass: 15.99

Figure S172 Mass spectrum (EI) from TD-GC-MS experiments at 3.10 minutes retention time with peak assignments.

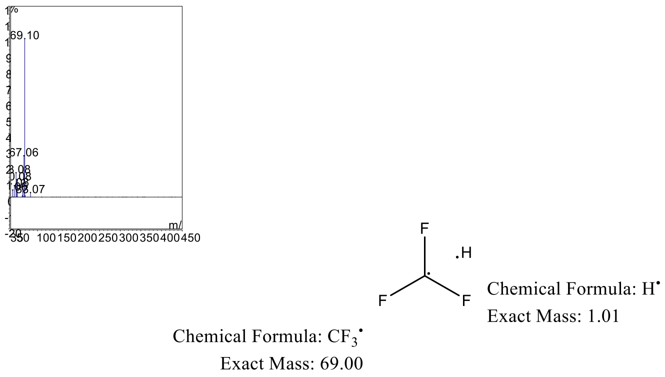

Figure S174 Mass spectrum (EI) from TD-GC-MS experiments at 2.65 minutes retention time with peak assignments.

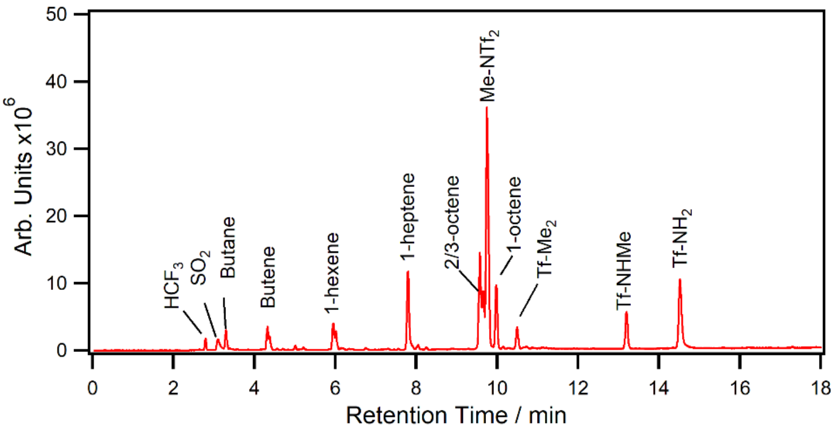

Figure S175 Gas chromatogram from TD-GC-MS of $[C_8C_1Im][NTf_2]_{0.33}Li[NTf_2]$.

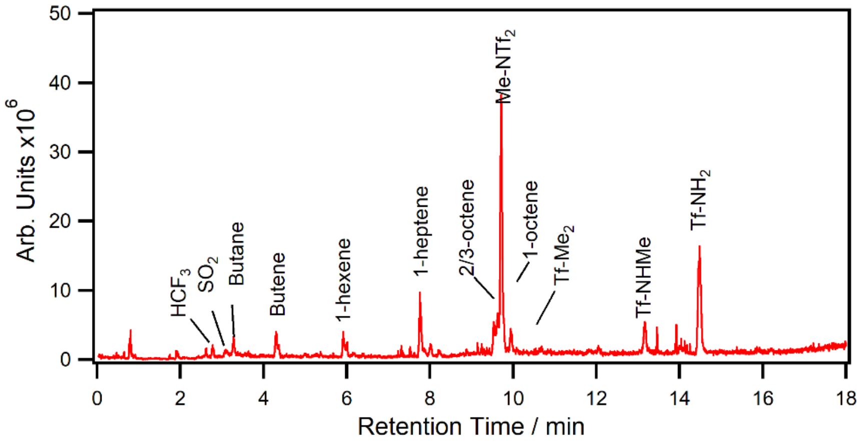

Figure S176 Gas chromatogram from TD-GC-MS of $[C_8C_1Im][NTf_2]_{0.33}Mg[NTf_2]_2$.

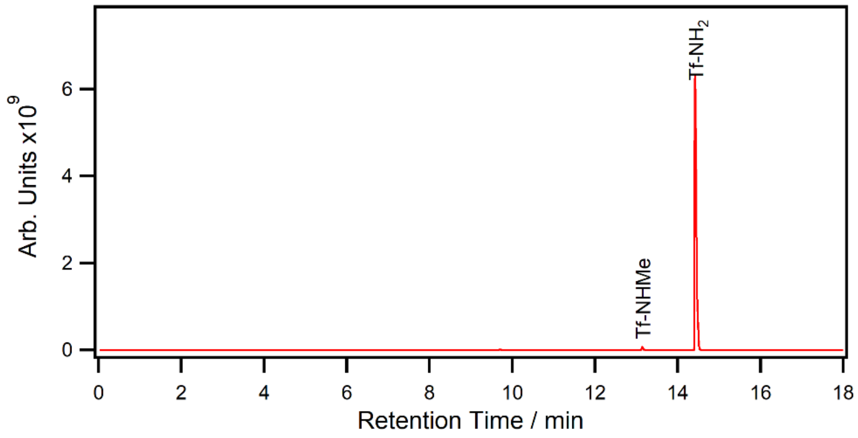

Figure S177 Gas chromatogram from TD-GC-MS of $[C_8C_1Im][NTf_2]_{0.33}Co[NTf_2]_2$ of 0-18 minute region.

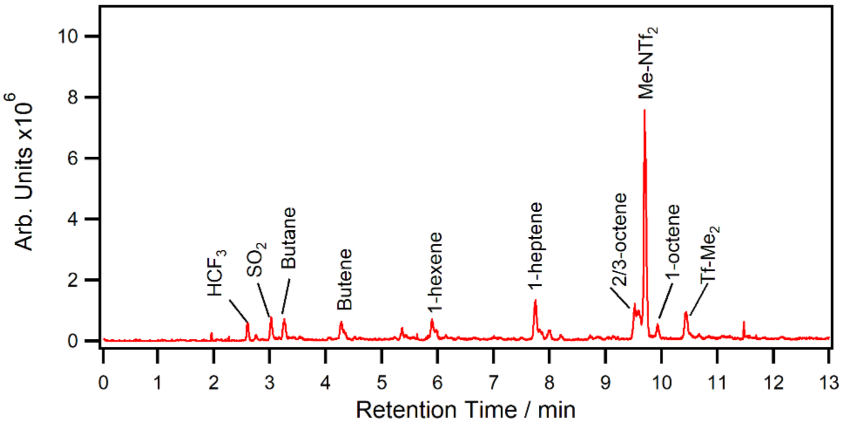

Figure S178 Gas chromatogram from TD-GC-MS of $[C_8C_1Im][NTf_2]_{0.33}Co[NTf_2]_2$ of 0-13 minute region.

ESI-MS Decomposition Data

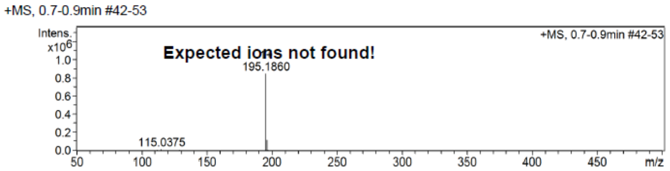

#	m/z	I %
1	90.0558	0.2
2	90.9772	0.4
3	98.0219	0.7
4	104.0713	0.6
5	107.0426	0.3
6	107.9686	0.2
7	112.0381	0.8
8	115.0375	1.8
9	116.0701	0.3
10	118.0860	0.4
11	129.0527	0.9
12	142.0490	0.3
13	145.0484	0.5
14	146.0808	0.2
15	158.9650	0.3
16	159.0639	0.4
17	195.1860	100.0
18	196.1887	13.7
19	197.1918	0.9
20	325.8974	0.4

Figure S179 Positive ESI-MS of unheated $[C_8C_1Im][NTf_2]$.

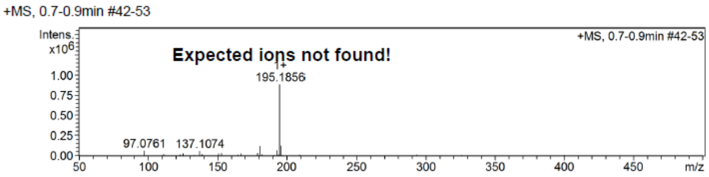

#	m/z	I %
1	97.0761	7.5
2	111.0922	2.4
3	123.0920	1.6
4	125.1075	3.0
5	137.1074	7.4
6	139.1229	2.1
7	151.1230	3.0
8	153.1385	3.4
9	165.1385	1.7
10	167.1540	3.0
11	179.1541	3.7
12	181.1698	13.5
13	182.1730	1.8
14	191.1538	1.3
15	193.1698	8.1
16	194.1742	1.3
17	195.1856	100.0
18	196.1886	14.4
19	209.1948	1.5
20	293.2943	1.4

Figure S180 Positive ESI-MS from the residue of a TGA pan containing $[C_8C_1Im][NTf_2]$ which was heated under 10 mL min^{-1} N_2 until 40% of the mass had decomposed.

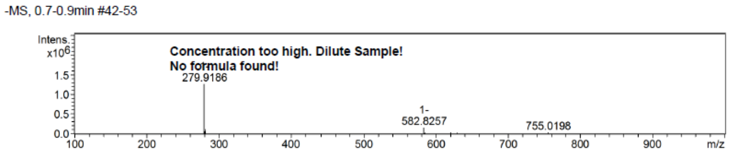

#	m/z	I %
1	279.9186	100.0
2	280.0890	0.4
3	280.7527	0.4
4	280.9196	4.5
5	281.9147	10.3
6	282.9167	0.4
7	582.8257	12.8
8	583.8260	1.1
9	584.8223	2.5
10	619.9169	3.0
11	620.9185	0.4
12	621.9122	0.6
13	628.8803	2.4
14	630.8772	0.5
15	642.8963	0.8
16	656.9112	1.0
17	741.0036	0.7
18	755.0198	3.1
19	756.0221	0.7
20	757.0177	0.7

Figure S181 Negative ESI-MS from the residue of a TGA pan containing $[C_8C_1Im][NTf_2]$ which was heated under 10 mL min^{-1} N_2 until 40% of the mass had decomposed.

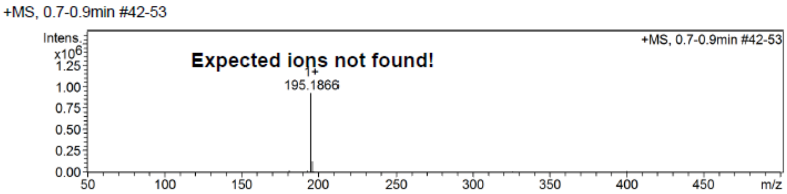

#	m/z	I %
1	112.0379	0.2
2	115.0378	0.5
3	120.9877	0.3
4	129.0531	0.2
5	137.1076	0.2
6	151.1237	0.2
7	153.1392	0.2
8	158.9650	0.3
9	181.1704	2.0
10	182.1728	0.3
11	185.1149	0.3
12	193.1705	2.0
13	194.1784	0.5
14	195.1866	100.0
15	196.1893	13.7
16	197.1925	0.9
17	226.9520	0.3
18	256.9629	0.2
19	325.8962	0.8
20	400.0785	1.5

Figure S182 Positive mode ESI-MS from the residue of a TGA pan containing $[C_8C_1Im][NTf_2]_{0.33}Zn[NTf_2]_2$ which was heated under 10 mL min^{-1} N_2 until 40% of the mass had decomposed.

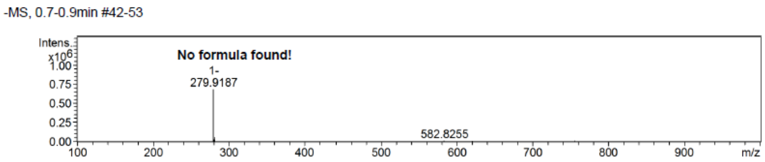

#	m/z	I %
1	278.4723	0.1
2	278.5115	0.1
3	279.9187	100.0
4	280.0895	0.4
5	280.2079	0.2
6	280.7452	0.2
7	280.9199	4.4
8	281.9151	9.6
9	282.9177	0.4
10	283.9127	0.3
11	582.8255	2.1
12	583.8279	0.2
13	584.8224	0.4
14	619.9165	1.4
15	620.9176	0.2
16	621.9135	0.3
17	633.9290	0.1
18	755.0196	2.0
19	756.0223	0.4
20	757.0173	0.4

Figure S183 Negative mode ESI-MS from the residue of a TGA pan containing $[C_8C_1Im][NTf_2]_{0.33}Zn[NTf_2]_2$ which was heated under 10 mL min^{-1} N_2 until 40% of the mass had decomposed.

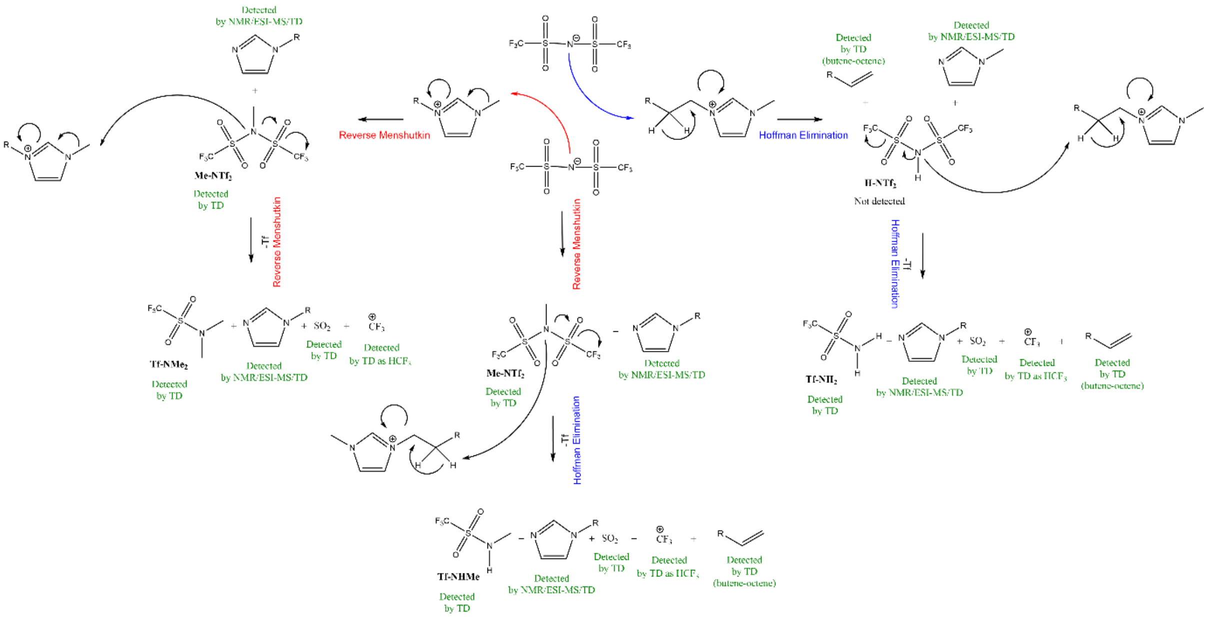

Scheme S1 Mechanism of thermal decomposition for 1-alkyl-3-methylimidazolium bistriflimide ionic liquids. Weakly coordinated metals enhance the production of Tf-NH_2 through successive elimination reactions by either stabilising a transition state or polarising the $[NTf_2]^-$ anion.

Annealing Experiments

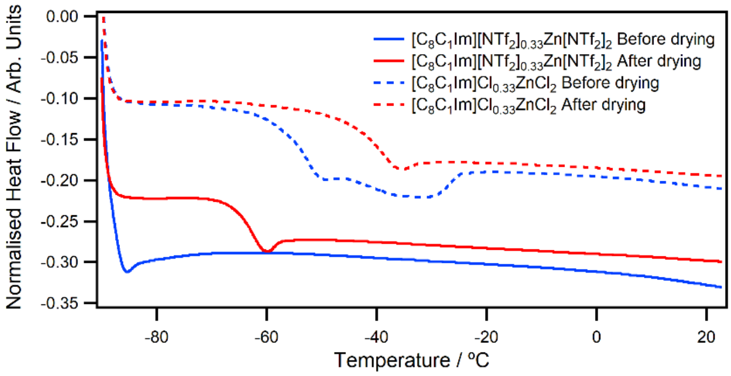

Figure S184 DSC data before and after *in situ* drying of $[C_8C_1Im]X_{0.33}ZnX_2$ samples with 10 wt% water for 45 minutes at 100 °C.

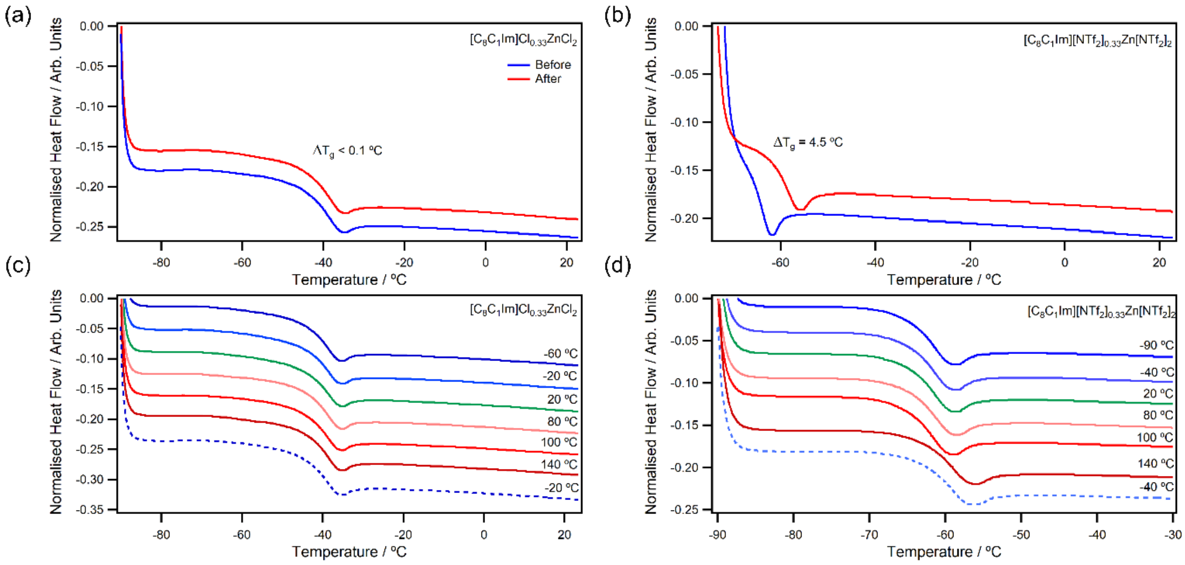

Figure S185 DSC annealing experiments, showing DSC thermograms (a-b) before (blue) and after (red) heating. (c-d) DSC scans between 1 hour annealing isothermal holds.

Viscosity and Density

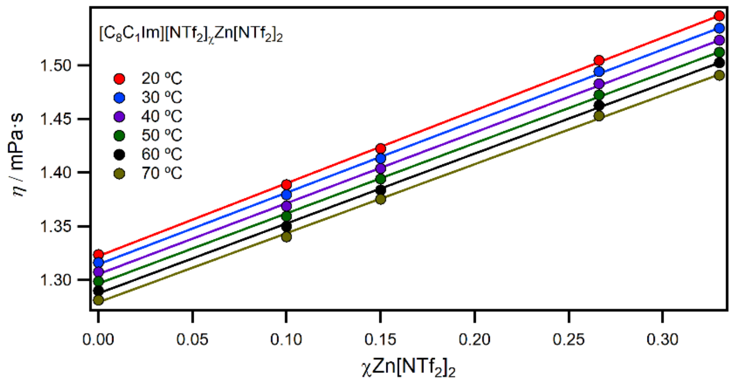

Figure S186 Densities of $[C_8C_1Im][NTf_2]_xZn[NTf_2]_2$ ionic liquids against mole fraction of $Zn[NTf_2]_2$.

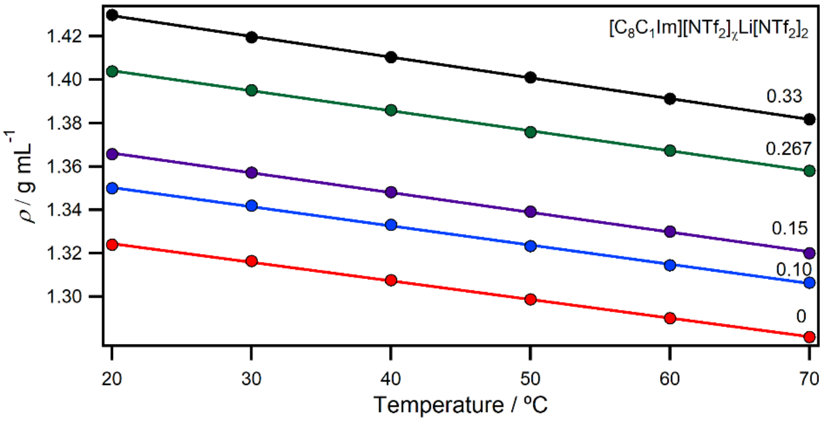

Figure S187 Densities of $[C_8C_1Im][NTf_2]_xLi[NTf_2]_2$ ionic liquids against temperature.

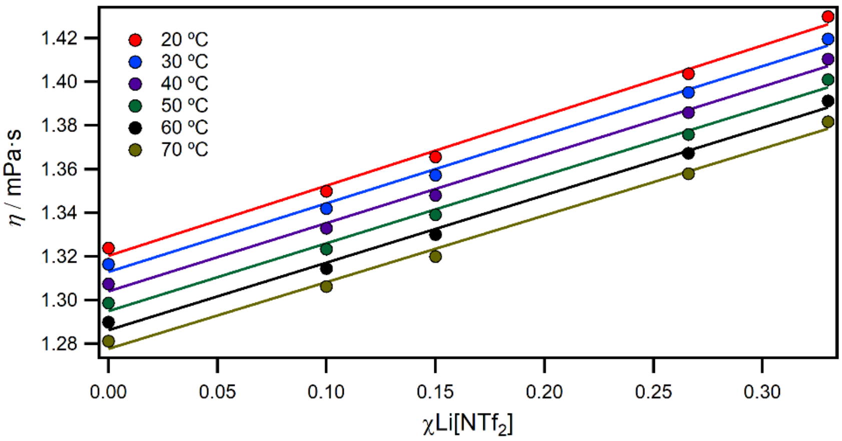

Figure S188 Densities of $[C_8C_1Im][NTf_2]_xLi[NTf_2]$ ionic liquids against mole fraction of $Li[NTf_2]$.

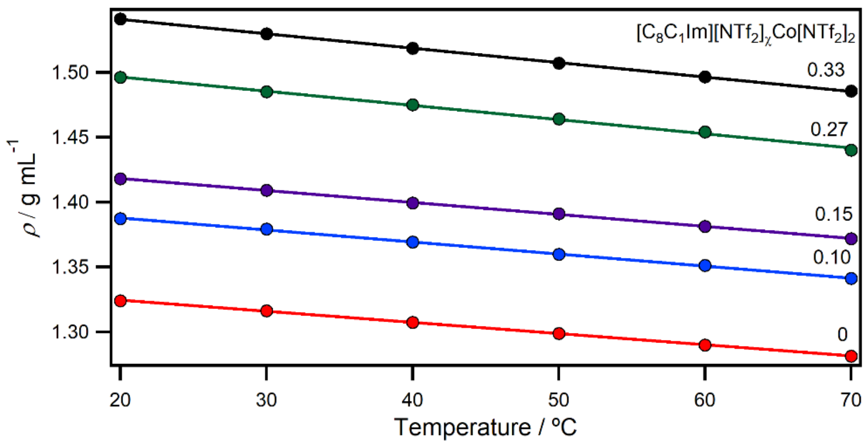

Figure S189 Densities of $[C_8C_1Im][NTf_2]_xCo[NTf_2]_2$ ionic liquids against temperature.

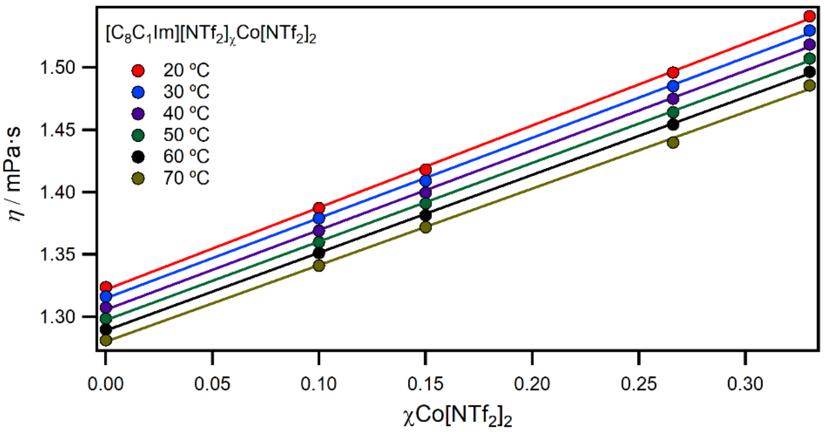

Figure S190 Densities of $[C_8C_1Im][NTf_2]_xCo[NTf_2]_2$ ionic liquids against mole fraction of $Co[NTf_2]_2$.

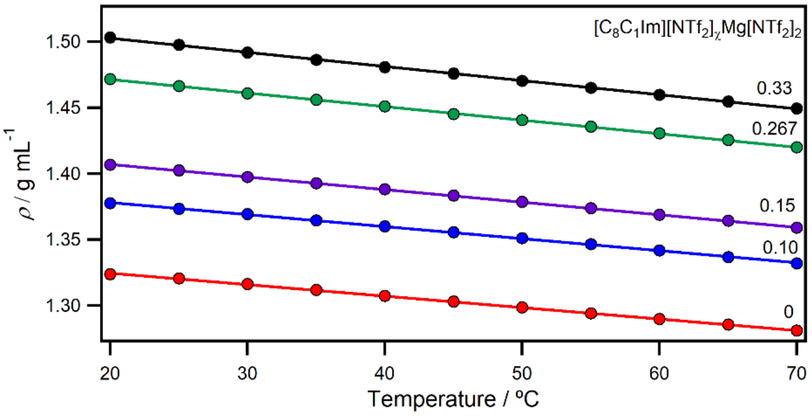

Figure S191 Densities of $[C_8C_1Im][NTf_2]_xMg[NTf_2]_2$ ionic liquids against temperature.

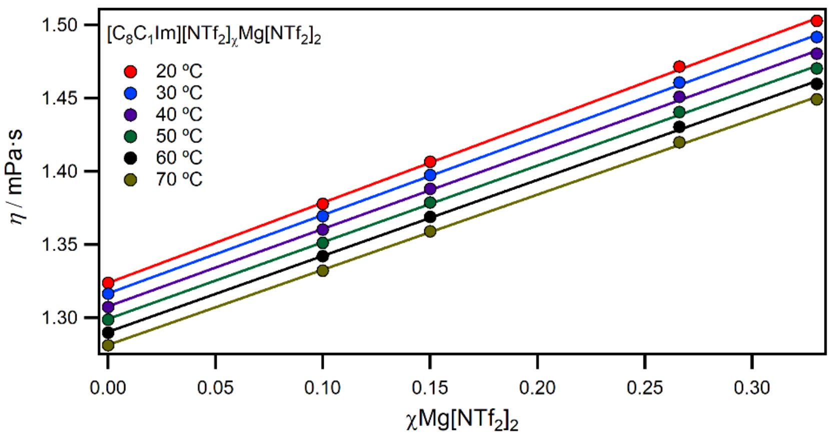

Figure S192 Densities of $[C_8C_1Im][NTf_2]_xMg[NTf_2]_n$ ionic liquids against mole fraction of $Mg[NTf_2]_2$.

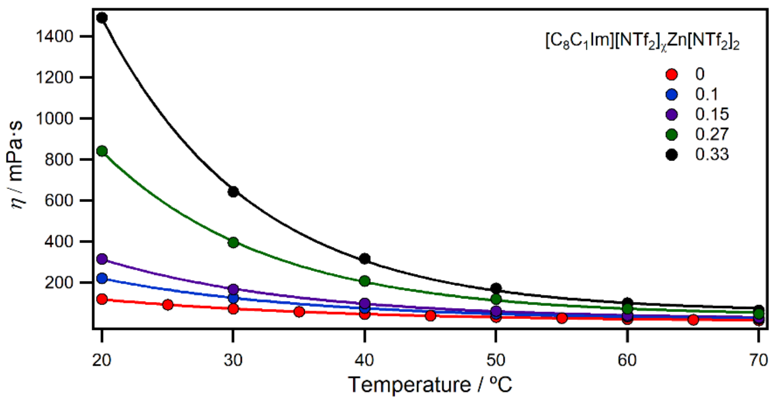

Figure S193 Viscosities of $[C_8C_1Im][NTf_2]_xZn[NTf_2]_2$ ionic liquids against temperature.

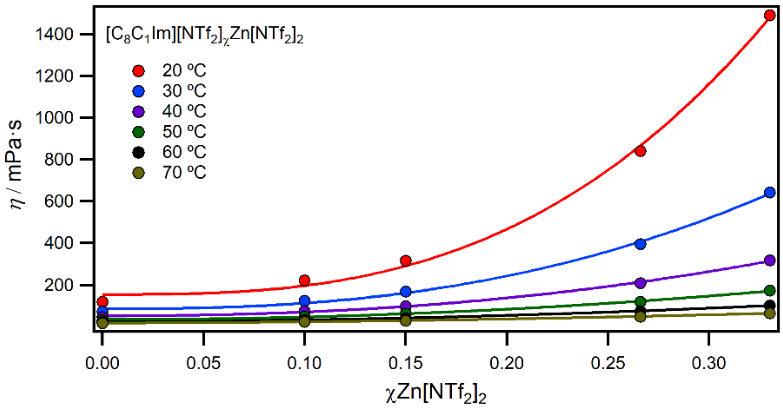

Figure S194 Viscosities of $[C_8C_1Im][NTf_2]_xZn[NTf_2]_2$ ionic liquids against mole fraction of $Zn[NTf_2]_2$.

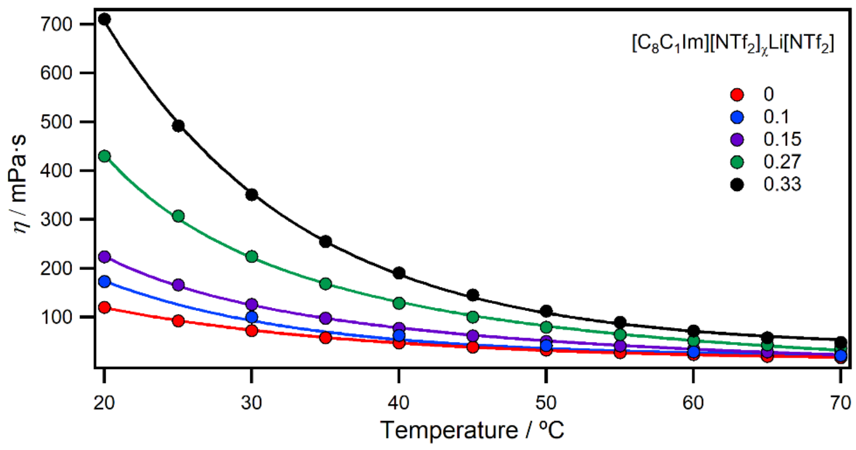

Figure S195 Viscosities of $[C_8C_1Im][NTf_2]_xLi[NTf_2]$ ionic liquids against temperature.

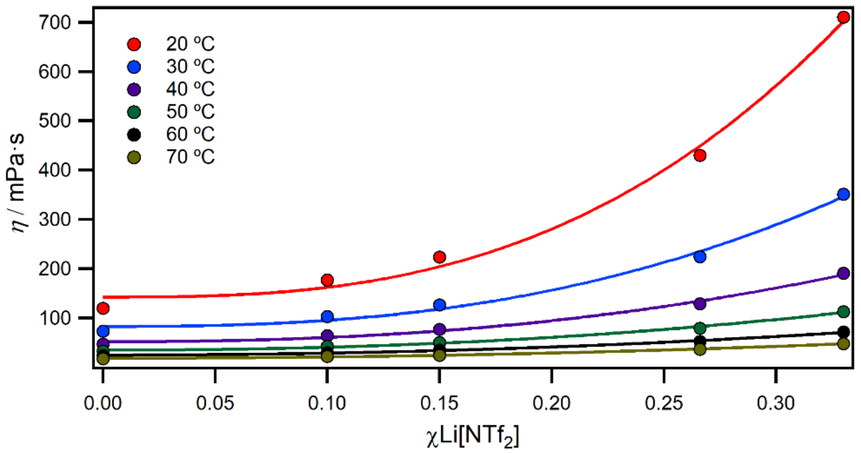

Figure S196 Viscosities of $[C_8C_1Im][NTf_2]_xLi[NTf_2]$ ionic liquids against mole fraction of $Li[NTf_2]$.

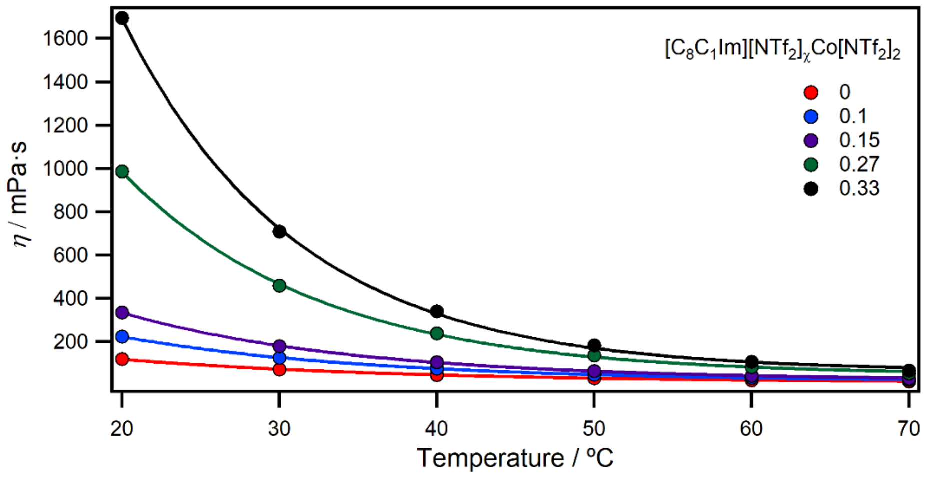

Figure S197 Viscosities of $[C_8C_1Im][NTf_2]_xCo[NTf_2]_2$ ionic liquids against temperature.

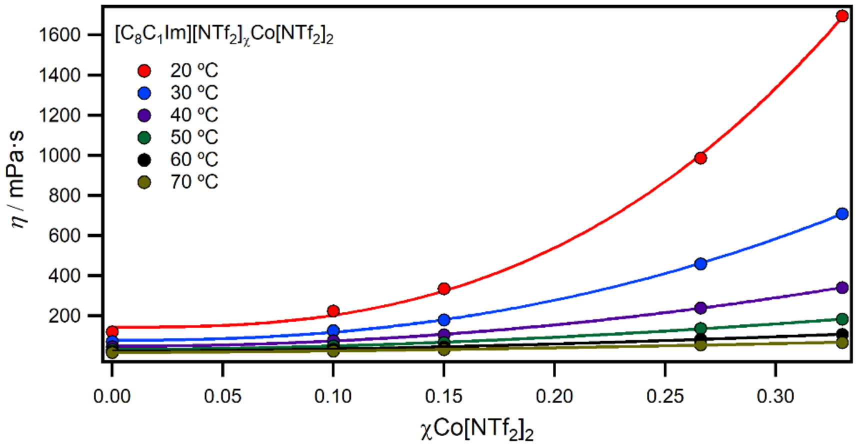

Figure S198 Viscosities of $[C_8C_1Im][NTf_2]_xCo[NTf_2]_2$ ionic liquids against mole fraction of $Co[NTf_2]_2$.

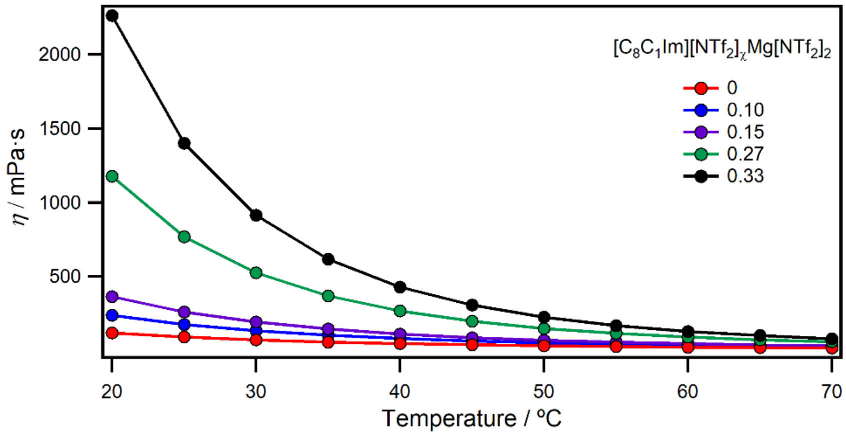

Figure S199 Viscosities of $[C_8C_1Im][NTf_2]_xMg[NTf_2]_2$ ionic liquids against temperature.

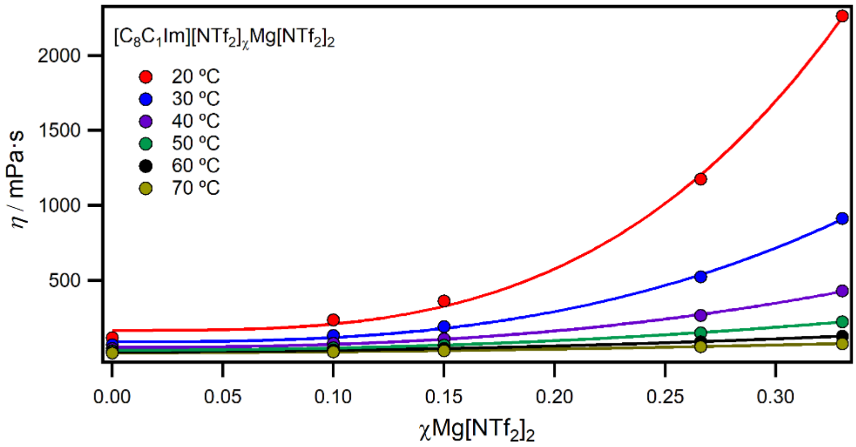

Figure S200 Viscosities of $[C_8C_1Im][NTf_2]_xMg[NTf_2]_2$ ionic liquids against mole fraction of $Mg[NTf_2]_2$.

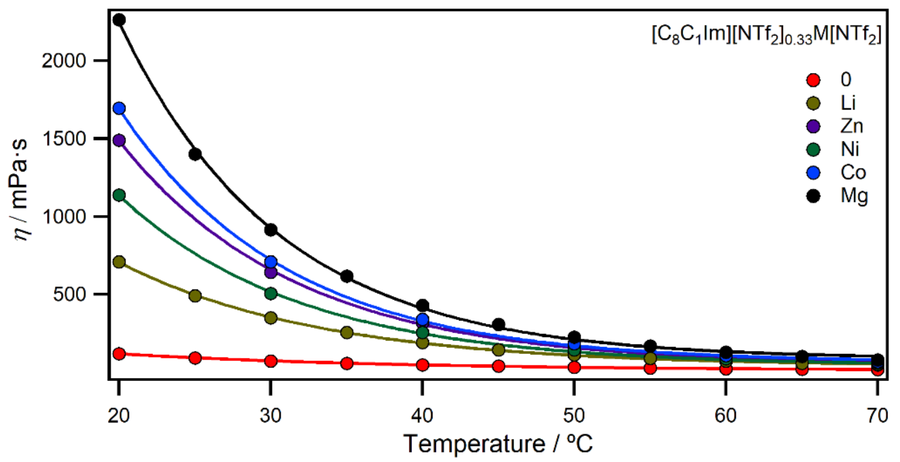

Figure S201 Viscosities of $[C_8C_1Im][NTf_2]_{0.33}M[NTf_2]_2$ ionic liquids against temperature.

Table S2 Viscosities of $[C_8C_1Im][NTf_2]_χZn[NTf_2]_2$ ionic liquids from 20-70°C.

	Viscosity / mPa.s					
χ	20 °C	30 °C	40 °C	50 °C	60 °C	70 °C
0	120.1	72.9	47.3	32.4	23.4	17.3
0.1	221.7	125.1	76.2	49.5	33.9	24.2
0.15	315.4	168.7	99.1	62.5	41.7	29.2
0.27	841.4	395.7	209.0	120.1	74.3	48.9
0.33	1490.6	642.8	317.2	172.5	102.1	64.8

Table S3 Densities of $[C_8C_1Im][NTf_2]_χZn[NTf_2]_2$ ionic liquids from 20-70°C.

	Density / g mL^{-1}					
χ	20 °C	30 °C	40 °C	50 °C	60 °C	70 °C
0	1.32	1.32	1.31	1.30	1.29	1.28
0.1	1.39	1.38	1.37	1.36	1.35	1.34
0.15	1.42	1.41	1.40	1.39	1.38	1.38
0.27	1.50	1.49	1.48	1.47	1.46	1.45
0.33	1.55	1.53	1.52	1.51	1.50	1.49

Table S4 Viscosities of $[C_8C_1Im][NTf_2]_χMg[NTf_2]_2$ ionic liquids from 20-70°C.

	Viscosity / mPa.s					
χ	20 °C	30 °C	40 °C	50 °C	60 °C	70 °C
0	120.1	72.9	47.3	32.4	23.4	17.3
0.1	238.8	134.2	81.4	52.7	35.9	25.6
0.15	364.0	193.1	112.3	70.1	46.4	32.3
0.27	1177.1	525.7	267.8	150.2	90.9	58.6
0.33	2263.8	914.9	430.2	225.7	129.6	80.0

Table S5 Densities of $[C_8C_1Im][NTf_2]_\chi Mg[NTf_2]_2$ ionic liquids from 20-70°C.

	Density / g mL^{-1}					
χ	20 °C	30 °C	40 °C	50 °C	60 °C	70 °C
0	1.32	1.32	1.31	1.30	1.29	1.28
0.1	1.38	1.37	1.36	1.35	1.34	1.33
0.15	1.41	1.40	1.39	1.38	1.37	1.36
0.27	1.47	1.46	1.45	1.44	1.43	1.42
0.33	1.50	1.49	1.48	1.47	1.46	1.45

Table S6 Viscosities of $[C_8C_1Im][NTf_2]_\chi Li[NTf_2]$ ionic liquids from 20-70°C.

	Viscosity / mPa.s					
χ	20 °C	30 °C	40 °C	50 °C	60 °C	70 °C
0	120.1	72.9	47.3	32.4	23.4	17.3
0.1	172.7	100.2	41.8	41.8	29.3	21.3
0.15	223.4	126.3	77.3	50.4	34.5	24.2
0.27	429.9	224.8	128.9	79.5	52.2	36.1
0.33	710.6	351.0	190.5	112.8	71.5	48.0

Table S7 Densities of $[C_8C_1Im][NTf_2]_\chi Li[NTf_2]$ ionic liquids from 20-70°C.

	Density / g mL^{-1}					
χ	20 °C	30 °C	40 °C	50 °C	60 °C	70 °C
0	1.32	1.32	1.31	1.30	1.29	1.28
0.1	1.35	1.34	1.33	1.32	1.31	1.31
0.15	1.37	1.36	1.35	1.34	1.33	1.32
0.27	1.40	1.40	1.39	1.38	1.37	1.36
0.33	1.43	1.42	1.41	1.40	1.39	1.38

Table S8 Viscosities of $[C_8C_1Im][NTf_2]_\chi Co[NTf_2]_2$ ionic liquids from 20-70°C.

	Viscosity / mPa.s					
χ	20 °C	30 °C	40 °C	50 °C	60 °C	70 °C
0	120.1	72.9	47.3	32.4	23.4	17.3
0.1	224.8	126.0	76.6	49.7	34.0	24.3
0.15	335.2	180.2	105.4	66.2	44.1	30.9
0.27	986.9	459.2	239.8	137.3	84.2	55.0
0.33	1695.2	709.8	341.1	183.0	107.3	67.6

Table S9 Densities of $[C_8C_1Im][NTf_2]_\chi Co[NTf_2]_2$ ionic liquids from 20-70°C.

	Density / g mL^{-1}					
χ	20 °C	30 °C	40 °C	50 °C	60 °C	70 °C

0	1.32	1.32	1.31	1.30	1.29	1.28
0.1	13.87	1.38	1.37	1.36	1.35	1.34
0.15	1.42	1.41	1.40	1.39	1.38	1.37
0.27	1.50	1.49	1.48	1.46	1.45	1.44
0.33	1.54	1.53	1.52	1.51	1.50	1.49

Table S10 Viscosities of $[C_8C_1Im][NTf_2]_{0.33}M[NTf_2]_2$ ionic liquids from 20-70°C.

	Viscosity / mPa.s					
M	20 °C	30 °C	40 °C	50 °C	60 °C	70 °C
Li	710.6	351.0	190.5	112.8	71.5	48.0
Ni	1137.7	505.5	255.2	143.1	86.7	56.1
Zn	1490.6	642.8	317.2	172.5	102.1	64.8
Co	1695.2	709.8	341.1	183.0	107.3	67.6
Mg	2263.8	914.9	430.2	225.7	129.6	80.0

Table S11 Densities of $[C_8C_1Im][NTf_2]_{0.33}M[NTf_2]_2$ ionic liquids from 20-70°C.

	Density / g mL^{-1}					
M	20 °C	30 °C	40 °C	50 °C	60 °C	70 °C
Li	1.43	1.42	1.41	1.40	1.39	1.38
Mg	1.50	1.49	1.48	1.47	1.46	1.45
Ni	1.53	1.52	1.51	1.50	1.49	1.48
Co	1.54	1.53	1.52	1.51	1.50	1.49
Zn	1.55	1.53	1.52	1.51	1.50	1.49

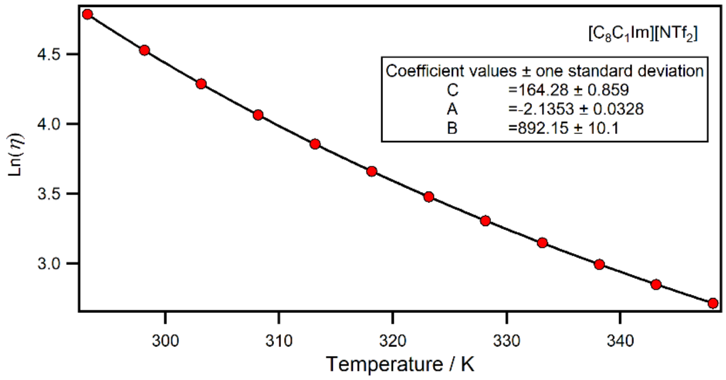

Figure S202 VFT fit from Equation 1 of viscosity data for $[C_8C_1Im][NTf_2]$.

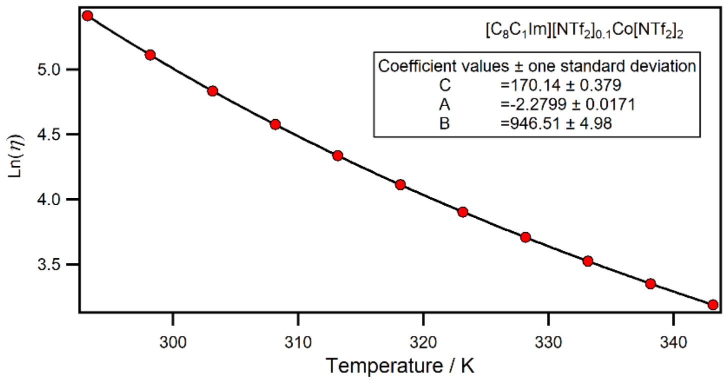

Figure S203 VFT fit from Equation 1 of viscosity data for $[C_8C_1Im][NTf_2]_{0.1}Co[NTf_2]_2$.

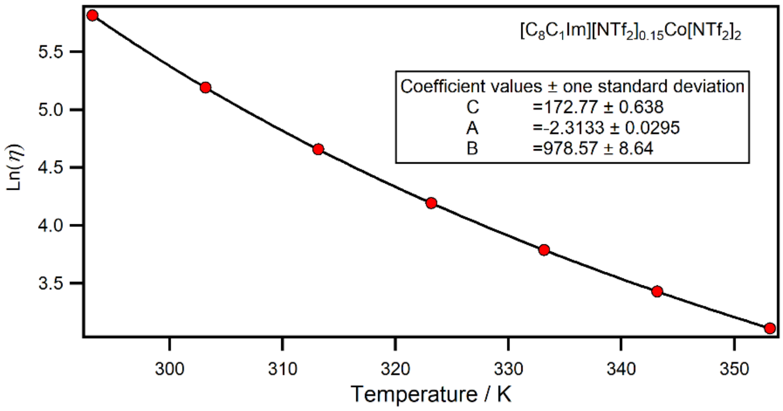

Figure S204 VFT fit from Equation 1 of viscosity data for $[C_8C_1Im][NTf_2]_{0.15}Co[NTf_2]_2$.

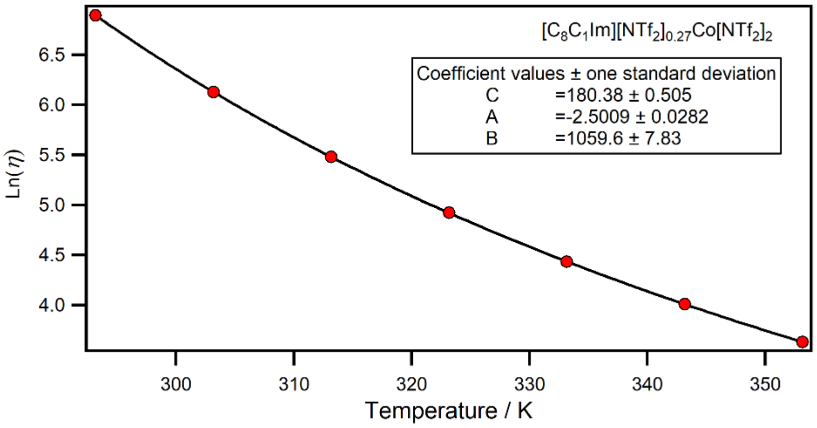

Figure S205 VFT fit from Equation 1 of viscosity data for $[C_8C_1Im][NTf_2]_{0.27}Co[NTf_2]_2$.

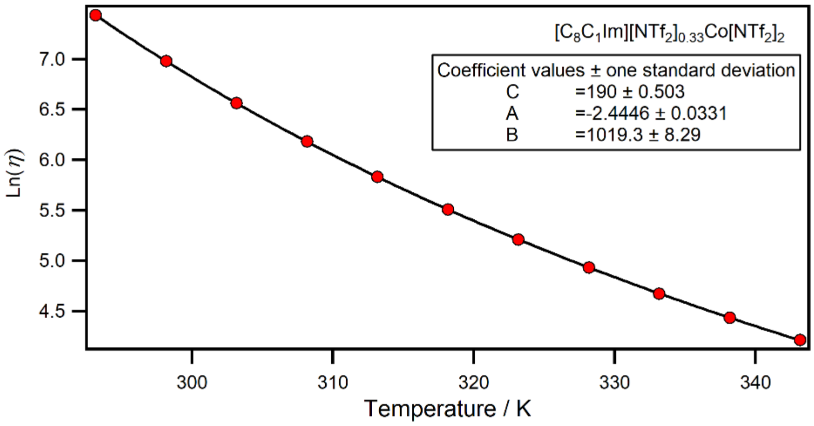

Figure S206 VFT fit from Equation 1 of viscosity data for $[C_8C_1Im][NTf_2]_{0.33}Co[NTf_2]_2$.

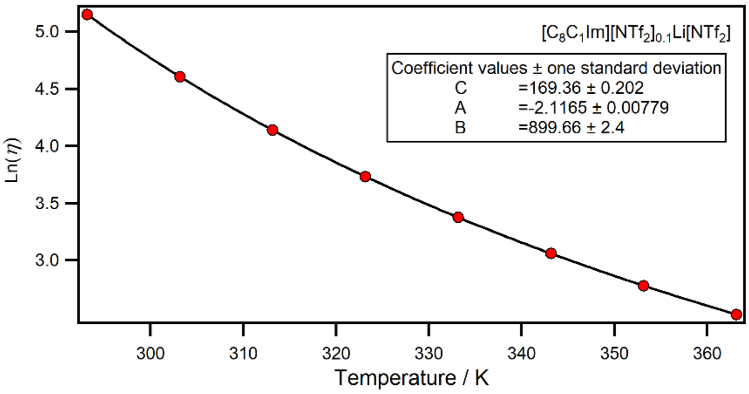

Figure S207 VFT fit from Equation 1 of viscosity data for $[C_8C_1Im][NTf_2]_{0.1}Li[NTf_2]$.

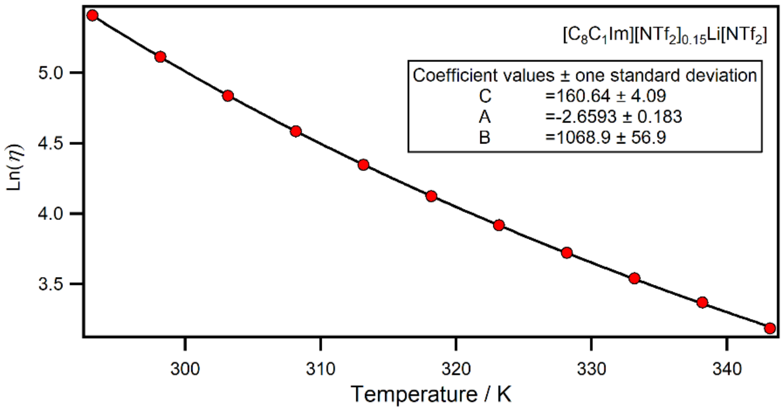

Figure S208 VFT fit from Equation 1 of viscosity data for $[C_8C_1Im][NTf_2]_{0.15}Li[NTf_2]$.

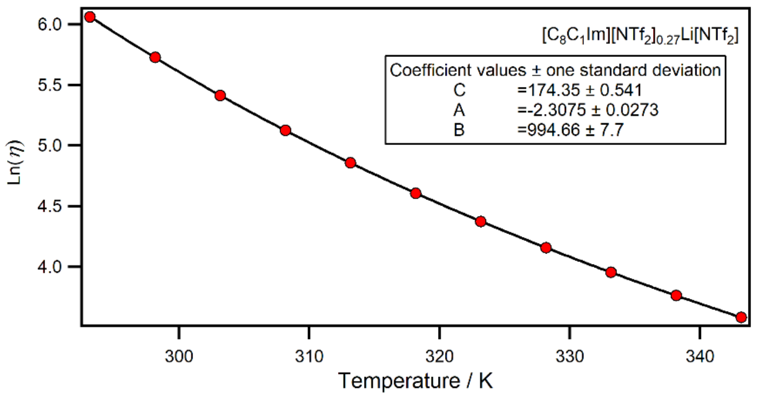

Figure S209 VFT fit from Equation 1 of viscosity data for $[C_8C_1Im][NTf_2]_{0.27}Li[NTf_2]$.

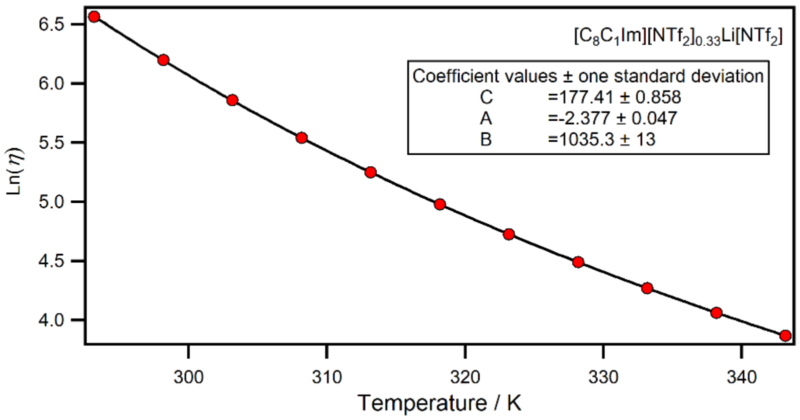

Figure S210 VFT fit from Equation 1 of viscosity data for $[C_8C_1Im][NTf_2]_{0.33}Li[NTf_2]$.

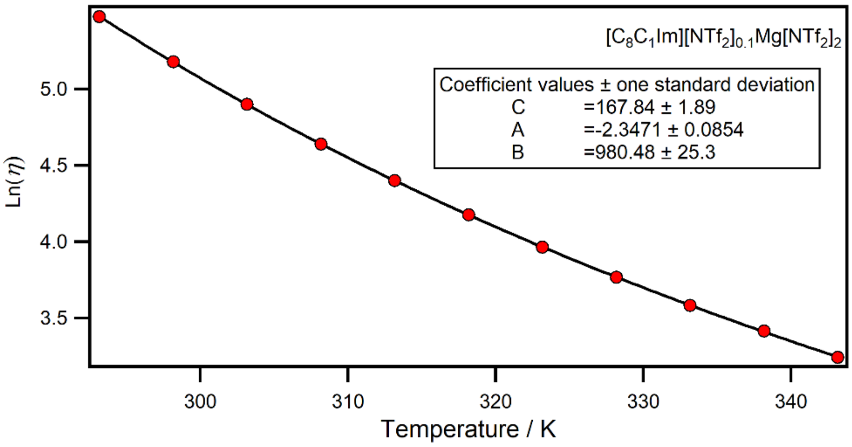

Figure S211 VFT fit from Equation 1 of viscosity data for $[C_8C_1Im][NTf_2]_{0.1}Mg[NTf_2]_2$.

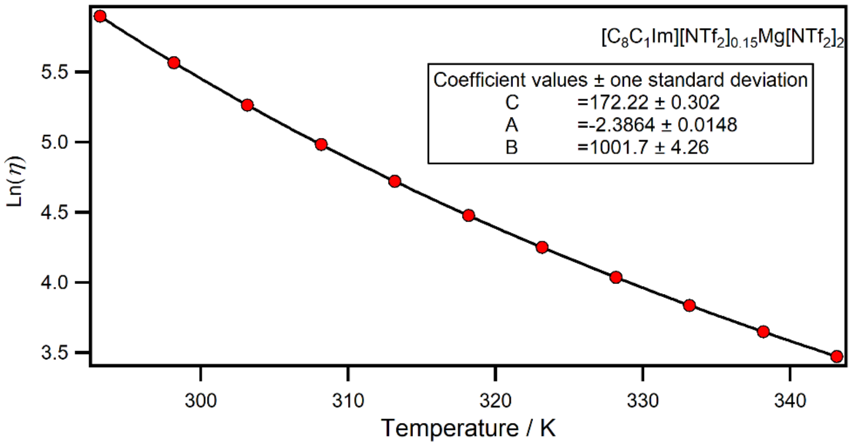

Figure S212 VFT fit from Equation 1 of viscosity data for $[C_8C_1Im][NTf_2]_{0.15}Mg[NTf_2]_2$.

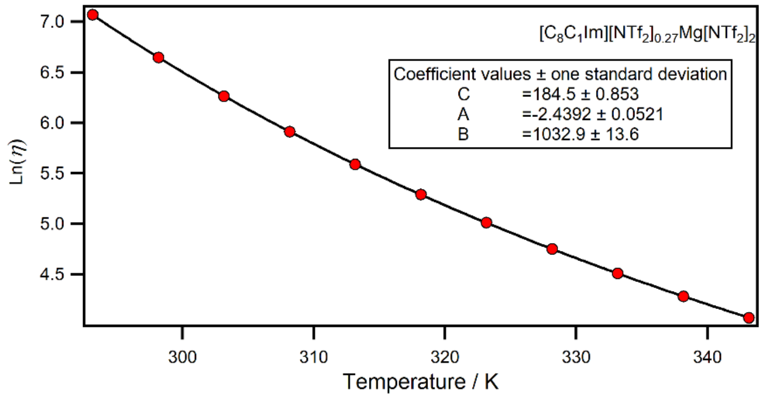

Figure S213 VFT fit from Equation 1 of viscosity data for $[C_8C_1Im][NTf_2]_{0.27}Mg[NTf_2]_2$.

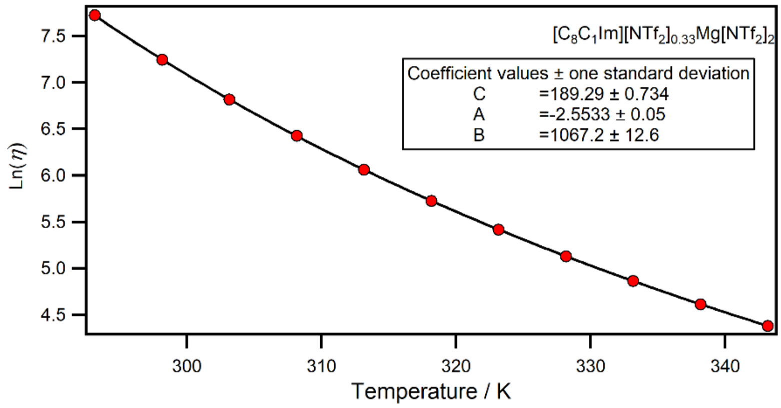

Figure S214 VFT fit from Equation 1 of viscosity data for $[C_8C_1Im][NTf_2]_{0.33}Mg[NTf_2]_2$.

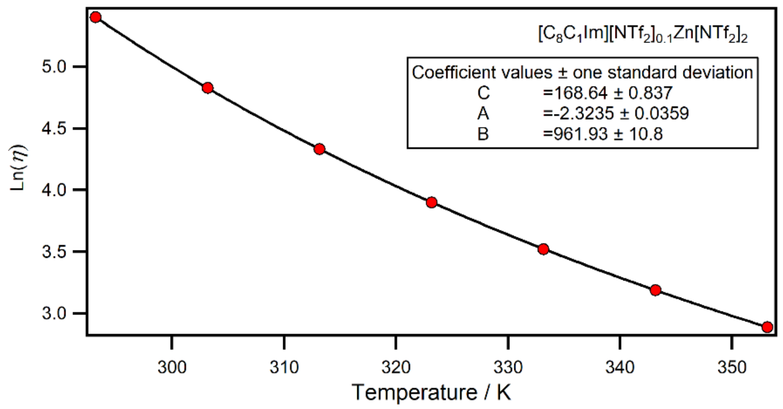

Figure S215 VFT fit from Equation 1 of viscosity data for $[C_8C_1Im][NTf_2]_{0.1}Zn[NTf_2]_2$.

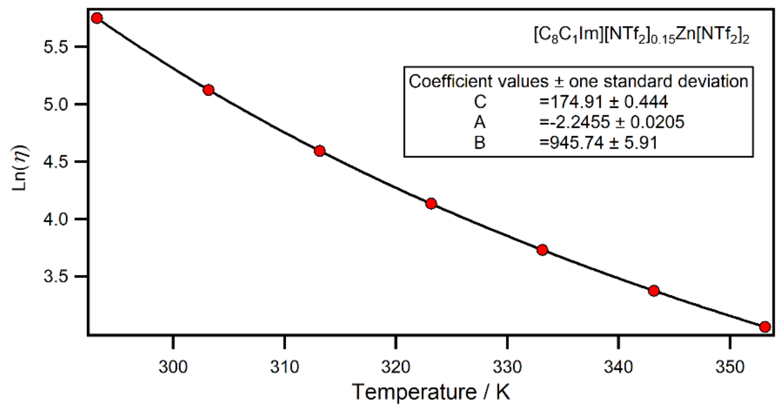

Figure S216 VFT fit from Equation 1 of viscosity data for $[C_8C_1Im][NTf_2]_{0.15}Zn[NTf_2]_2$.

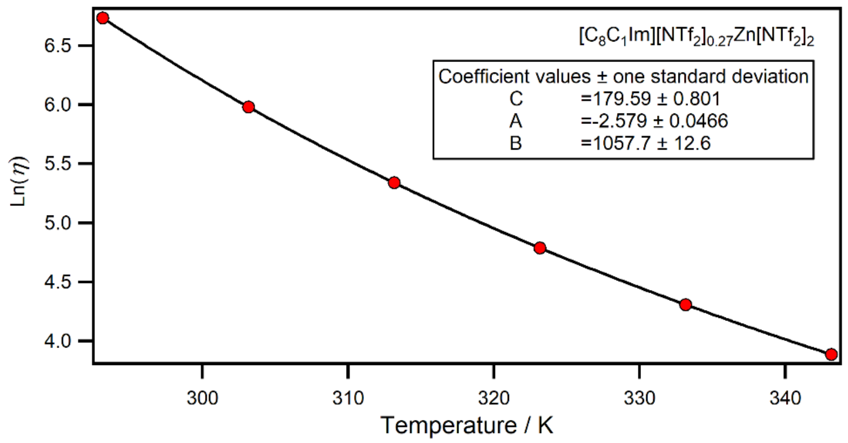

Figure S217 VFT fit from Equation 1 of viscosity data for $[C_8C_1Im][NTf_2]_{0.27}Zn[NTf_2]_2$.

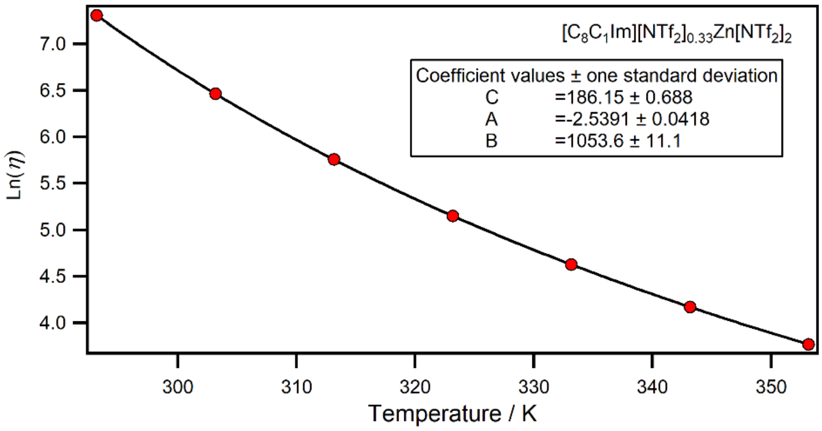

Figure S218 VFT fit from Equation 1 of viscosity data for $[C_8C_1Im][NTf_2]_{0.33}Zn[NTf_2]_2$.

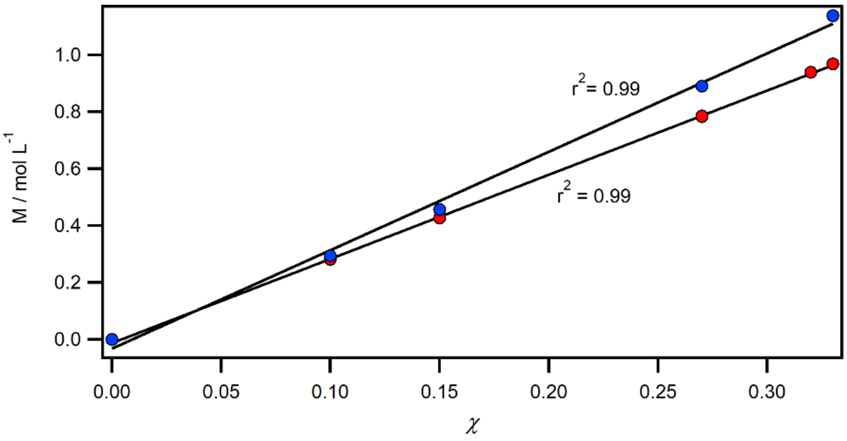

Figure S219 Concentration against mole fraction for +1 (blue) and +2 (red) MILs.

Table S12 Calculated molecular volumes (V_m) and parameters A_η, B, and T_O calculated from optimised VFT fitting of viscosity data. Angell's strength parameter is D = $K_\eta/T_{O.}$

									Optimised VFT Parameters								
					T_g				A_η	B	T_O						
Cation	Anion	Metal	χ	M / mol L^{-1}	°C	K	V_m	η_{exp} (25 °C)	mPa.s	K	K	°C	T_g/T_0	D	η_{calc} (25 °C)	ρ / g mL^{-1}	$\Delta\eta_{(calc-exp)}$
$[C_8C_1Im]$	$[NTf_2]$		0	0.00	-86.0	187.1	358.5	92.642	0.12	892	164.3	-108.9	1.14	5.43	92.7	1.32	0.0
		$Zn[NTf_2]_2$	0.1	0.28			355.5		0.10	962	168.6	-104.5			164.7	1.38	
			0.15	0.43	-74.3	198.8	353.9		0.12	892	174.9	-98.2	1.14	5.41	227.8	1.42	
			0.27	0.78	-65.3	207.8	350.2		0.11	946	179.6	-93.6	1.16	5.89	568.1	1.50	
			0.33	0.97	-61.6	211.5	348.3		0.08	1058	186.2	-87.0	1.14	5.66	961.1	1.54	
		$Co[NTf_2]_2$	0.1	0.28	-78.3	194.8	355.2	166.17	0.08	1054	170.1	-103.0	1.15	5.56	166.4	1.38	0.2
			0.15	0.43	-74.0	199.2	353.5		0.10	947	172.8	-100.4	1.15	5.66	242.6	1.41	
			0.27	0.78	-64.6	208.5	349.4		0.10	979	180.4	-92.8	1.16	5.87	662.7	1.49	
			0.33	0.97	-61.3	211.8	347.4	1075.9	0.08	1060	190.0	-83.1	1.11	5.36	1075.2	1.54	-0.7
		$Mg[NTf_2]_2$	0.1	0.28	-78.5	194.6	356.0	177.48	0.09	1019	167.8	-105.3	1.16	5.84	177.2	1.37	-0.3
			0.15	0.43	-73.6	199.5	354.7	261.64	0.10	980	172.2	-100.9	1.16	5.82	261.9	1.40	0.3
			0.27	0.78	-64.3	208.8	351.7	769.53	0.09	1002	184.5	-88.6	1.13	5.60	772.2	1.47	2.7
			0.32	0.94	-58.9	214.2	350.4	1400.6	0.09	1033	189.3	-83.9	1.13	5.64	1408.3	1.50	7.7
		$Li[NTf_2]$	0.1	0.29	-79.5	193.6	340.0		0.08	1067	169.4	-103.8	1.14	5.31	130.2	1.35	
			0.15	0.46	-78.7	194.4	330.8	166.52	0.12	900	160.6	-112.5	1.21	6.65	166.3	1.36	-0.2
			0.27	0.89	-71.7	201.4	308.6	307.59	0.07	1069	174.4	-98.8	1.16	5.70	307.0	1.40	-0.6
			0.33	1.14	-67.2	205.9	297.5	491.76	0.10	995	177.4	-95.7	1.16	5.84	491.6	1.42	-0.2

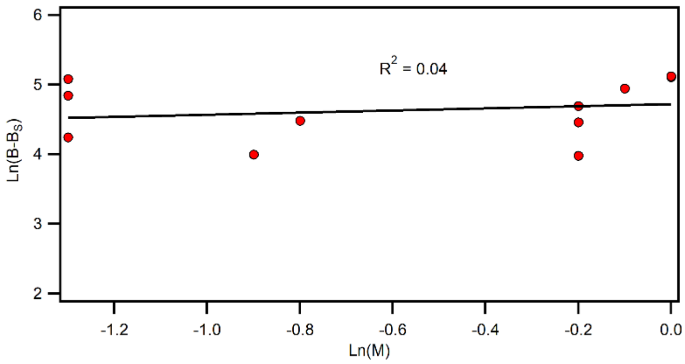

Figure S220 Correlations of Ln(B-B_S) vs Ln(M) where B is the pseudo-activation energy parameter of the MIL solution and B_S is the same parameter for pure IL. Values were derived from VFT fittings and are plotted against Ln(M) which is the natural logarithm of the molar concentration in g L^{-1}.

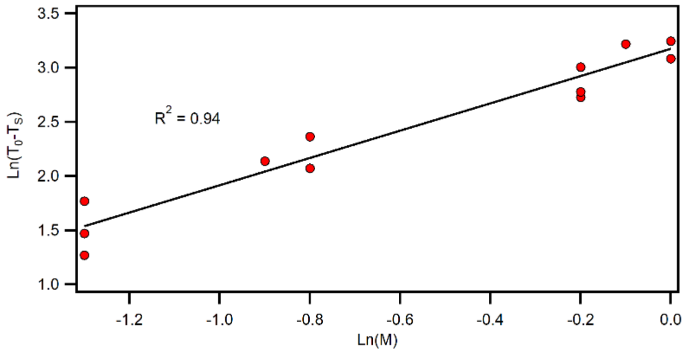

Figure S221 Correlations of Ln(T_0-T_S) where T is the Vogel temperature of the MIL solution and B_S is the same parameter for pure IL. Values were derived from VFT fittings and are plotted against Ln(M) which is the natural logarithm of the molar concentration in g L^{-1}.

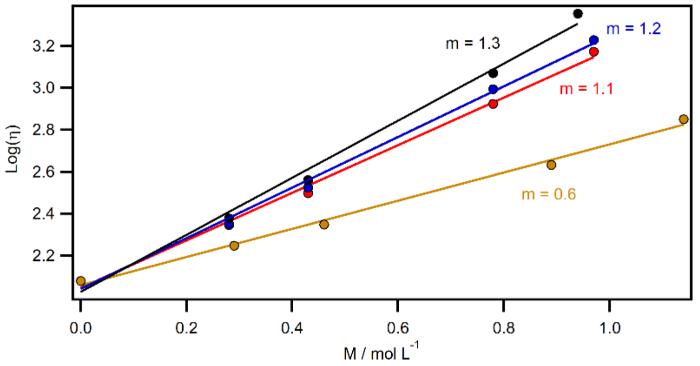

Figure S222 Logarithm of viscosity against molar concentration for $[C_8C_1Im][NTf_2]_xM[NTf_2]_n$ where M = Li (yellow), Zn (red), Co (blue), and Mg (black).

Heat Capacity Data

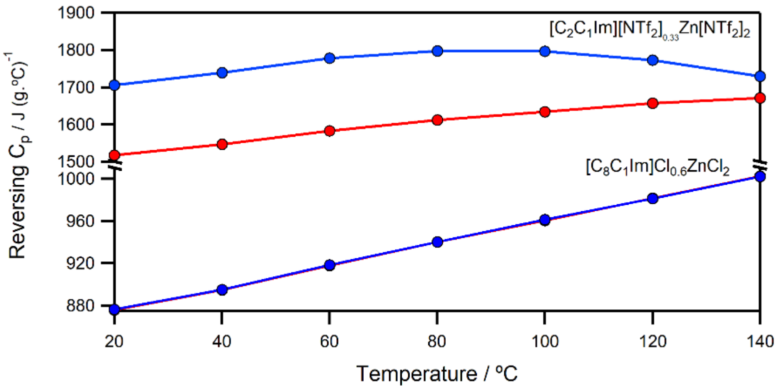

Figure S223 QI-MDSC heat capacities for $[C_2C_1Im][NTf_2]_{0.33}Zn[NTf_2]_2$ and a chlorozincate IL with excess ZnCl2.

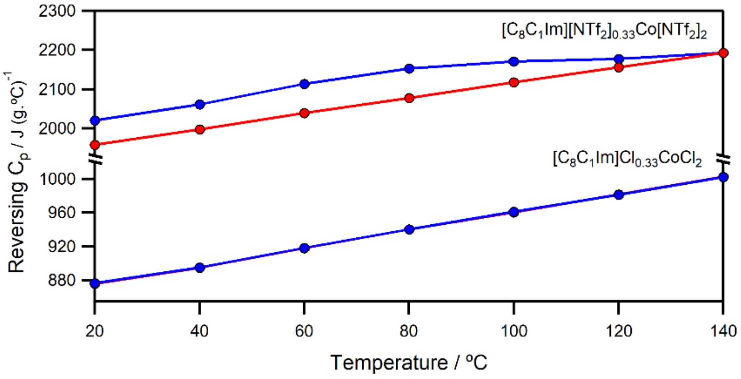

Figure S224 QI-MDSC heat capacities for $[C_8C_1Im][NTf_2]_{0.33}Co[NTf_2]_2$ and a cobalt chloride analogue.

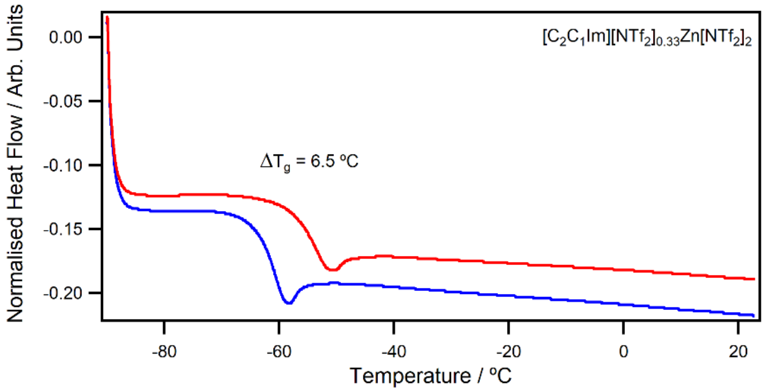

Figure S225 Change in glass transition before (blue) and after (red) QI-MDSC experiments for $[C_2C_1Im][NTf_2]_{0.33}Zn[NTf_2]_2$.

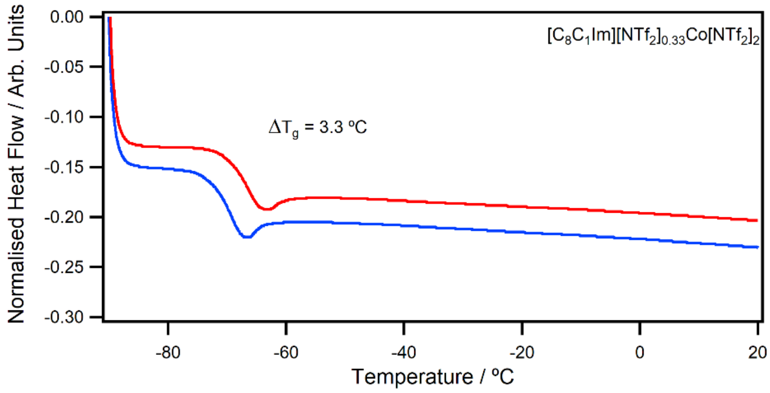

Figure S226 Change in glass transition before (blue) and after (red) QI-MDSC experiments for [C_8C_1Im][NTf_2]$_{0.33}$Co[NTf_2]$_2$.

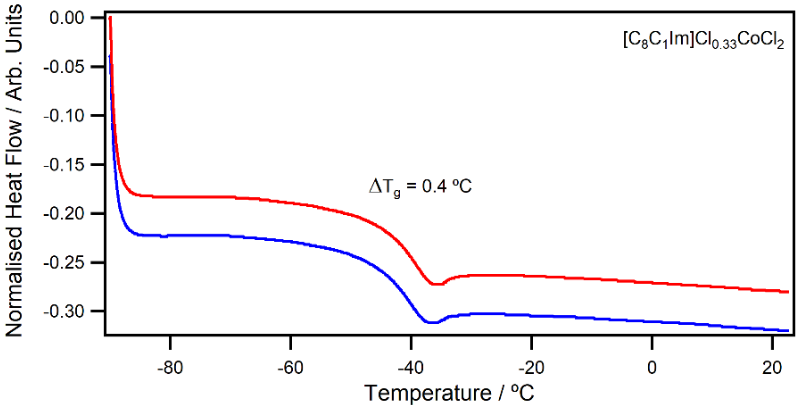

Figure S227 Change in glass transition before (blue) and after (red) QI-MDSC experiments for [C_8C_1Im]$Cl_{0.33}CoCl_2$.

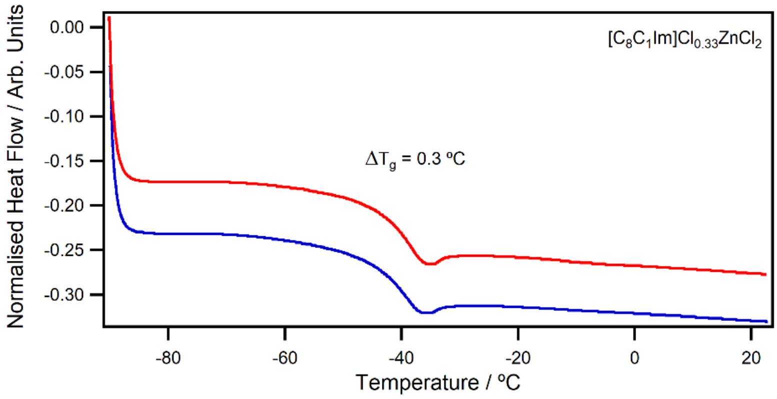

Figure S228 Change in glass transition before (blue) and after (red) QI-MDSC experiments for [C_8C_1Im]$Cl_{0.33}ZnCl_2$.

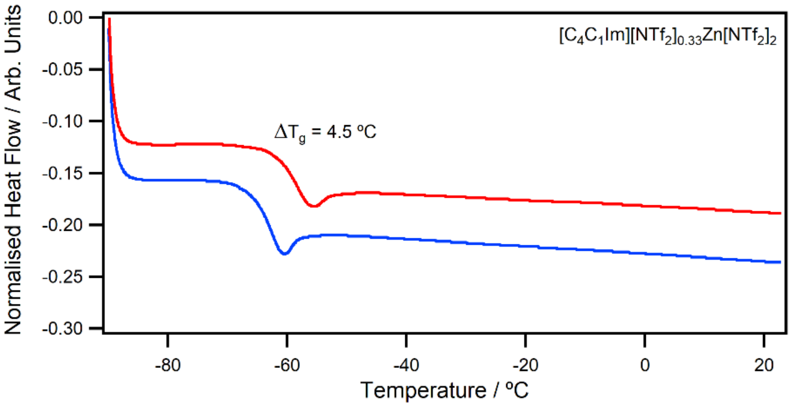

Figure S229 Change in glass transition before (blue) and after (red) QI-MDSC experiments for $[C_4C_1Im][NTf_2]_{0.33}Zn[NTf_2]_2$.

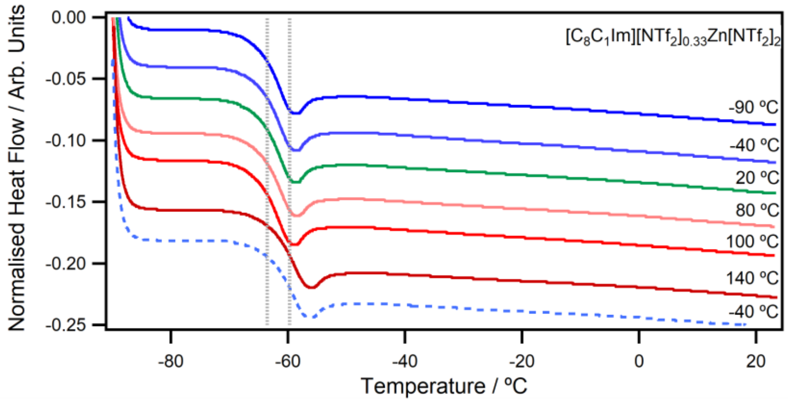

Figure S230 Annealing experiments for $[C_8C_1Im][NTf_2]_{0.33}Zn[NTf_2]_2$ held at temperature intervals for 60 minutes.

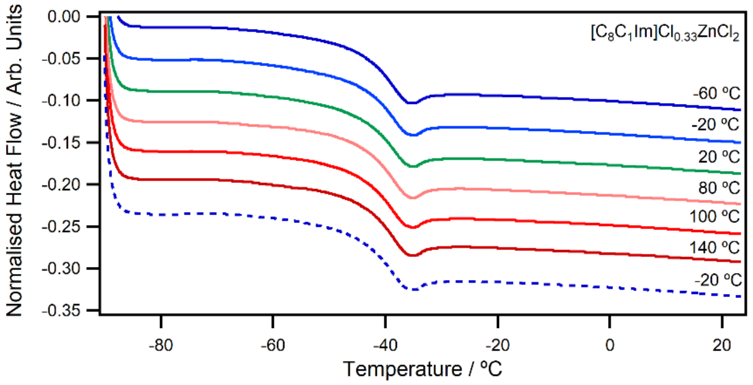

Figure S231 Annealing experiments for $[C_8C_1Im]Cl_{0.33}ZnCl_2$ held at temperature intervals for 60 minutes.

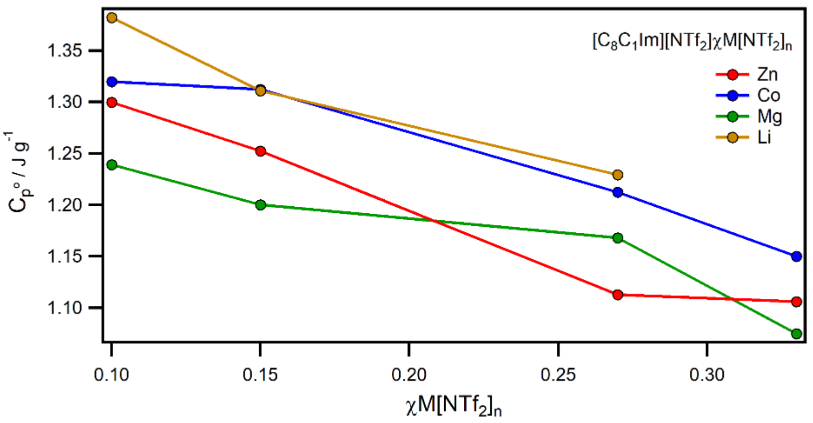

Figure S232 Heat capacities of $[C_8C_1Im][NTf_2]_{\chi}M[NTf_2]_n$ MILs measured from QI-MDSC experiments at 20°C.

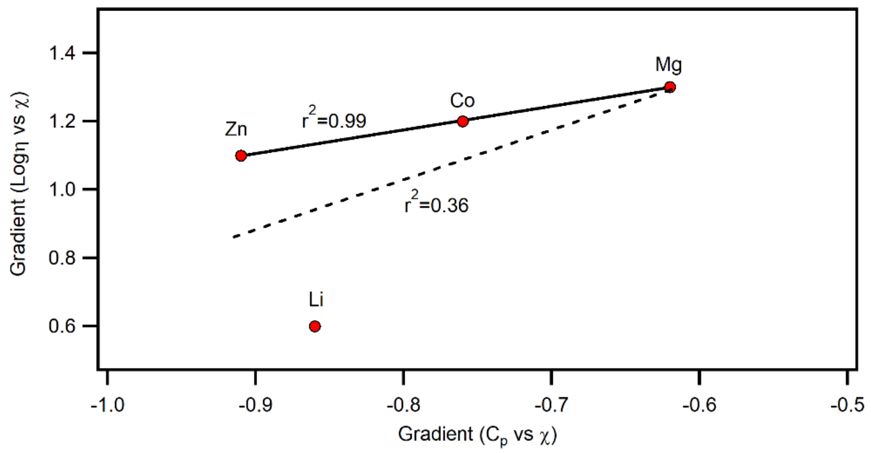

Figure S233 Correlations of gradients taken from linear fits of logarithm of viscosity vs heat capacity against mole fraction of metal salts, with a fit to all (dashed) or only divalent metals (solid).

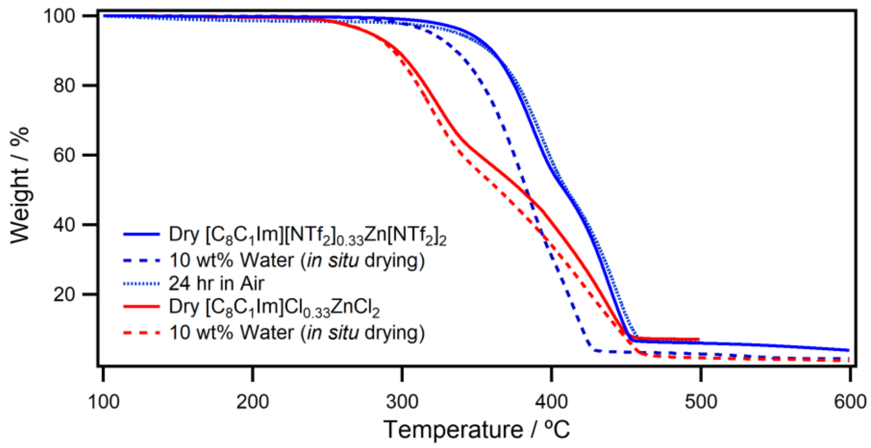

Figure S234 TGA thermograms for pure $[C_8C_1Im][NTf_2]_{0.33}Zn[NTf_2]_2$ (blue solid) and a 10% water mixture (blue dashed) compared to analogous $[C_8C_1Im]Cl_{0.33}ZnCl_2$ MIL.

3. References

1 L. Bui-Le, C. J. Clarke, A. Bröhl, A. P. S. Brogan, J. A. J. Arpino, K. M. Polizzi and J. P. Hallett, *Commun Chem*, 2020, **3**, 1–9.

2 C. J. Clarke, L. Bui-Le and J. Hallett, *Analytical Methods*, 2020, **12**, 2244–2252.

3 C. Clarke, S. Hayama, A. Hawes, J. Hallett, T. Chamberlain, K. Lovelock and N. Besley, *J Phys Chem A*, **123**, 9552–9559.

4 (PDF) Vogel-Fulcher-Tammann behavior for Viscosity, https://www.researchgate.net/publication/378184224_Vogel-Fulcher-Tammann_behavior_for_Viscosity?channel=doi&linkId=65cc9fa179007454978f99f6&showFulltext=true, (accessed 30 May 2024).

5 J. M. Slattery, C. Daguenet, P. J. Dyson, T. J. S. Schubert, I. Krossing, J. M. Slattery, I. Krossing, C. Daguenet, J. Dyson and T. J. S. Schubert, *Angewandte Chemie International Edition*, 2007, **46**, 5384–5388.

6 Y. Marcus, *J Mol Liq*, 2015, **209**, 289–293.

7 C. N. Singman, *J Chem Educ*, 1984, **61**, 137–142.

8 G. Li and C. W. Monroe, *Front Energy Res*, 2021, **9**, 660081.
